# Artifact-free whole-slide imaging with structured illumination microscopy and Bayesian image reconstruction

**DOI:** 10.1093/gigascience/giaa035

**Published:** 2020-04-14

**Authors:** Karl A Johnson, Guy M Hagen

**Affiliations:** UCCS BioFrontiers Center, University of Colorado at Colorado Springs, 1420 Austin Bluffs Parkway, Colorado Springs, CO 80918, USA

**Keywords:** structured illumination microscopy, SIM, image stitching, Bayesian methods, MAP-SIM, SIMToolbox, histopathology, cancer

## Abstract

**Background:**

Structured illumination microscopy (SIM) is a method that can be used to image biological samples and can achieve both optical sectioning and super-resolution effects. Optimization of the imaging set-up and data-processing methods results in high-quality images without artifacts due to mosaicking or due to the use of SIM methods. Reconstruction methods based on Bayesian estimation can be used to produce images with a resolution beyond that dictated by the optical system.

**Findings:**

Five complete datasets are presented including large panoramic SIM images of human tissues in pathophysiological conditions. Cancers of the prostate, skin, ovary, and breast, as well as tuberculosis of the lung, were imaged using SIM. The samples are available commercially and are standard histological preparations stained with hematoxylin-eosin.

**Conclusion:**

The use of fluorescence microscopy is increasing in histopathology. There is a need for methods that reduce artifacts caused by the use of image-stitching methods or optical sectioning methods such as SIM. Stitched SIM images produce results that may be useful for intraoperative histology. Releasing high-quality, full-slide images and related data will aid researchers in furthering the field of fluorescent histopathology.

## Data Description

### Context

Structured illumination microscopy (SIM) is a method in optical fluorescence microscopy that achieves both optical sectioning (OS-SIM) [1] and resolution beyond the diffraction limit (SR-SIM) [2, 3]. SIM has been used for super-resolution imaging of both fixed and live cells [4–7] and has matured enough as a method that it is now available commercially. In SIM, a set of images is acquired using an illumination pattern that shifts between each image. As SIM has developed, diverse strategies have been proposed for creation of the SIM pattern [1, 8–13]. Several different approaches for processing the data have also been introduced [3, 7, 8, 14–16].

Recently, microscope systems capable of imaging with high resolution and a large field of view (FOV) have been developed [17–21], some using custom-made microscope objectives. However, stitching together images acquired with a higher magnification objective to create a large mosaic remains a valid and popular approach. Some published results involving stitched images are hindered by pronounced artifacts in which the edges of the individual sub-images are visible, usually as dark bands that outline each sub-image [22–24]. On the other hand, several studies have proposed methods for stitching of microscope images with reduced artifacts [25–32].

The combination of SIM with image-stitching methods allows collection of large FOV images with both optical sectioning and super-resolution properties. Here, we demonstrate methods and provide complete datasets for 5 different samples. The samples are hematoxylin-eosin (H&E) stained histological specimens that provide examples of human diseases (ovarian cancer, breast cancer, prostate cancer, skin cancer, and tuberculosis) and that are also available commercially for those who wish to reproduce our work. We used freely available optical designs [6, 10, 33] and open source software [33] for SIM imaging, along with freely available software for image stitching (Microsoft Image Composite Editor [ICE] [34], or a well-validated plug-in [26] for ImageJ [35]). Combining this with devignetting methods, we produced stitched images that are free of noticeable artifacts from stitching or from SIM reconstruction.

Fluorescence microscopy is becoming more important in histopathology. Traditional bright-field microscopy diagnostic methods require a time-consuming process, involving chemical fixation and physical sectioning. The use of optical sectioning fluorescence microscopy allows high-quality images to be captured without the need for physical sectioning. Consequently, it has been shown that imaging can be performed on large human tissue samples within 1 hour after excision [36]. Additionally, other studies have shown the results of fluorescence imaging to be usable and accurate in diagnosis of various medical conditions [37–42]. Previously, it was noted that obvious stitching artifacts significantly decrease the usability of large fluorescence images in medical diagnosis. In 1 case, such artifacts resulted in the rejection of more than half of the images acquired [38]. The set-up we describe here allows for fast, artifact-free, high-resolution imaging of fluorescent samples and is compatible with samples stained with most fluorescent dyes.

## Methods

### Samples

All samples used in this study are available from Carolina Biological (Burlington, NC, USA), Omano (Chongqing, China), or Ward's Science (Rochester, NY, USA). The samples are ∼7 μm thick and are stained with H&E. The commercial source, product number, and other SIM imaging parameters for each sample are detailed in Table 1. Table 2 details imaging parameters for acquisitions of each sample with a color camera.

**Table 1:** Imaging parameters for the SIM datasets

Sample	Source company and product No.	SIM pattern No. of phases	Exposure time, ms	No. of tiles	Objective magnification/NA	Acquisition time, s	Stitching software
Carcinoma of prostate	Carolina, 318,492	5	50	23 × 11	20×/0.45	315	Microsoft ICE
Basal cell carcinoma	Ward's Science, 470,183–256	6	75	29 × 18	30×/1.05	821	FIJI
Adenocarcinoma of ovary	Carolina, 318,628	5	100	25 × 14	10×/0.4	595	Microsoft ICE
Adenocarcinoma of breast	Carolina, 318,766	8	200	12 × 8	10×/0.4	278	FIJI
Lung tuberculosis	Omano, OMSK-HP50	5	100	20 × 16	30×/1.05	541	FIJI

NA: numerical aperture.

**Table 2:** Parameters for the color images

Sample	No. of tiles	Objective magnification/NA
Carcinoma of prostate	6 × 5	4×/0.16
Basal cell carcinoma	5 × 5	4×/0.16
Adenocarcinoma of ovary	11 × 11	4×/0.16
Adenocarcinoma of breast	6 × 6	4×/0.16
Lung tuberculosis	8 × 10	10×/0.4

NA: numerical aperture.

### Microscope set-up and data acquisition

**Figure 1:**
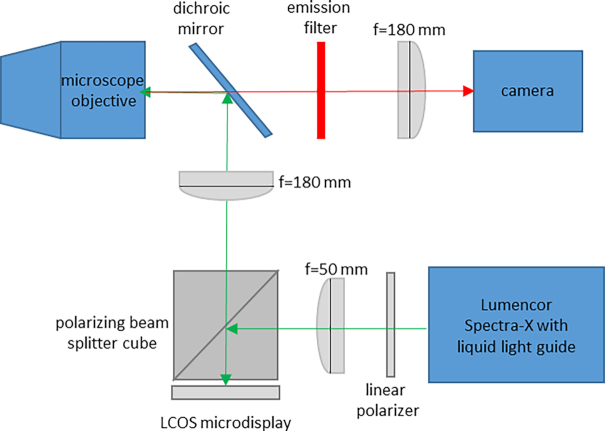
Simplified diagram of SIM system. LCOS, liquid crystal on silicon.

We used a home-built SIM set-up based on the same design as described previously [6, 10, 15] (Fig. 1). The SIM system is based on an IX83 microscope (Olympus, Tokyo, Japan) equipped with a Zyla 4.2+ sCMOS camera (Andor, Belfast, UK) under the control of IQ3 software (Andor). We used the following Olympus objectives: UPLSAPO 4×/0.16 numerical aperture (NA), UPLSAPO 10×/0.4 NA, LUCPLFLN 20×/0.45 NA, and UPLSAPO 30×/1.05 NA silicone oil immersion. For color images we used an aca1920–40uc color camera (Basler, Ahrensburg, Germany) under control of Pylon software (Basler). We used an MS-2000 motorized microscope stage (Applied Scientific Instrumentation, Eugene, OR, USA) to acquire tiled SIM images. In all datasets, the stage scanning was configured such that all image edges overlapped by 20%.

Briefly, the SIM system uses a ferroelectric liquid crystal on silicon (LCOS) microdisplay (type SXGA-3DM, Forth Dimension Displays, Dalgety Bay, Fife, UK). This device has been used previously in SIM and related methods in fluorescence microscopy [5, 10, 15, 33, 43–47] and allows one to produce patterns of illumination on the sample that can be reconfigured at will by changing the image displayed on the device. The light source (Lumencor Spectra-X, Beaverton, OR, USA) is toggled off between SIM patterns and during camera readout. Fig. 1 shows a simplified diagram of the microscope system.

### Motorized stage and illumination control

**Figure 2:**
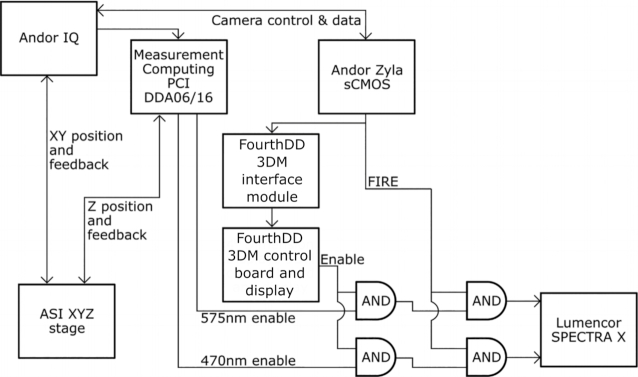
Simplified connection diagram.

Close synchronization between the camera acquisitions, light source, and microdisplay ensures rapid image acquisition, helps reduce artifacts, and reduces light exposure to the sample. As shown in Fig. 2, Andor IQ software controls the SIM system.

**Figure 3:**
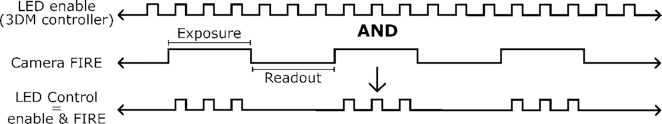
Illumination trigger signal logic. This logical operation occurs for each illumination wavelength at the rightmost AND gates pictured in Fig. 2.

While the camera and XYZ stage receive signals directly from Andor IQ software and an input/output computer card (Measurement Computing DDA06/16, Contoocook, NH, USA), the illumination signals generated by the software must be altered before being sent to the light source. First, the microdisplay used in our set-up will not produce an image on the sample if it is illuminated with a constant light source. Rather, a meaningful illumination pattern will only form if the light source is synchronized with an enable signal output from the microdisplay control board. Therefore, the channel signals output by IQ are first modulated with the microdisplay enable signal (this is performed by the leftmost AND gates pictured in Fig. 2). Additionally, to reduce unnecessary light exposure to the sample, the light source is shut off whenever the camera sensor is not being exposed. This is accomplished by performing a second logical AND of the result of the previous AND with the “FIRE” signal output from the camera. This process is illustrated in Fig. 3.

### SIM data processing

SIM reconstructions were performed in the same way as previously described using SIMToolbox, an open source and freely available program that our group developed for processing SIM data [33]. We generated optically sectioned, enhanced resolution images using a Bayesian estimation method, maximum a posteriori probability SIM (MAP-SIM) [15]. MAP-SIM works using maximum a posteriori probability methods, which are well known in microscopy applications [48, 49], to enhance high spatial frequency image information. We then combine this information, in the frequency domain, with low spatial frequency image information obtained by OS-SIM methods, then produce the final image by an inverse Fourier transform [15]. We typically measure the final resolution obtained by analyzing the frequency spectrum of the resulting image, as is discussed below.

The illumination patterns used here are generated such that the sum of all positions in each pattern set results in homogenous illumination. As such, a wide-field (WF) image can be reconstructed from SIM data simply by performing an average intensity projection of the patterned images. This can be described by

$$\begin{equation*}
{\rm{\ }}{I_{\mathrm{WF}}} = \frac{1}{N}{\rm{\ }}\mathop \sum \nolimits_{n{\rm{\ }} = {\rm{\ }}1}^N {I_n},
\end{equation*}$$

where *N* is the number of pattern phases, *I_n_* is the image acquired on the *n*th illumination position, and *I*_WF_ is the WF reconstruction. This is the method we used to generate WF images throughout this study.

### Vignetting correction

Following SIM reconstruction, vignetting artifacts remain in each tile. If not removed prior to stitching, this vignetting introduces a distracting grid pattern in the final stitched image. We performed vignette removal by dividing each tile of the mosaic by an image representing the vignetting profile common to all tiles. Other studies have used an image of a uniformly fluorescent calibration slide as a reference for vignette removal [36], where information concerning non-uniform illumination is captured. However, we found that SIM processing introduces vignetting artifacts beyond those due to non-uniform illumination. Additionally, these artifacts vary somewhat depending on properties of the sample being imaged. As such, performing pre-acquisition calibration on a uniformly fluorescent slide is not sufficient to remove vignetting artifacts from SIM reconstructions. Instead, an estimate of the vignetting profile is found through analysis of the mosaic tiles after SIM reconstruction.

**Figure 4:**
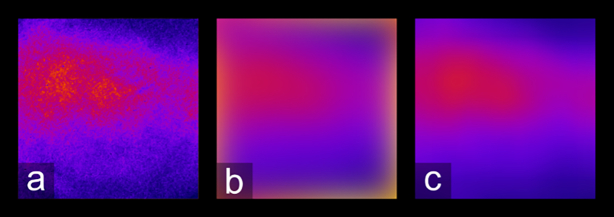
Edge handling during the blurring process. (a) Result after performing an average intensity projection on a set of MAP-SIM tiles. (b) Shows (a) after being blurred using ImageJ's “Gaussian blur” with a radius of 200 pixels, while (c) shows (a) blurred using the “border limited mean” filter.

A blurred average intensity projection of the tiles is a good approximation of the vignetting profile because an average intensity projection merges the tiles into a single image with averaged foreground information while preserving the vignetting profile. Subsequent blurring with an appropriate radius and edge-handling method also eliminates the high spatial frequency foreground without affecting the low spatial frequency illumination profile. To eliminate errors during the blurring step due to the blurring area extending outside the original image, we used an edge-handling method in which the blurring area is reduced near the edges of the image such that no values outside the image border are sampled. Unlike edge-handling methods in which the image is padded with a uniform value (or mirrored and tiled) to accommodate a blurring area that extends beyond the original image limits, this method is free from major artifacts, such as erroneous brightness of the image edges (see Fig. 4). While the average intensity projection removes most foreground information from the images, some coarseness remains in the vignette estimation after this step (Fig. 4a), especially for stitches with <50 tiles. The blurring step serves to eliminate only this non-vignette information and must preserve the illumination profile. As shown in Fig. 4b, use of the default Gaussian blurring function in ImageJ introduces a bright glow near the borders of the image, a significant artifact that does not reflect the original vignetting profile. Use of the “border limited mean” filter, on the other hand, does not introduce this aberration, as shown in Fig. 4c.

**Figure 5:**
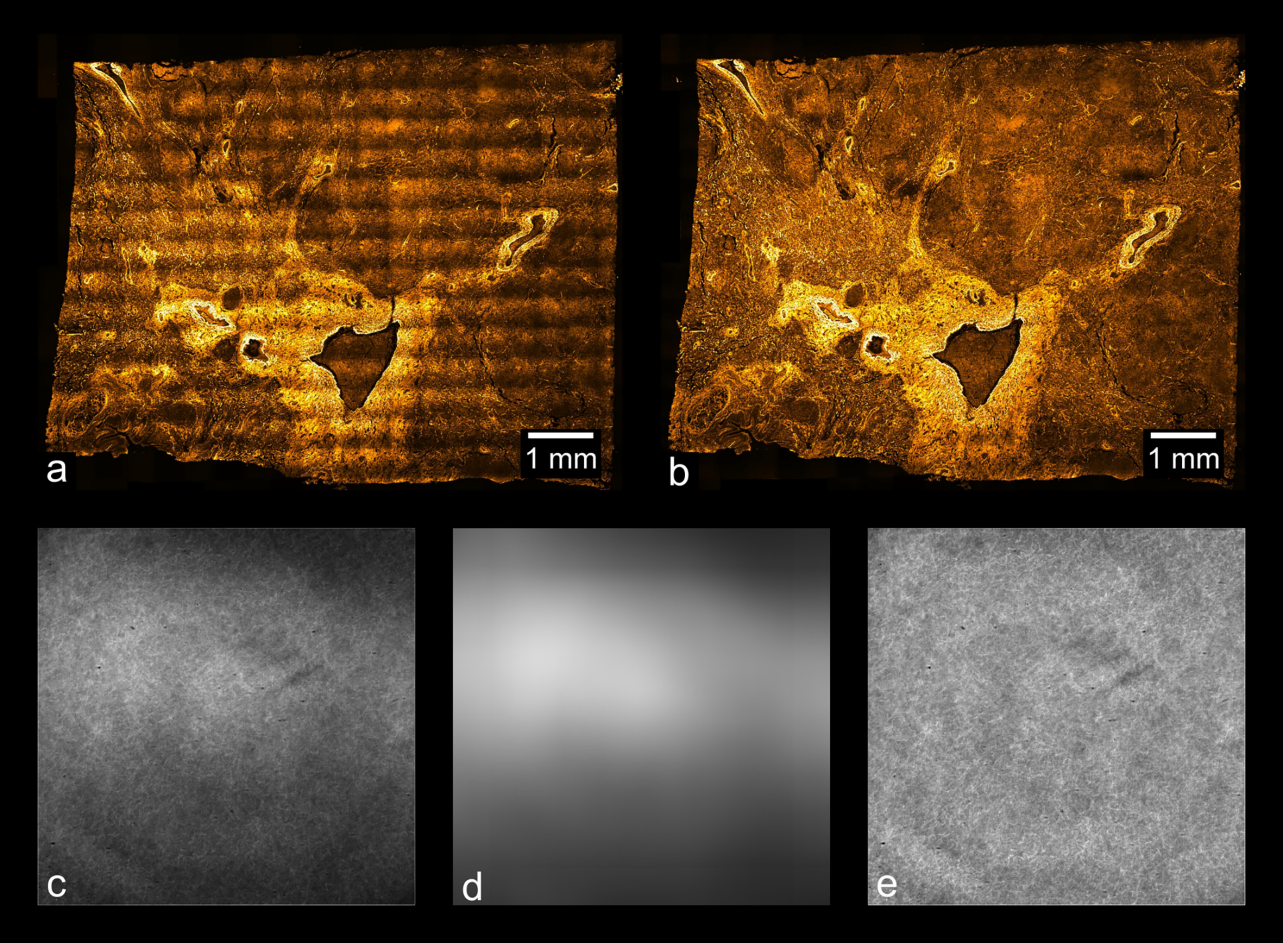
Vignetting artifacts and their removal. (a) Result of stitching images without applying the devignetting process; (b) a stitch of the same data after devignetting has been applied. (c) Average intensity projection of the images used to stitch (a), which estimates the vignette profile of each frame. This estimate can be refined by application of an edge-limited blurring filter, as shown in (d). (e) Average intensity projection of the data used in (b), after devignetting has been applied. The uniform brightness of (e) indicates that no major vignetting artifacts remain in the devignetted data.

Approximating the illumination profile works especially well for histological samples because such samples are non-sparse and require many tiles, factors that improve the accuracy of this approach. We performed all steps of this devignetting process using built-in functions and the “Fast Filters” plug-in in ImageJ [50]. The effect of devignetting is illustrated in Fig. 5.

### Image stitching

With visible vignetting removed, we then stitched together a composite image from the tiles. The pre-processing allows for stitching to be done in various stitching applications; Microsoft ICE and Preibisch's plug-in for FIJI [26] were used to stitch the data presented here.

**Figure 6:**
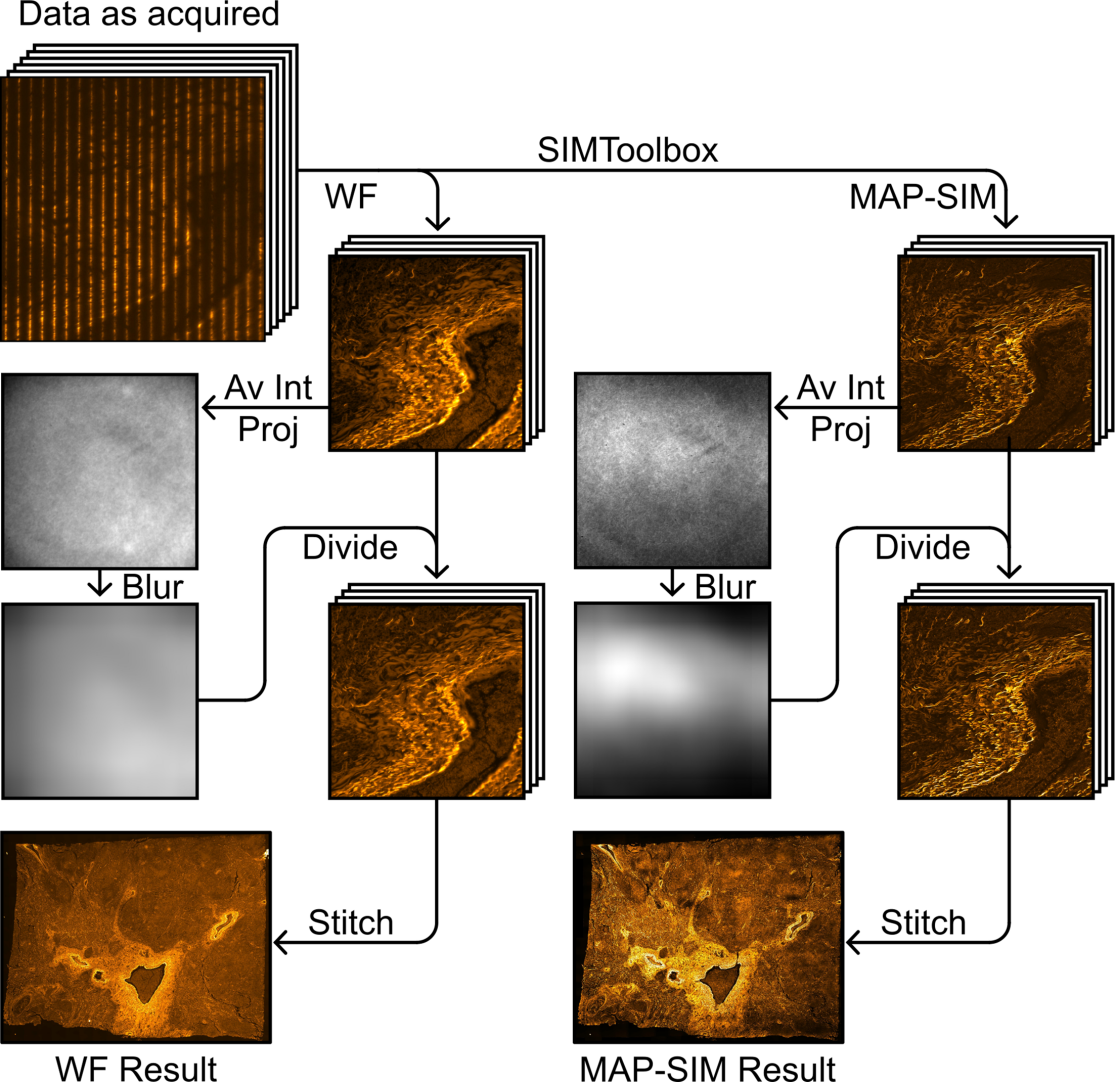
Panoramic SIM data-processing workflow. Devignetting was performed after SIM reconstruction. Note that the vignette profile differs between reconstruction methods, necessitating separate projection, blurring, and division steps. Av Int Proj, average intensity projection.

The data-processing procedure described here is summarized in Fig. 6. The total time for processing each dataset was ∼30 min.

### Color image data-processing methods

We created color overview images by stitching devignetted bright-field acquisitions. Devignetting was performed simply by adding the inverse of an empty bright-field acquisition to each color tile using ImageJ. For this method to produce optimal results, the empty bright-field image must be acquired in conditions identical to those of the raw tile data, such that the illumination profile in the empty image matches that of the unprocessed tiles. This simple operation removes nearly all visible vignetting and color balance artifacts within each tile. The results after devignetting were then stitched using Preibisch's plug-in for FIJI [26].

### Resolution measurement

We evaluated our results by measuring image resolution using SR Measure Toolbox. SR Measure Toolbox [51] measures the resolution limit of input images through analysis of the normalized, radially averaged power spectral density (PSD_ca_) of the images, as previously described [6]. Briefly, the resolution limit in real space is determined by evaluating the cut-off frequency in Fourier space. The cut-off frequency is estimated by calculating the spatial frequency at which the PSD_ca_ (after noise correction) decreases to zero.

**Figure 7:**
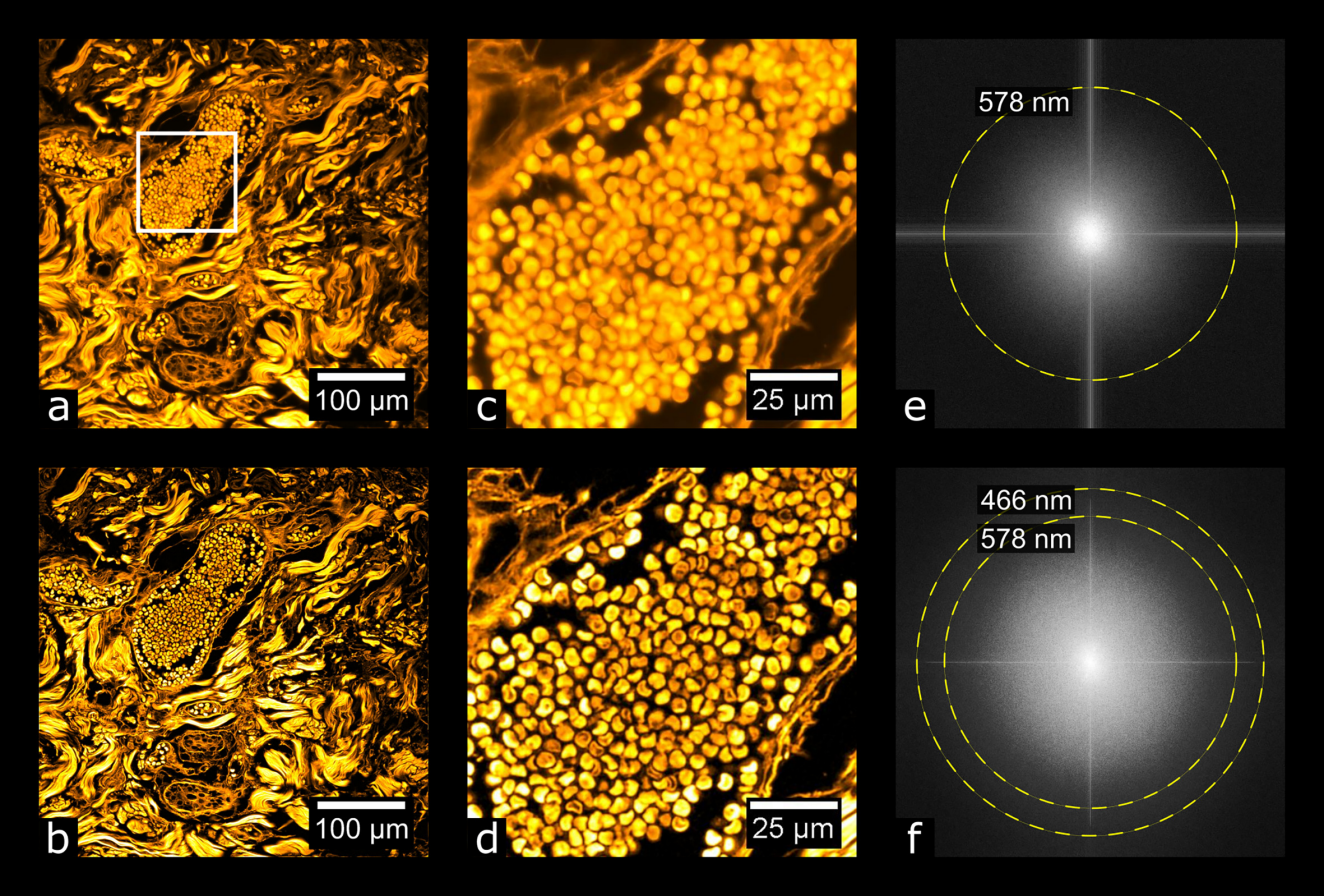
Evaluating image resolution. (a, b) A tile from the data in Fig. 10 (basal cell carcinoma sample) after wide-field and MAP-SIM reconstruction, respectively. (c, d) Zoom-in of (a) and (b), respectively. (e, f) FFT of (a) and (b), respectively. The dotted lines in (e) and (f) indicate the resolution of each image according to the resolution measurement described.

**Figure 8:**
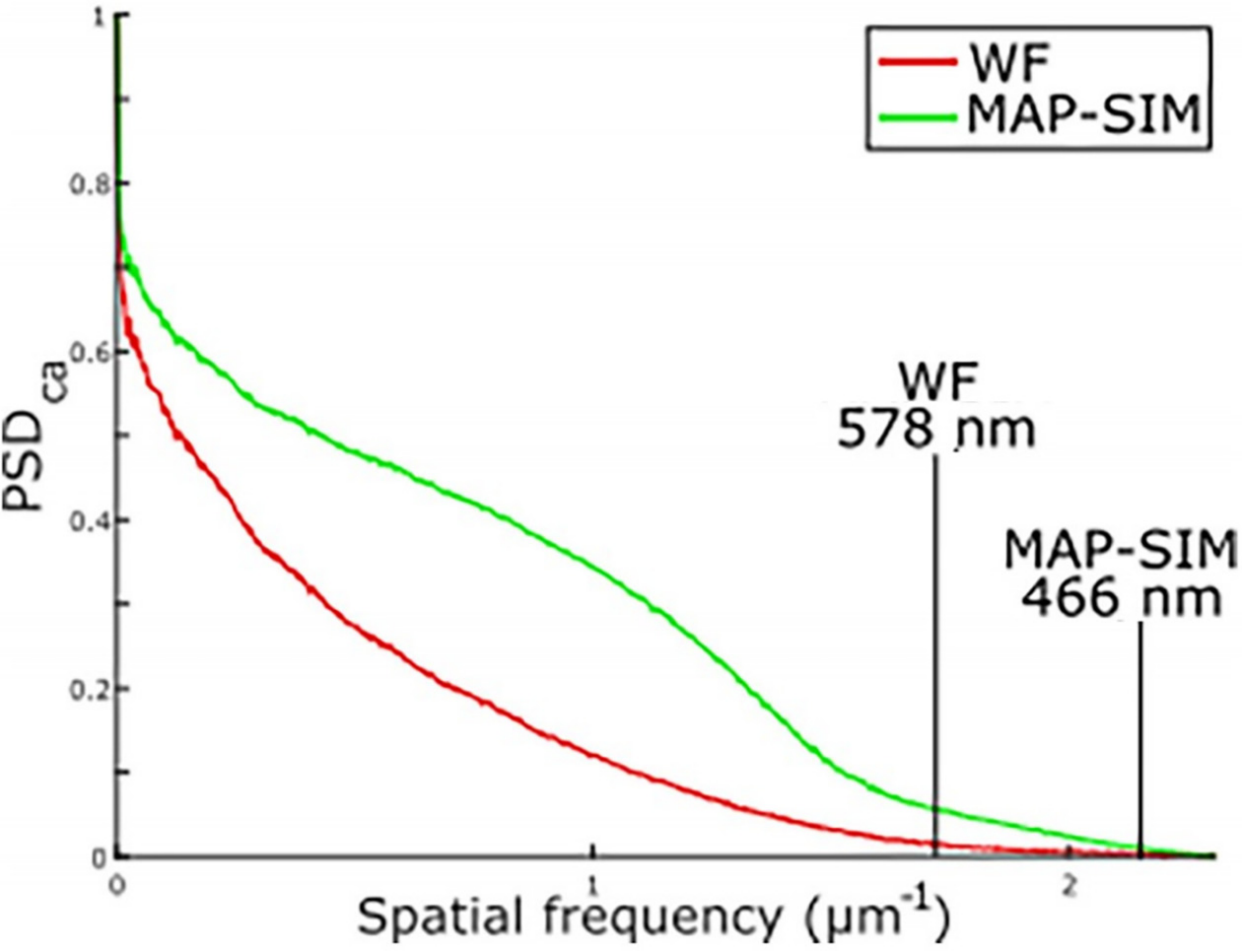
Normalized, radially averaged power spectral density (PSD_ca_) and resolution analysis measured on the tiles shown in Figs 7a and b.

Focusing on the basal cell carcinoma sample, we selected 125 (out of 522 total) image tiles, calculated the PSD and resolution for each tile, and averaged the results. We found that, in the case of this sample, the image resolution was 593 ± 20 nm for WF and 468 ± 2.5 nm for MAP-SIM (average ± standard deviation). These data were acquired with a UPLSAPO 30×/1.05 NA silicone oil immersion objective. Fig. 7 shows an example measurement for 1 image tile. Fig. 8 shows a plot of PSD_ca_ for this image tile.

## Results

**Figure 9:**
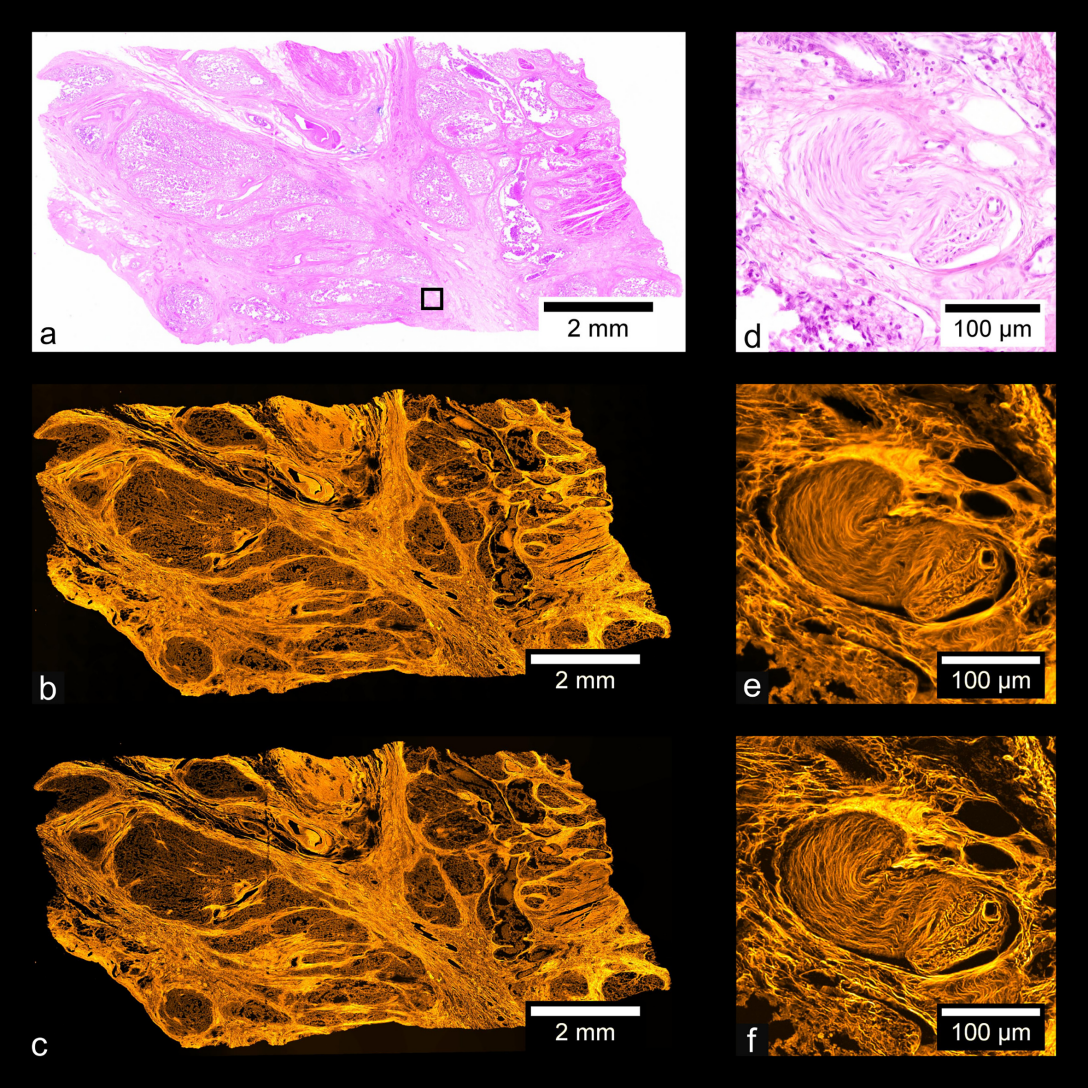
Carcinoma of human prostate. (a) Color overview, (b) WF stitch, (c) MAP-SIM stitch. (d) A region of the sample indicated in (a). (e, f) A zoom-in of (b) and (c), respectively, in the region indicated in (a).

**Figure 10:**
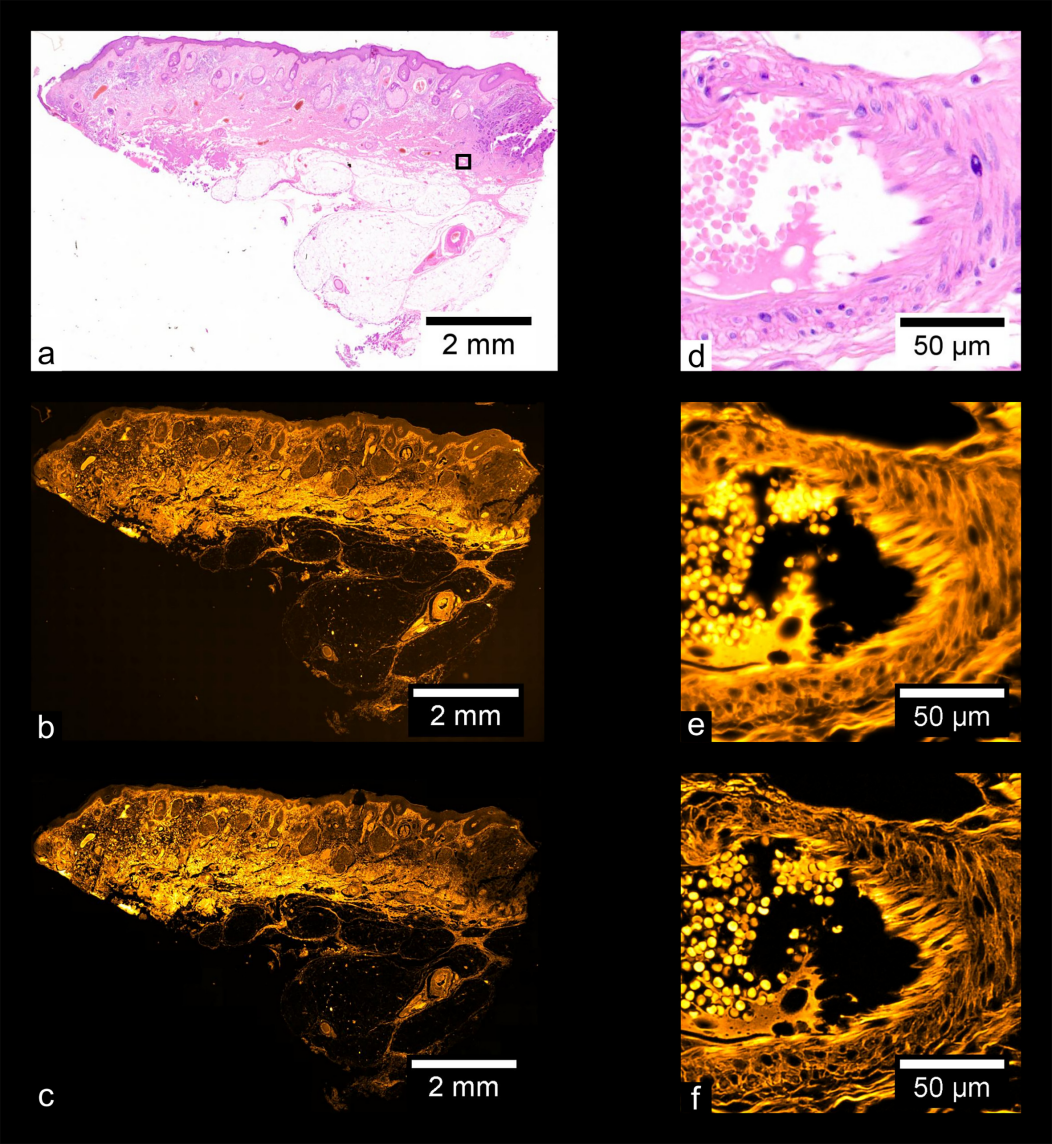
Basal cell carcinoma. (a) Color overview, (b) WF stitch, (c) MAP-SIM stitch. (d) A region of the sample indicated in (a). (e, f) A zoom-in of (b) and (c), respectively, in the region indicated in (a).

**Figure 11:**
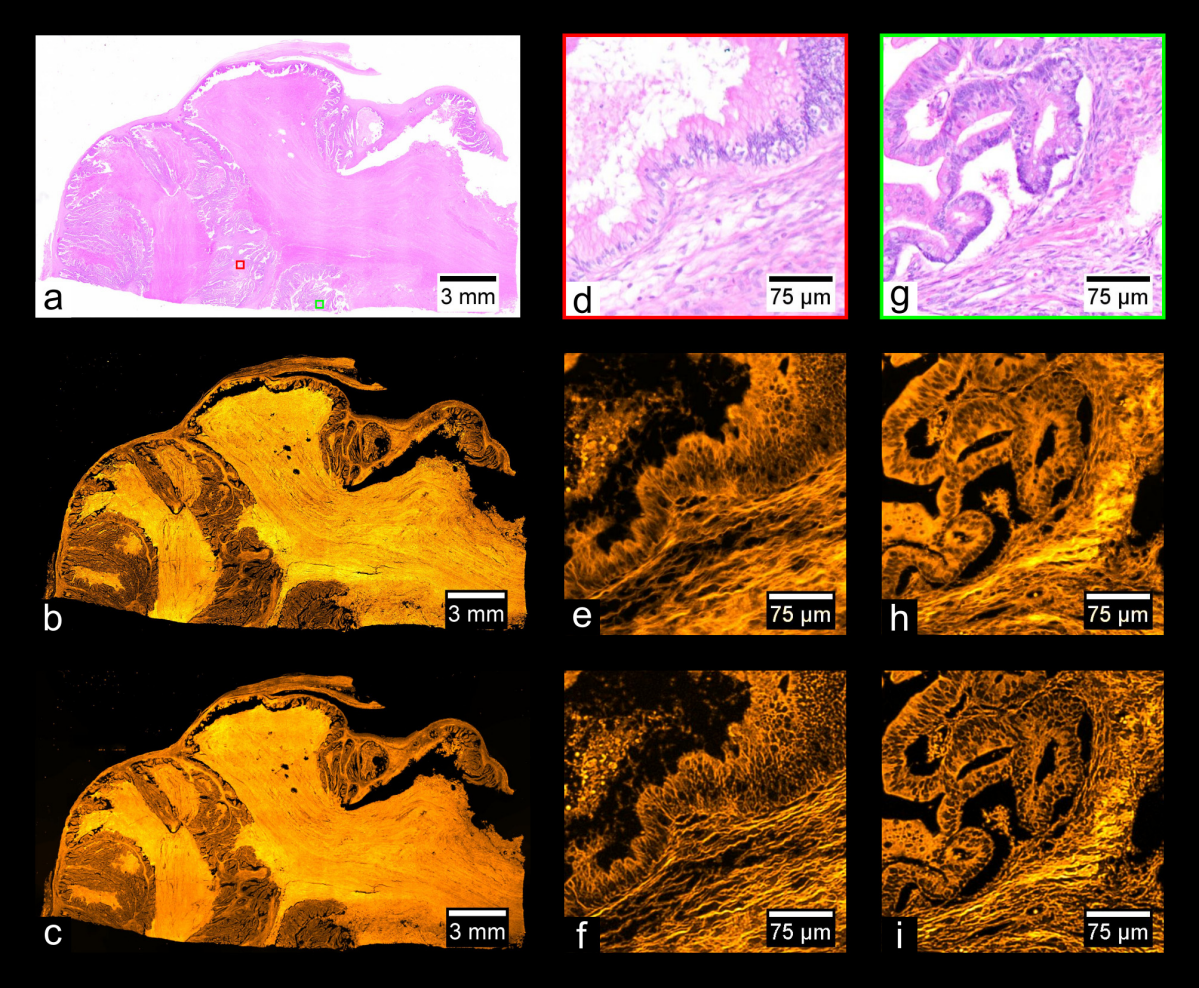
Adenocarcinoma of human ovary. (a) Color overview, (b) WF stitch, (c) MAP-SIM stitch. (d, g) A region of the sample indicated in (a), acquired separately from (a) using a 10× objective. (e, h) A zoom-in of (b), while (f) and (i) show a zoom-in of (c), all in the regions indicated in (a).

**Figure 12:**
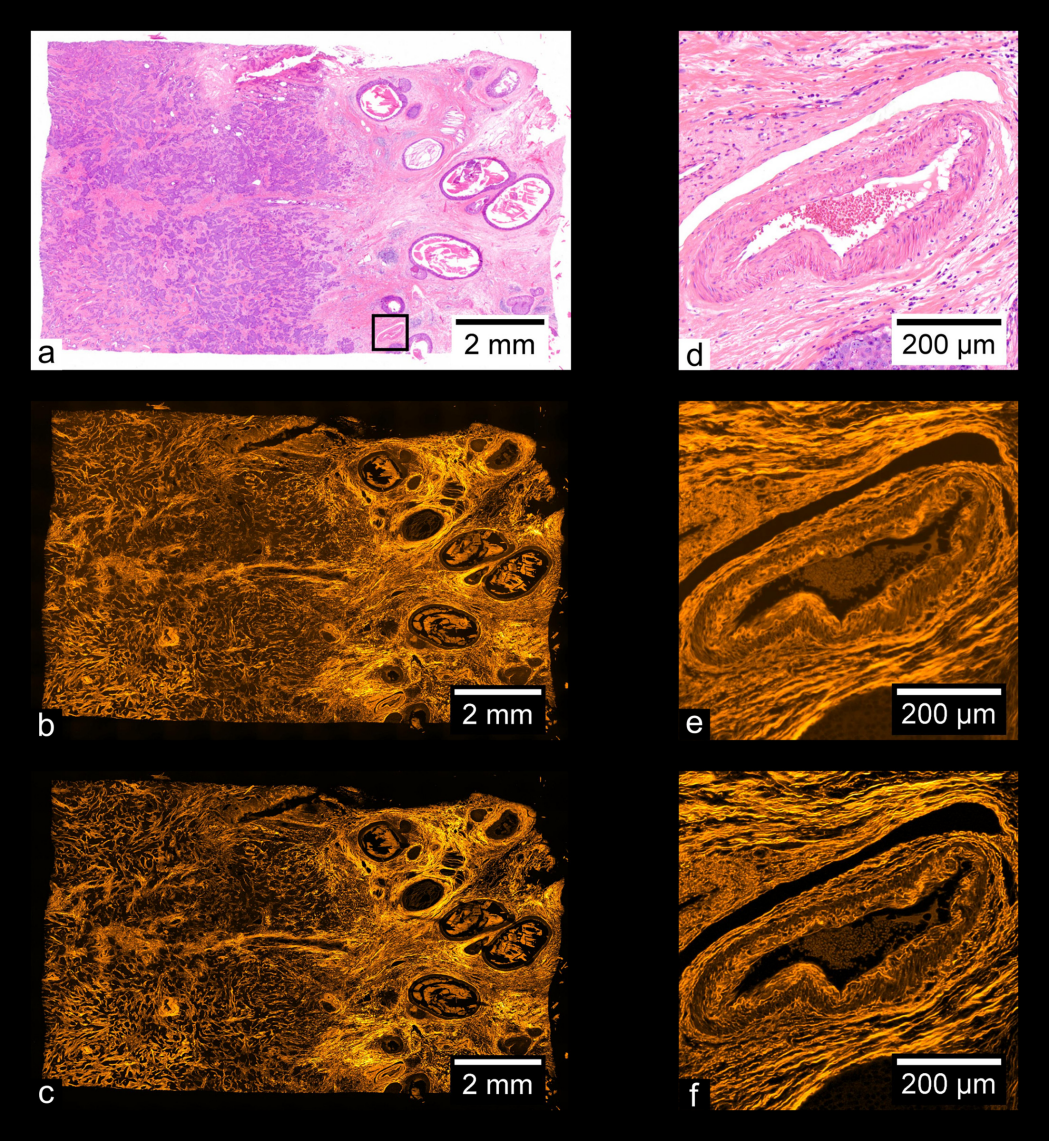
Adenocarcinoma of human breast. (a) Color overview, (b) WF stitch, (c) MAP-SIM stitch. (d) A region of the sample indicated in (a), acquired separately from (a) using a 10× objective. (e, f) A zoom-in of (b) and (c), respectively, in the region indicated in (a).

**Figure 13:**
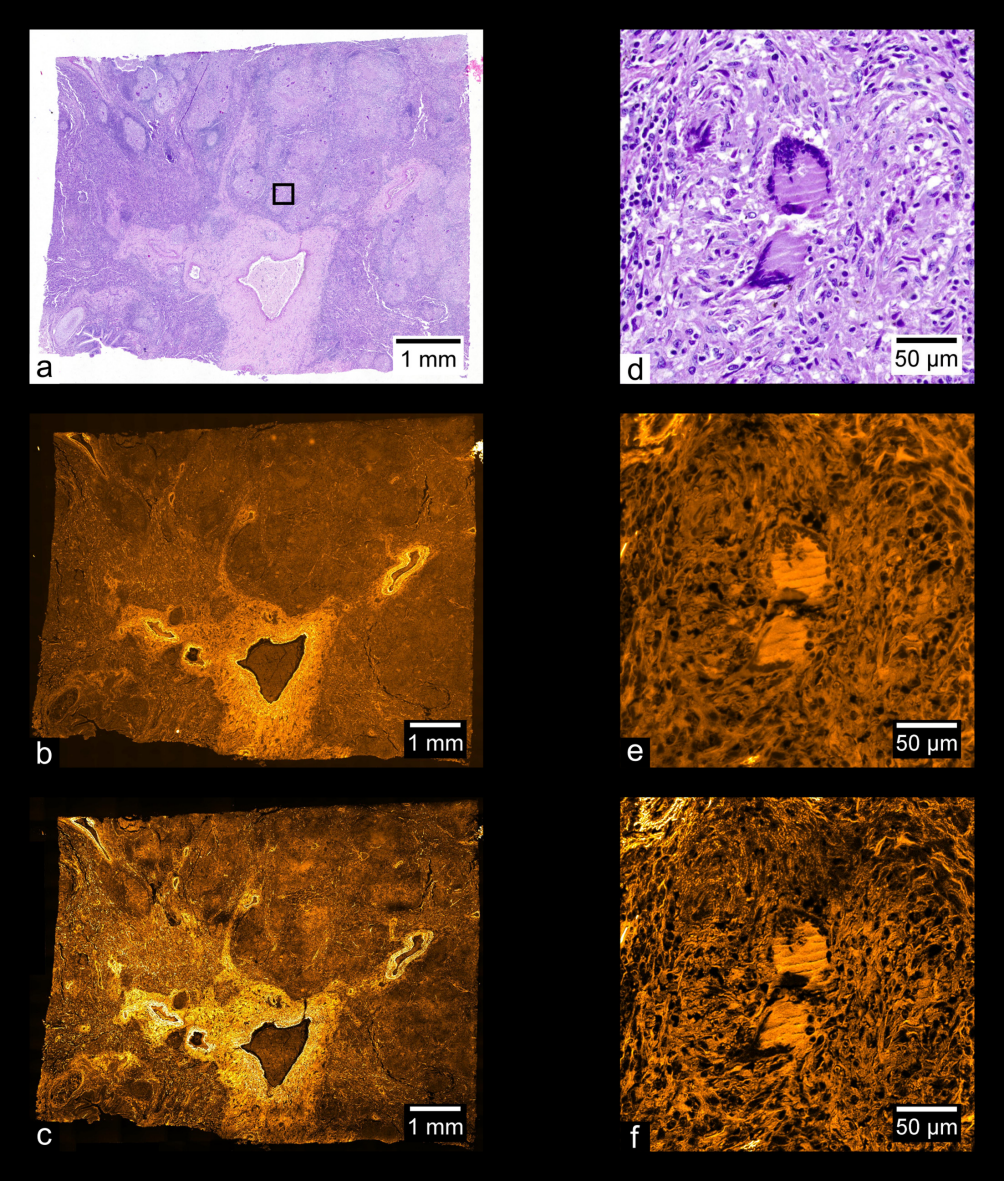
Tuberculosis of human lung. (a) Color overview, (b) WF stitch, (c) MAP-SIM stitch. (d) A region of the sample indicated in (a), acquired separately from (a) using a 20× objective. (e, f) A zoom-in of (b) and (c), respectively, in the region indicated in (a).

Fig. 9 shows images of a prepared slide containing a human prostate carcinoma sample stained with H&E. Fig. 9a shows a stitched color overview, and Fig. 9d shows a zoom-in of the region indicated in Fig. 9a, acquired separately using a UPLSAPO 20×/0.75 NA objective. Fig. 9b shows a stitched wide-field fluorescence image, and Fig. 9c shows a stitched SIM image. Figs 9e and f each show zoom-ins of the stitches shown in Figs 9b and c, respectively. Using the acquisition and processing methods described, whole-slide images are produced without any visible stitching artifacts. Additionally, the MAP-SIM reconstruction method produces resolution superior to that of the wide-field data. Figs. 10–13 show similar comparisons for basal cell carcinoma, ovary adenocarcinoma, breast adenocarcinoma, and tuberculosis of the lung, respectively.

The data shown in Fig. 9–13 are freely available through Giga DB [52]. This dataset includes all color overviews as well as WF and MAP-SIM stitches at full resolution. In addition, all image tiles used to create the WF and MAP-SIM stitches of the samples are provided.

## Discussion

Many past studies into stitching of SIM mosaics have been hindered by noticeable image artifacts, arising from flaws in the optical set-ups used as well as imperfections in the SIM reconstruction and image-stitching processes. While these artifacts are sometimes minimal enough to remain uncorrected, certain artifacts seriously inhibit the usefulness of the final stitched image. Schlichenmeyer et al. [23] note that issues in triggering and evenly illuminating the microdisplay being used for illumination resulted in striping and vignetting artifacts; similarly, in [22, 24, 36, 53], stitching artifacts are apparent in the images.

Here, optimization of the optical set-up, camera-microdisplay synchronization, and image-processing methods yielded whole-slide images free from visible SIM or image-stitching artifacts. In addition to the elimination of artifacts, our use of SIMToolbox to perform SIM reconstruction on the data allows for a variety of reconstruction algorithms to be used, including super-resolution algorithms such as MAP-SIM. This too presents an improvement over previous works.

Another advantage of the acquisition and processing methods demonstrated here is the minimization of user intervention and, in turn, reductions in acquisition and processing time. Stage movement, sample focusing, image acquisition, and SIM pattern advancement are controlled automatically. Loading of the sample, definition of the mosaic edges, and manual focus on 3–5 positions in the sample are the only steps that the user must take before acquisition can begin. Recent developments in autofocus technology for SIM may allow for the manual focus step to be shortened or omitted [53]. These automated steps during acquisition allow for large mosaics to be acquired. The quality of the final stitched images does not degrade for larger mosaics—in fact, the quality of the devignetting process improves with larger datasets because more data are available to produce an accurate estimation of the illumination profile. SIMToolbox (version 2.0), which is capable of utilizing the processing power of modern consumer graphics cards during MAP-SIM processing, also reduces the time spent during the data-processing phase. Finally, unlike other super-resolution reconstruction methods such as SR-SIM, MAP-SIM is able to produce artifact-free results without tuning of reconstruction parameters by the user, a process that is difficult to automate and requires substantial user experience.

One drawback of the method presented here is the inability to image the entire volume of samples thicker than ∼0.5 mm. However, this limitation does not prevent large, unsectioned samples from being imaged, as is the case with bright-field microscopy, where samples must be thin enough for transmitted light to reach the objective. Rather, because the light that illuminates the sample in fluorescence microscopy emanates from the objective, all surface regions of a large sample can be imaged. Additionally, due to the optical sectioning exhibited by SIM, light from out-of-focus regions of the sample is almost completely attenuated. Consequently, imaging the surfaces of large samples with SIM produces high-quality images without the need for physical sectioning, as previously demonstrated [23, 36].

Here, we demonstrated our imaging techniques on traditionally prepared histopathological samples in order to provide a comparison between bright-field imaging and SIM, but the same techniques can be used to image a wide variety of fluorescently labeled samples. The ability to seamlessly image the entire surface region of large samples has multiple potential applications in histopathology. SIM presents unique advantages in analyzing the surgical margins of large tissue excisions, as demonstrated by Wang et al. [36]. Confocal imaging of core needle biopsy samples has been previously demonstrated to produce images suitable for medical diagnosis [42], a practice easily adapted to SIM. The speed at which sample preparation and image acquisition can be performed in fluorescence microscopy presents opportunities for intra-operative analysis of tissue samples using SIM techniques, as mentioned by multiple other studies [23, 36, 54, 55].

### Reuse potential and example of data reuse

The data provided here present various opportunities for reuse. With the multiple high-resolution color overviews and stitched SIM images, comparison of structures visible in the bright-field and fluorescent images could be performed to further study the use of fluorescence microscopy in histopathology. The unstitched image tiles of the basal cell carcinoma sample provided in the dataset, which still contain vignetting artifacts, may be used to reproduce the results of our devignetting process, as well as to further develop new devignetting approaches suited for SIM. These tiles might also be used to create or modify existing stitching software for global minimization of stitching artifacts. Note that the image tiles from the other samples in the dataset are provided after devignetting.

**Figure 14:**
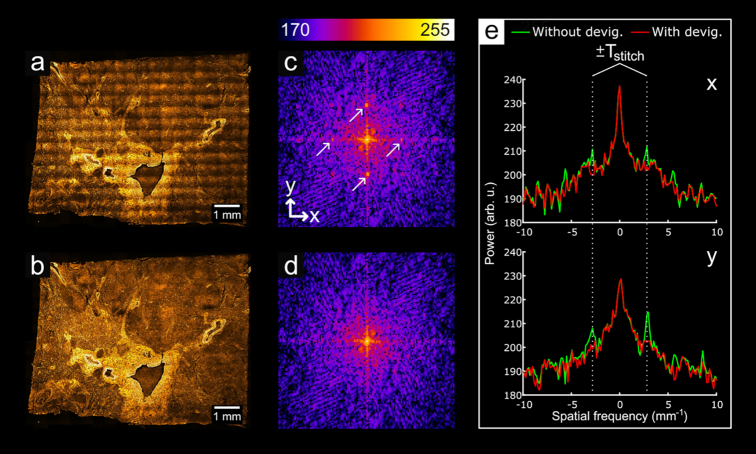
Analysis of periodic stitching artifacts in the frequency domain. (a) A stitched image where devignetting has not been applied to the tiles; (b), a stitch of devignetted data. (c, d) Central region of the FFTs of (a) and (c), respectively. The arrows in (c) point to the fundamental peaks of the grid artifact. (e) Shows plots of (c) and (d) through the center of each image along the x and y axes. The calculated value for *T*_stitch_ aligns well with the peaks along both axes. Arb. u.: arbitrary units; devig.: devignetting.

Gone uncorrected, vignetting in the image tiles used to stitch a larger image can cause a noticeable grid pattern in the final stitched image. While readily noticeable upon viewing of the image, quantification of this pattern is useful for evaluation of methods that remove it. Because this stitching artifact arises from an illumination profile common to each tile, the period of this pattern in the stitched image can simply be represented by the spacing between tiles used during acquisition:

$$\begin{equation*}
{T_{\mathrm{stitch}}} = l \cdot \left( {1 - r} \right),
\end{equation*}$$

where *l* is the image width and *r* is the proportional overlap between image tiles. The parameters for the dataset visualized in Fig. 14 are *l* = 2,048 pixels and *r* = 0.2; thus *T*_stitch_ = 1,638 pixels, or ∼355 µm (because the dataset has a pixel size of ∼216.7 nm). In our set-up, the camera sensor is square, so *T*_stitch_ is the same both horizontally and vertically in the final image. As a pattern with a very consistent period, this grid artifact manifests in the fast Fourier transform (FFT) of an uncorrected stitch as a series of bright peaks. As shown in Fig. 14e, the location of the peaks corresponding to the fundamental frequency of the grid pattern agrees very well with the calculated *T*_stitch_. Figs 14d and e show that the FFT of a properly corrected image contains no trace of the peaks evident in the uncorrected image.

## Availability of Source Code and Requirements

Project name: SIMToolbox version 2.12

Project home page: http://mmtg.fel.cvut.cz/SIMToolbox/

Operating system: platform independent

Programming language: MATLAB

License: GNU General Public License v3.0

The SIMToolbox GUI was compiled with MATLAB 2015a and tested in Windows 7 and 8. The GUI is a stand-alone program and does not require MATLAB to be installed. To use the MATLAB functions within SIMToolbox (i.e., without the GUI), MATLAB must be installed. The functions were mainly developed with 64-bit MATLAB versions 2012b, 2014a, 2015a in Windows 7. When using SIMToolbox functions without the GUI, the MATLAB “Image Processing Toolbox” is required. SIMToolbox also requires the “MATLAB YAML” package to convert MATLAB objects to/from YAML file format. Note that this package is installed automatically when using the GUI.

## Availability of Supporting Data and Materials

All raw and analyzed data are available on GigaDB [52]. All files and data are distributed under the Creative Commons CC0 waiver, with a request for attribution. The data are organized into 5 main folders for the 5 different samples (see Table 3).

**Table 3:** Description of the data files

Folder	Files	File Size
01-Prostate-carcinoma	Prostate-carcinoma-WF-tiles.zip	3.6 GB
	Prostate-carcinoma-MAPSIM-tiles.zip	3.5 GB
	Prostate-carcinoma-WF-stitch.tif	695 Mb
	Prostate-carcinoma-MAPSIM-stitch.tif	695 Mb
	Prostate-carcinoma-Color-stitch.tif	118 MB
02-Basal-cell-carcinoma	Basal-cell-carcinoma-MAPSIM-tiles.zip	7.5 GB
	Basal-cell-carcinoma-WF-tiles.zip	7.5 GB
	Basal-cell-carcinoma-MAPSIM-stitch.tif	6.3 GB
	Basal-cell-carcinoma-WF-stitch.tif	5.7 GB
	Basal-cell-carcinoma-color-stitch.tif	123 MB
03-Ovary-adenocarcinoma	Ovary-adenocarcinoma-MAPSIM-tiles.zip	891 MB
	Ovary-adenocarcinoma-WF-tiles.zip	849 MB
	Ovary-adenocarcinoma-MAPSIM-stitch.tif	916 MB
	Ovary-adenocarcinoma-WF-stitch.tif	979 MB
	Ovary-adenocarcinoma-Color-stitch.tif	610 MB
04-Breast-adenocarcinoma	Breast-adenocarcinoma-MAPSIM-tiles.zip	1.4 GB
	Breast-adenocarcinoma-WF-tiles.zip	1.4 GB
	Breast-adenocarcinoma-MAPSIM-stitch.tif	1.1 GB
	Breast-adenocarcinoma-WF-stitch.tif	1.1 GB
	Breast-adenocarcinoma-Color-stitch.tif	129 MB
05-Lung-tuberculosis	Lung-tuberculosis-MAPSIM-tiles.zip	4.8 GB
	Lung-tuberculosis-WF-tiles.zip	4.6 GB
	Lung-tuberculosis-MAPSIM-stitch.tif	3.8 GB
	Lung-tuberculosis-WF-stitch.tif	3.6 GB
	Lung-tuberculosis-Color-stitch.tif	341 MB

## Abbreviations

Av Int Proj: average intensity projection; FFT: fast Fourier transform; FOV: field of view; GUI: graphical user interface; H&E: hematoxylin-eosin; ICE: Image Composite Editor; MAP-SIM: maximum a posteriori probability SIM; NA: numerical aperture; LCOS: liquid crystal on silicon; PSDca: circularly averaged power spectral density; SIM: structured illumination microscopy; WF: wide field.

## Ethics Approval and Consent to Participate

Because the samples were acquired commercially and because they are completely de-identified (meaning that there is no way to connect these particular samples to the original donor), this is not considered human subject research, and approval is not required to work with these samples.

## Competing Interests

The authors declare that they have no competing interests.

## Funding

Research reported in this publication was supported by the National Institute of General Medical Sciences of the National Institutes of Health under award No. 1R15GM128166–01. This work was also supported by the UCCS BioFrontiers Center. The funding sources had no involvement in study design; in the collection, analysis, and interpretation of data; in the writing of the report; or in the decision to submit the article for publication. This material is based in part upon work supported by the National Science Foundation under Grant No. 1,727,033. Any opinions, findings, and conclusions or recommendations expressed in this material are those of the authors and do not necessarily reflect the views of the National Science Foundation.

## Author Contributions

K.J. acquired data, analyzed data, and wrote the manuscript. G.H. conceived the project, acquired data, analyzed data, supervised research, and wrote the manuscript.

## Supplementary Material

giaa035_Authors_Response_To_Reviewer_Comments

giaa035_GIGA-D-19-00306_Original_Submission

giaa035_GIGA-D-19-00306_Revision_1

giaa035_GIGA-D-19-00306_Revision_2

giaa035_Response_to_Reviewer_Comments_Original_Submission

giaa035_Reviewer_1_Report_Original_Submission Chris Armit -- 9/18/2019 Reviewed

giaa035_Reviewer_2_Report_Original_Submission Marcus Wagner -- 10/18/2019 Reviewed

giaa035_Reviewer_2_Report_Revision_1 Marcus Wagner -- 1/24/2020 Reviewed
