# Supplementary material for: Artifact-free whole-slide imaging with structured illumination microscopy and Bayesian image reconstruction
Source: Gigascience. 2020 Apr 14;9(4):giaa035. doi: 10.1093/gigascience/giaa035 (PMC7155289; doi:10.1093/gigascience/giaa035)
Supplement: giaa035_GIGA-D-19-00306_Original_Submission [file giaa035_giga-d-19-00306_original_submission.pdf]

GigaScience

Artifact-free whole-slide imaging with structured illumination microscopy and Bayesian image reconstruction

--Manuscript Draft--

**Manuscript Number:**	GIGA-D-19-00306
**Full Title:**	Artifact-free whole-slide imaging with structured illumination microscopy and Bayesian image reconstruction
**Article Type:**	Data Note
**Funding Information:**	National Institute of General Medical Sciences (1R15GM128166-01) — Dr Guy M Hagen<br>Division of Biological Infrastructure (1727033) — Dr Guy M Hagen
**Abstract:**	Background<br><br>Structured illumination microscopy (SIM) is a method which can be used to image biological samples and can achieve both optical sectioning and super-resolution effects. Optimization of the imaging setup and data processing methods results in high quality images without artifacts due to mosaicking or due to the use of SIM methods. Reconstruction methods based on Bayesian estimation can be used to produce images with a resolution beyond that dictated by the optical system.Findings<br><br>Five complete datasets are presented including large panoramic SIM images of human tissues in pathophysiological conditions. Cancers of the prostate, skin, ovary, and breast, as well as tuberculosis of the lung, were imaged using SIM. The samples are available commercially and are standard histological preparations stained with hematoxylin and eosin.Conclusion<br><br>The use of fluorescence microscopy is increasing in histopathology. There is a need for methods which reduce artifacts when employing image stitching methods or optical sectioning methods such as SIM. Stitched SIM images produce results which may be useful for intraoperative histology. Releasing high quality, full slide images and related data will aid researchers in furthering the field of fluorescent histopathology.
**Corresponding Author:**	Guy M Hagen, PHD<br><br>UNITED STATES
**Corresponding Author Secondary Information:**	
**Corresponding Author's Institution:**	
**Corresponding Author's Secondary Institution:**	
**First Author:**	Karl A Johnson
**First Author Secondary Information:**	
**Order of Authors:**	Karl A Johnson<br>Guy M Hagen, PHD
**Order of Authors Secondary Information:**	
**Additional Information:**	
**Question**	**Response**
Are you submitting this manuscript to a special series or article collection?	No

Powered by Editorial Manager® and ProduXion Manager® from Aries Systems Corporation

**Experimental design and statistics**<br><br>Full details of the experimental design and statistical methods used should be given in the Methods section, as detailed in our Minimum Standards Reporting Checklist. Information essential to interpreting the data presented should be made available in the figure legends.<br><br>Have you included all the information requested in your manuscript?	Yes
**Resources**<br><br>A description of all resources used, including antibodies, cell lines, animals and software tools, with enough information to allow them to be uniquely identified, should be included in the Methods section. Authors are strongly encouraged to cite Research Resource Identifiers (RRIDs) for antibodies, model organisms and tools, where possible.<br><br>Have you included the information requested as detailed in our Minimum Standards Reporting Checklist?	Yes
**Availability of data and materials**<br><br>All datasets and code on which the conclusions of the paper rely must be either included in your submission or deposited in publicly available repositories (where available and ethically appropriate), referencing such data using a unique identifier in the references and in the "Availability of Data and Materials" section of your manuscript.<br><br>Have you have met the above requirement as detailed in our Minimum Standards Reporting Checklist?	Yes

manuscript Click here to download Manuscript Manuscript.docx

Artifact-free whole-slide imaging with structured illumination microscopy and Bayesian image reconstruction

Karl Johnson[1], Guy M. Hagen[1*]

[1]UCCS center for the Biofrontiers Institute, University of Colorado at Colorado Springs

1420 Austin Bluffs Parkway, Colorado Springs, Colorado, 80918, USA

*ghagen@uccs.edu

Abstract

Background Structured illumination microscopy (SIM) is a method which can be used to image biological samples and can achieve both optical sectioning and super-resolution effects. Optimization of the imaging setup and data processing methods results in high quality images without artifacts due to mosaicking or due to the use of SIM methods. Reconstruction methods based on Bayesian estimation can be used to produce images with a resolution beyond that dictated by the optical system.

Findings Five complete datasets are presented including large panoramic SIM images of human tissues in pathophysiological conditions. Cancers of the prostate, skin, ovary, and breast, as well as tuberculosis of the lung, were imaged using SIM. The samples are available commercially and are standard histological preparations stained with hematoxylin and eosin.

Conclusion The use of fluorescence microscopy is increasing in histopathology. There is a need for methods which reduce artifacts when employing image stitching methods or optical sectioning methods such as SIM. Stitched SIM images produce results which may be useful for intraoperative histology. Releasing high quality, full slide images and related data will aid researchers in furthering the field of fluorescent histopathology.

Keywords Structured illumination microscopy, SIM, image stitching, Bayesian methods, MAP-SIM, SIMToolbox, histopathology, cancer.

Data description

Context

Structured illumination microscopy (SIM) is a method in optical fluorescence microscopy which can achieve both optical sectioning (OS-SIM) [1] and resolution beyond the diffraction limit (SR-SIM) [2,3]. SIM has been used for super-resolution imaging of both fixed and live cells [4–7] and has matured enough as a method that it is now available commercially. In SIM, a set of images is acquired using an illumination pattern which shifts between each image. As SIM has developed, diverse strategies have been proposed for creation of the SIM pattern [1,8–13]. Several different approaches for processing the data have also been introduced [3,7,8,14–16].

Recently, microscope systems capable of imaging with high resolution and a large field of view (FOV) have been developed [17–21], some using custom-made microscope objectives. However, stitching together images acquired with a higher magnification objective to create a large mosaic remains a valid and popular approach. Some published results involving stitched images suffer from pronounced artifacts in which the edges of the individual sub-images are visible, usually as dark bands which outline each sub-image [22–24]. On the other hand, several studies have proposed methods for stitching of microscope images with reduced artifacts [25–32].

The combination of SIM with image stitching methods allows collection of large FOV images with both optical sectioning and super-resolution properties. Here, we demonstrate methods and provide complete datasets for five different samples. The samples are hematoxylin and eosin (H&E) stained histological specimens which provide examples of human diseases (ovarian cancer, breast cancer, prostate cancer, skin cancer, and tuberculosis), and which are also available commercially for those who wish to reproduce our work. We used freely available optical designs [6,10,33] and open source software [33] for SIM imaging, along with freely available software for image stitching (Microsoft Image Composite Editor (ICE) [34], or a well validated plugin [26] for ImageJ [35]). Combining this with devignetting methods, we produced stitched images which are free of noticeable artifacts from stitching or from SIM reconstruction.

Fluorescence microscopy is becoming more important in histopathology. Traditional bright field microscopy diagnostic methods require a time-consuming process, involving chemical fixation and physical sectioning. The use of optical sectioning fluorescence microscopy allows high-quality images to be captured without the need for physical sectioning. Consequently, it has been shown that imaging can be performed on large human tissue samples within 1 hour after excision [36]. Additionally, other studies have shown the results of fluorescence

imaging to be usable and accurate in diagnosis of various medical conditions [37–42]. Previously, it was noted that obvious stitching artifacts significantly decrease the usability of large fluorescence images in medical diagnosis. In one case, such artifacts resulted in the rejection of over half of the images acquired [38]. The setup we describe here allows for fast, artifact-free, high-resolution imaging of fluorescent samples, and is compatible with samples stained with most fluorescent dyes.

Methods

Samples

All samples used in this study are available from Carolina Biological, Omano, or Ward's Science. The samples are approximately 7 μm thick and are stained with hematoxylin and eosin. The commercial source, product number, and other SIM imaging parameters for each sample are detailed in Table 1. Table 2 details imaging parameters for acquisitions of each sample with a color camera.

Table 1: Imaging parameters for the SIM datasets

Sample	Source company and product no.	SIM pattern no. of phases	Exposure time, ms	No. of tiles	Objective mag/NA	Acquisition time, s	Stitching software
Carcinoma of Prostate	Carolina, 318492	5	50	23 × 11	20×/0.45	315	Microsoft ICE
Basal Cell Carcinoma	Ward's Science, 470183-256	6	75	29 × 18	30×/1.05	821	FIJI
Adenocarcinoma of Ovary	Carolina, 318628	5	100	25 × 14	10×/0.4	595	Microsoft ICE
Adenocarcinoma of Breast	Carolina, 318766	8	200	12 × 8	10×/0.4	278	FIJI
Lung Tuberculosis	Omano, OMSK-HP50	5	100	20 × 16	30×/1.05	541	FIJI

Table 2: Parameters for the color images

Sample	No. of tiles	Objective mag/NA
Carcinoma of Prostate	6 × 5	4×/0.16
Basal Cell Carcinoma	5× 5	4×/0.16

Adenocarcinoma of Ovary	11 × 11	4×/0.16
Adenocarcinoma of Breast	6 × 6	4×/0.16
Lung Tuberculosis	8 × 10	10×/0.4

Microscope setup and data acquisition

We used a home-built SIM setup based on the same design as described previously [6,10,15] (Fig. 1). The SIM system is based on an IX83 microscope (Olympus) equipped with a Zyla 4.2+ sCMOS camera (Andor) under the control of IQ3 software (Andor). We used the following Olympus objectives: UPLSAPO 4×/0.16 NA, UPLSAPO 10×/0.4 NA, LUCPLFLN 20×/0.45 NA, and UPLSAPO 30×/1.05 NA silicone oil immersion. For color images we used an aca1920-40uc color camera (Basler) under control of Pylon software (Basler). We used a MS-2000 motorized microscope stage (Applied Scientific Instrumentation) to acquire tiled SIM images. In all datasets, the stage scanning was configured such that all image edges overlapped by 20%.

Briefly, the SIM system uses a ferroelectric liquid crystal on silicon (LCOS) microdisplay (type SXGA-3DM, Forth Dimension Displays). This device has been used previously in SIM and related methods in fluorescence microscopy [5,10,15,33,43–47] and allows one to produce patterns of illumination on the sample which can be reconfigured at will by changing the image displayed on the device. The light source (Lumencor Spectra-X) is toggled off between SIM patterns and during camera readout. Close synchronization between the camera acquisitions, light source, and microdisplay ensures rapid image acquisition, helps reduce artifacts, and reduces light exposure to the sample. The supplementary material contains more information about system integration.

INSERT FIGURE 1

SIM data processing

SIM reconstructions were performed using SIMToolbox, an open-source and freely available program that our group developed for processing SIM data [33]. We generated optically sectioned, enhanced resolution images using a Bayesian estimation method, maximum *a posteriori* probability SIM (MAP-SIM) [15]. MAP-SIM works using maximum *a posteriori* probability methods, which are well known in microscopy applications [48,49], to enhance high spatial frequency image information. We then combine this information, in the frequency domain, with low spatial frequency image information obtained by OS-SIM methods, then produce the final image by an

inverse Fourier transform [15]. We typically measure the final resolution obtained by analyzing the frequency spectrum of the resulting image, as is discussed below.

The illumination patterns used here are generated such that the sum of all positions in each pattern set results in homogenous illumination. As such, a widefield (WF) image can be reconstructed from SIM data simply by performing an average intensity projection of the patterned images. This can be described by

$$I_{WF} = \frac{1}{N}\sum_{n=1}^{N} I_n,$$

where N is the number of pattern phases, I_n is the image acquired on the nth illumination position, and I_{WF} is the WF reconstruction. This is the method we used to generate WF images throughout this study.

Vignetting correction

Following SIM reconstruction, vignetting artifacts remain in each tile. If not removed prior to stitching, this vignetting introduces a distracting grid pattern in the final stitched image. We performed vignette removal by dividing each tile of the mosaic by an image representing the vignetting profile common to all tiles. Other studies have used an image of a uniformly fluorescent calibration slide as a reference for vignette removal [36], where information concerning non-uniform illumination is captured. However, we found that SIM processing introduces vignetting artifacts beyond those due to non-uniform illumination. Additionally, these artifacts vary depending on properties of the sample being imaged. As such, performing pre-acquisition calibration on a uniformly fluorescent slide is not sufficient to remove vignetting artifacts from SIM reconstructions. Instead, an estimate of the vignetting profile is found through analysis of the mosaic tiles after SIM reconstruction.

A blurred average intensity projection of the tiles is a good approximation of the vignetting profile, as an average intensity projection merges the tiles into a single image with reduced foreground information while preserving vignetting. Subsequent blurring with an appropriate radius and edge-handling method also eliminates the high spatial frequency foreground without impacting the low spatial frequency illumination profile. To eliminate errors during the blurring step due to the blurring area extending outside the original image, we used an edge handling method in which the blurring area is reduced near the edges of the image such that no values outside the image border are sampled. Unlike edge handling methods in which the image is padded with a uniform value (or

mirrored and tiled) to accommodate a blurring area which extends beyond the original image limits, this method is free from major artifacts, such as erroneous brightness of the image edges (see supplementary figure S1). This approximation of the illumination profile works especially well for histological samples, as such samples are non-sparse and require many tiles, factors which improve the accuracy this approach. We performed all steps of this devignetting process using built-in functions and the 'Fast Filters' plugin in ImageJ [50]. The effect of devignetting is illustrated in Fig. 2.

INSERT FIGURE 2

Image Stitching

With visible vignetting removed, we then stitched together a composite image from the tiles. The pre-processing allows for stitching to be done in various stitching applications; Microsoft ICE and Preibisch's plugin for FIJI [26] were used to stitch the data presented here.

The data processing procedure is summarized in Fig. 3. The total time for processing each dataset was about 30 min.

INSERT FIGURE 3

Color image data processing methods

We created color overview images by stitching devignetted brightfield acquisitions. Devignetting was performed simply by adding the inverse of an empty brightfield acquisition to each color tile using ImageJ. For this method to produce optimal results, the empty brightfield image must be acquired in conditions identical to those of the raw tile data, such that the illumination profile in the empty image matches that of the unprocessed tiles. This simple operation removes nearly all visible vignetting and color balance artifacts within each tile. The results after devignetting were then stitched using Preibisch's plugin for FIJI [26].

Resolution measurement

We evaluated our results by measuring image resolution using SR Measure Toolbox. SR Measure Toolbox [51] measures the resolution limit of input images through analysis of the normalized, radially averaged power spectral density (PSD_{ca}) of the images, as previously described [6]. Briefly, the resolution limit in real space

is determined by evaluating the cutoff frequency in Fourier space. The cutoff frequency is estimated by calculating the spatial frequency at which the PSD_{ca} (after noise correction) drops to zero.

Focusing on the basal cell carcinoma sample, we selected 125 (out of 522 total) image tiles, calculated the PSD and resolution for each tile, and averaged the results. We found that, in the case of this sample, the image resolution was 593 ± 20 nm for WF and 468 ± 2.5 nm for MAP-SIM (average ± standard deviation). This data was acquired with a UPLSAPO 30×/1.05 NA silicone oil immersion objective. Figure 4 shows an example measurement for one image tile. Figure 5 shows a plot of PSD_{ca} for this image tile.

INSERT FIGURE 4

INSERT FIGURE 5

Results

Figure 6 shows images of a prepared slide containing a human prostate carcinoma sample stained with H&E. Fig. 6a shows a stitched color overview, and Fig. 6d shows a zoom-in of the region indicated in Fig. 6a, acquired separately using a UPLSAPO 20×/0.75NA objective. Fig. 6b shows a stitched widefield fluorescence image, and Fig. 6c shows a stitched SIM image. Figs. 6e and 6f each show zoom-ins of the stitches shown in Figs. 6b and 6c, respectively. Using the acquisition and processing methods described, whole-slide images are produced without any visible stitching artifacts. Additionally, the MAP-SIM reconstruction method produces resolution superior to that of the widefield data.

Figures 7-10 show similar comparisons for basal cell carcinoma, ovary adenocarcinoma, breast adenocarcinoma, and tuberculosis of the lung, respectively.

The data shown in figures 6-10 is freely available through Giga DB [reference to be added]. This dataset includes all color overviews as well as WF and MAP-SIM stitches at full resolution. In addition, all image tiles (prior to devignetting) used to create the WF and MAP-SIM stitches of the basal cell carcinoma sample are provided.

INSERT FIGURE 6

INSERT FIGURE 7

INSERT FIGURE 8

INSERT FIGURE 9

INSERT FIGURE 10

Discussion

Many past studies into stitching of SIM mosaics have suffered from noticeable image artifacts, arising from flaws in the optical setups used as well as imperfections in the SIM reconstruction and image stitching processes. While these artifacts are sometimes minimal enough to remain uncorrected, certain artifacts seriously inhibit the usefulness of the final stitched image. In [23], the authors note that issues in triggering and evenly illuminating the microdisplay being used for illumination resulted in striping and vignetting artifacts; similarly, in [22,24,36,52], stitching artifacts are apparent in the images. Here, optimization of the optical setup, camera-microdisplay synchronization, and image processing methods yielded whole-slide images free from visible SIM or image stitching artifacts. In addition to the elimination of artifacts, our use of SIMToolbox to perform SIM reconstruction on the data allows for a variety of reconstruction algorithms to be used, including super-resolution algorithms such as MAP-SIM. This too presents an improvement over previous works. Our methods also allow for stitching of high-magnification tiles into large-FOV images with subdiffractive detail (see supplementary Fig. S3).

Another advantage of the acquisition and processing methods demonstrated here is the minimization of user intervention, and in turn, reductions in acquisition and processing time. Firstly, the use of Andor IQ during acquisition allows for stage movement, sample focusing, image acquisition, and SIM pattern advancement to be controlled automatically. Loading of the sample, definition of the mosaic edges, and manual focus on 3-5 positions of the sample are the only steps needed to be taken by the user before acquisition can begin. Recent developments in autofocus technology for SIM may allow for the manual focus step to be shortened or omitted [52]. These automated steps during acquisition allow for large mosaics to be acquired. The quality of the final stitched images does not degrade for larger mosaics – in fact, the quality of the devignetting process improves with larger datasets, as more data is available to produce an accurate estimation of the illumination profile. SIMToolbox (version 2.0), which is capable of utilizing the processing power of modern consumer graphics cards during MAP-SIM processing, also reduces the time spent during the data processing phase. Finally, unlike other super-resolution reconstruction

methods such as SR-SIM, MAP-SIM is able to produce artifact-free results without tuning of reconstruction parameters by the user, a process which is difficult to automate and requires significant user experience.

One drawback the method presented here is the inability to image the entire volume of samples thicker than ~0.5 mm. However, this limitation does not prevent large, unsectioned samples from being imaged, as is the case with bright field microscopy, where samples must be thin enough for transmitted light to reach the objective. Rather, as the light which illuminates the sample in fluorescence microscopy emanates from the objective, all surface regions of a large sample may be imaged. Additionally, due to the optical sectioning exhibited by SIM, light from out-of-focus regions of the sample is almost completely attenuated. Consequently, imaging the surfaces of large samples with SIM produces high-contrast images of thin regions without the need for physical sectioning, as previously demonstrated [23,36].

Here, we demonstrated our imaging techniques on traditionally prepared histopathological samples in order to provide a comparison between bright field imaging and SIM, but the same techniques can be used to image a wide variety of fluorescently labelled samples, as demonstrated in the supplementary material. The ability to seamlessly image the entire surface region of large samples has multiple potential applications in histopathology. SIM presents unique advantages in analyzing the surgical margins of large tissue excisions, as demonstrated by Wang [36]. Briefly, due to the ability of SIM to image an unsectioned sample, analysis of surgical margins using SIM requires imaging of far less surface area than that needed for bright field imaging. Confocal imaging of core needle biopsy samples has been previously demonstrated to produce images suitable for medical diagnosis [42], a practice easily adapted to SIM. The speed at which sample preparation and image acquisition can be performed in fluorescence microscopy presents opportunities for intra-operative analysis of tissue samples using SIM techniques, as mentioned by multiple other studies [23,36,53,54].

Reuse potential

The data provided here presents various opportunities for reuse. Firstly, the unstitched image tiles provided in the dataset, which still contain vignetting artifacts, may be used to reproduce the results of our devignetting process, as well as to further develop more sophisticated devignetting approaches suited for SIM. These tiles might also be used to create or modify existing stitching software for global minimization of stitching artifacts. For example, the frequency-domain detection of periodic stitching artifacts discussed in the supplementary material

could be used to minimize such artifacts in developing new stitching software. With the multiple high-resolution color overviews and stitched SIM images, comparison of structures visible in the brightfield and fluorescent images could be performed to further study the use of fluorescence microscopy in histopathology.

Availability of source code and requirements

Project name: SIMToolbox version 2.12

Project home page: http://mmtg.fel.cvut.cz/SIMToolbox/

Operating system: platform independent

Programming language: MATLAB

License: GNU General Public License v3.0

Detailed software compatibility notes

The SIMToolbox GUI was compiled with MATLAB 2015a and tested in Windows 7 and 8. The GUI is a stand-alone program and does not require MATLAB to be installed. To use the MATLAB functions within SIMToolbox (i.e., without the GUI), MATLAB must be installed. The functions were mainly developed with 64 bit MATLAB versions 2012b, 2014a, 2015a in Windows 7. When using SIMToolbox functions without the GUI, the MATLAB "Image Processing Toolbox" is required. SIMToolbox also requires the "MATLAB YAML" package to convert MATLAB objects to/from YAML file format. Note that this package is installed automatically when using the GUI.

Availability of data

All raw and analyzed data is available on GigaDB at http://gigadb.org/site/index.

Abbreviations

Av Int Proj, average intensity projection; FOV, field of view; H&E, hematoxylin and eosin; ICE, Image Composite Editor; MAP-SIM, maximum *a posteriori* probability SIM; NA, numerical aperture; LCOS, liquid crystal on silicon; PSDca, circularly averaged power spectral density; SIM, structured illumination microscopy; WF, wide field.

Ethics approval and consent to participate

Not applicable

Consent for publication

Not applicable

Competing interests

The authors declare that they have no competing interests.

Funding

Research reported in this publication was supported by the National Institute of General Medical Sciences of the National Institutes of Health under award number 1R15GM128166-01. This work was also supported by the UCCS center for the University of Colorado BioFrontiers Institute. The funding sources had no involvement in study design; in the collection, analysis and interpretation of data; in the writing of the report; or in the decision to submit the article for publication. This material is based in part upon work supported by the National Science Foundation under Grant Number 1727033. Any opinions, findings, and conclusions or recommendations expressed in this material are those of the authors and do not necessarily reflect the views of the National Science Foundation.

Author Contributions

KJ: acquired data, analyzed data, wrote the paper

GH: conceived project, acquired data, analyzed data, supervised research, wrote the paper

References

1. M. A. A. Neil, R. Juškaitis, and T. Wilson, "Method of obtaining optical sectioning by using structured light in a conventional microscope," Opt. Lett. **22**, 1905–1907 (1997).
2. M. G. L. Gustafsson, "Surpassing the lateral resolution limit by a factor of two using structured illumination microscopy," J. Microsc. **198**, 82–87 (2000).
3. R. Heintzmann and C. Cremer, "Laterally modulated excitation microscopy: improvement of resolution by using a diffraction grating," Proc. SPIE **3568**, 185–196 (1998).
4. L. Schermelleh, P. M. Carlton, S. Haase, L. Shao, L. Winoto, P. Kner, B. Burke, M. C. Cardoso, D. A. Agard, M. G. L. Gustafsson, H. Leonhardt, and J. W. Sedat, "Subdiffraction multicolor imaging of the nuclear periphery with 3D structured illumination microscopy," Science (80-.). **320**, 1332–1336 (2008).
5. R. Fiolka, L. Shao, E. H. Rego, M. W. Davidson, and M. G. L. Gustafsson, "Time-lapse two-color 3D imaging of live cells with doubled resolution using structured illumination," Proc. Natl. Acad. Sci. U. S. A. **109**, 5311–5315 (2012).
6. J. Pospíšil, T. Lukeš, J. Bendesky, K. Fliegel, K. Spendier, and G. M. Hagen, "Imaging tissues and cells beyond the diffraction limit with structured illumination microscopy and Bayesian image reconstruction," Gigascience **8**, giy126 (2019).
7. X. Huang, J. Fan, L. Li, H. Liu, R. Wu, Y. Wu, L. Wei, H. Mao, A. Lal, P. Xi, L. Tang, Y. Zhang, Y. Liu, S.

Tan, and L. Chen, "Fast, long-term, super-resolution imaging with Hessian structured illumination microscopy," Nat. Biotechnol. (2018).

8. M. G. L. Gustafsson, L. Shao, P. M. Carlton, C. J. R. Wang, I. N. Golubovskaya, W. Z. Cande, D. A. Agard, and J. W. Sedat, "Three-dimensional resolution doubling in widefield fluorescence microscopy by structured illumination," Biophys. J. **94**, 4957–4970 (2008).

9. S. Rossberger, G. Best, D. Baddeley, R. Heintzmann, U. Birk, S. Dithmar, and C. Cremer, "Combination of structured illumination and single molecule localization microscopy in one setup," J. Opt. **15**, 094003 (2013).

10. P. Křížek, I. Raška, and G. M. Hagen, "Flexible structured illumination microscope with a programmable illumination array," Opt. Express **20**, 24585 (2012).

11. P. Kner, B. B. Chhun, E. R. Griffis, L. Winoto, and M. G. L. Gustafsson, "Super-resolution video microscopy of live cells by structured illumination," Nat. Methods **6**, 339–342 (2009).

12. L. J. Young, F. Ströhl, and C. F. Kaminski, "A Guide to Structured Illumination TIRF Microscopy at High Speed with Multiple Colors," J. Vis. Exp. e53988–e53988 (2016).

13. V. Poher, H. X. Zhang, G. T. Kennedy, C. Griffin, S. Oddos, E. Gu, D. S. Elson, M. Girkin, P. M. W. French, M. D. Dawson, and M. A. Neil, "Optical sectioning microscope with no moving parts using a micro-stripe array light emitting diode," Opt. Express **15**, 11196–11206 (2007).

14. F. Orieux, E. Sepulveda, V. Loriette, B. Dubertret, and J.-C. Olivo-Marin, "Bayesian estimation for optimized structured illumination microscopy.," IEEE Trans. Image Process. **21**, 601–14 (2012).

15. T. Lukeš, P. Křížek, Z. Švindrych, J. Benda, M. Ovesný, K. Fliegel, M. Klíma, and G. M. Hagen, "Three-dimensional super-resolution structured illumination microscopy with maximum a posteriori probability image estimation," Opt. Express **22**, 29805–17 (2014).

16. T. Lukeš, G. M. Hagen, P. Křížek, Z. Švindrych, M. Klíma, and K. Fliegel, "Comparison of image reconstruction methods for structured illumination microscopy," Proc. SPIE **9129**, 91293J (2014).

17. C. A. Werley, M.-P. Chien, and A. E. Cohen, "An ultrawidefield microscope for high-speed fluorescence imaging and targeted optogenetic stimulation," Biomed. Opt. Express **8**, 5794 (2017).

18. N. J. Sofroniew, D. Flickinger, J. King, and K. Svoboda, "A large field of view two-photon mesoscope with subcellular resolution for in vivo imaging," Elife **5**, (2016).

19. G. McConnell, J. Trägårdh, R. Amor, J. Dempster, E. Reid, and W. B. Amos, "A novel optical microscope for imaging large embryos and tissue volumes with sub-cellular resolution throughout," Elife **5**, (2016).

20. P. J. Keller, A. D. Schmidt, J. Wittbrodt, and E. H. K. Stelzer, "Reconstruction of zebrafish early embryonic development by scanned light sheet microscopy," Science (80-.). **322**, 1065–1069 (2008).

21. J. N. Stirman, I. T. Smith, M. W. Kudenov, and S. L. Smith, "Wide field-of-view, multi-region, two-photon imaging of neuronal activity in the mammalian brain," Nat. Biotechnol. **34**, 857–862 (2016).

22. B. Migliori, M. S. Datta, C. Dupre, M. C. Apak, S. Asano, R. Gao, E. S. Boyden, O. Hermanson, R. Yuste, and R. Tomer, "Light sheet theta microscopy for rapid high-resolution imaging of large biological samples," BMC Biol. **16**, 57 (2018).

23. T. C. Schlichenmeyer, M. Wang, K. N. Elfer, and J. Q. Brown, "Video-rate structured illumination microscopy for high-throughput imaging of large tissue areas," Biomed. Opt. Express **5**, 366 (2014).

24. D. Xu, T. Jiang, A. Li, B. Hu, Z. Feng, H. Gong, S. Zeng, and Q. Luo, "Fast optical sectioning obtained by structured illumination microscopy using a digital mirror device," J. Biomed. Opt. **18**, 060503 (2013).

25. F. B. Legesse, O. Chernavskaia, S. Heuke, T. Bocklitz, T. Meyer, J. Popp, and R. Heintzmann, "Seamless stitching of tile scan microscope images," J. Microsc. **258**, 223–232 (2015).

26. S. Preibisch, S. Saalfeld, and P. Tomancak, "Globally optimal stitching of tiled 3D microscopic image acquisitions," Bioinformatics **25**, 1463–1465 (2009).

27. C. Murtin, C. Frindel, D. Rousseau, and K. Ito, "Image processing for precise three-dimensional registration and stitching of thick high-resolution laser-scanning microscopy image stacks," Comput. Biol. Med. **92**, 22–41 (2018).

28. J. Chalfoun, M. Majurski, T. Blattner, K. Bhadriraju, W. Keyrouz, P. Bajcsy, and M. Brady, "MIST: Accurate and Scalable Microscopy Image Stitching Tool with Stage Modeling and Error Minimization," Sci. Rep. **7**, 4988 (2017).

29. Y. Yu and H. Peng, "Automated high speed stitching of large 3D microscopic images," in *2011 IEEE International Symposium on Biomedical Imaging: From Nano to Macro* (IEEE, 2011), pp. 238–241.

30. A. Bria and G. Iannello, "TeraStitcher - A tool for fast automatic 3D-stitching of teravoxel-sized microscopy images," BMC Bioinformatics **13**, 316 (2012).

31. F. Yang, Z.-S. Deng, and Q.-H. Fan, "A method for fast automated microscope image stitching," Micron **48**,

17–25 (2013).

32. P. Thévenaz and M. Unser, "User-friendly semiautomated assembly of accurate image mosaics in microscopy," Microsc. Res. Tech. **70**, 135–146 (2007).

33. P. Křížek, T. Lukeš, M. Ovesný, K. Fliegel, and G. M. Hagen, "SIMToolbox: A MATLAB toolbox for structured illumination fluorescence microscopy," Bioinformatics **32**, 318–320 (2015).

34. Microsoft, "Image Composite Editor," https://www.microsoft.com/en-us/research/product/computational-photography-applications/image-composite-editor/#!support.

35. C. A. Schneider, W. S. Rasband, and K. W. Eliceiri, "NIH Image to ImageJ: 25 years of image analysis.," Nat. Methods **9**, 671–5 (2012).

36. M. Wang, D. B. Tulman, A. B. Sholl, H. Z. Kimbrell, S. H. Mandava, K. N. Elfer, S. Luethy, M. M. Maddox, W. Lai, B. R. Lee, and J. Q. Brown, "Gigapixel surface imaging of radical prostatectomy specimens for comprehensive detection of cancer-positive surgical margins using structured illumination microscopy," Sci. Rep. **6**, 27419 (2016).

37. J. L. Dobbs, H. Ding, A. P. Benveniste, H. M. Kuerer, S. Krishnamurthy, W. Yang, and R. Richards-Kortum, "Feasibility of confocal fluorescence microscopy for real-time evaluation of neoplasia in fresh human breast tissue," J. Biomed. Opt. **18**, 106016 (2013).

38. D. S. Gareau, "Feasibility of digitally stained multimodal confocal mosaics to simulate histopathology," J. Biomed. Opt. **14**, 034050 (2009).

39. M. T. Tilli, M. C. Cabrera, A. R. Parrish, K. M. Torre, M. K. Sidawy, A. L. Gallagher, E. Makariou, S. A. Polin, M. C. Liu, and P. A. Furth, "Real-time imaging and characterization of human breast tissue by reflectance confocal microscopy," J. Biomed. Opt. **12**, 051901 (2007).

40. A. Parrish, E. Halama, M. T. Tilli, M. Freedman, and P. A. Furth, "Reflectance confocal microscopy for characterization of mammary ductal structures and development of neoplasia in genetically engineered mouse models of breast cancer," J. Biomed. Opt. **10**, 051602 (2005).

41. Y. Chen, W. Xie, A. K. Glaser, N. P. Reder, C. Mao, S. M. Dintzis, J. C. Vaughan, and J. T. C. Liu, "Rapid pathology of lumpectomy margins with open-top light-sheet (OTLS) microscopy," Biomed. Opt. Express **10**, 1257 (2019).

42. J. Dobbs, S. Krishnamurthy, M. Kyrish, A. P. Benveniste, W. Yang, and R. Richards-Kortum, "Confocal

fluorescence microscopy for rapid evaluation of invasive tumor cellularity of inflammatory breast carcinoma core needle biopsies," Breast Cancer Res. Treat. **149**, 303–310 (2015).

43. G. M. Hagen, W. Caarls, K. A. Lidke, A. H. B. De Vries, C. Fritsch, B. G. Barisas, D. J. Arndt-Jovin, and T. M. Jovin, "Fluorescence recovery after photobleaching and photoconversion in multiple arbitrary regions of interest using a programmable array microscope," Microsc. Res. Tech. **72**, 431–440 (2009).

44. S. R. Kantelhardt, W. Caarls, A. H. B. de Vries, G. M. Hagen, T. M. Jovin, W. Schulz-Schaeffer, V. Rohde, A. Giese, and D. J. Arndt-Jovin, "Specific Visualization of Glioma Cells in Living Low-Grade Tumor Tissue," PLoS One **5**, e11323 (2010).

45. L. Shao, P. Kner, E. H. Rego, and M. G. L. Gustafsson, "Super-resolution 3D microscopy of live whole cells using structured illumination," Nat. Methods **8**, 1044–1046 (2011).

46. B.-C. Chen, W. R. Legant, K. Wang, L. Shao, D. E. Milkie, M. W. Davidson, C. Janetopoulos, X. S. Wu, J. A. Hammer, Z. Liu, B. P. English, Y. Mimori-Kiyosue, D. P. Romero, A. T. Ritter, J. Lippincott-Schwartz, L. Fritz-Laylin, R. D. Mullins, D. M. Mitchell, J. N. Bembenek, A.-C. Reymann, R. Bohme, S. W. Grill, J. T. Wang, G. Seydoux, U. S. Tulu, D. P. Kiehart, and E. Betzig, "Lattice light-sheet microscopy: Imaging molecules to embryos at high spatiotemporal resolution," Science (80-.). **346**, 1257998 (2014).

47. G. M. Hagen, W. Caarls, M. Thomas, A. Hill, K. A. Lidke, B. Rieger, C. Fritsch, B. van Geest, T. M. Jovin, and D. J. Arndt-Jovin, "Biological applications of an LCoS-based programmable array microscope (PAM)," in D. L. Farkas, R. C. Leif, and D. V. Nicolau, eds. (2007), p. 64410S.

48. H. Hurwitz, "Entropy reduction in Bayesian analysis of measurements," Phys. Rev. A **12**, 698–706 (1975).

49. P. J. Verveer and T. M. Jovin, "Efficient superresolution restoration algorithms using maximum a posteriori estimations with application to fluorescence microscopy," J. Opt. Soc. Am. A **14**, 1696 (1997).

50. Michael Schmid, "Fast Filters," https://imagejdocu.tudor.lu/plugin/filter/fast_filters/start.

51. K. Fliegel, M. Klíma, and J. Pospíšil, "Assessing resolution in live cell structured illumination microscopy," in *Photonics, Devices, and Systems VII*, P. Páta and K. Fliegel, eds. (SPIE, 2017), Vol. 10603, p. 39.

52. T. C. Schlichenmeyer, M. Wang, C. Wenk, J. Q. Brown, and J. Q. Brown, "Autofocus optimization for tracking tissue surface topography in large-area mosaicking structured illumination microscopy," in *Frontiers in Optics 2014* (OSA, 2014), p. FM4F.3.

53. H. L. Fu, J. L. Mueller, M. P. Javid, J. K. Mito, D. G. Kirsch, N. Ramanujam, and J. Q. Brown,

"Optimization of a Widefield Structured Illumination Microscope for Non-Destructive Assessment and Quantification of Nuclear Features in Tumor Margins of a Primary Mouse Model of Sarcoma," PLoS One **8**, e68868 (2013).

54. H. L. Fu, J. L. Mueller, M. J. Whitley, D. M. Cardona, R. M. Willett, D. G. Kirsch, J. Q. Brown, and N. Ramanujam, "Structured Illumination Microscopy and a Quantitative Image Analysis for the Detection of Positive Margins in a Pre-Clinical Genetically Engineered Mouse Model of Sarcoma," PLoS One **11**, e0147006 (2016).

FIGURE CAPTIONS

Figure 1: Simplified diagram of SIM system. LCOS, liquid crystal on silicon

Figure 2: Vignetting artifacts and their removal. (a) shows the result of stitching images without applying the devignetting process, while (b) shows a stitch of the same data after devignetting has been applied. (c) shows the average intensity projection of the images used to stitch (a), which estimates the vignette profile of each frame. This estimate can be refined by application of an edge-limited blurring filter, as shown in (d). (e) shows the average intensity projection of the data used in (b), after devignetting has been applied. The uniform brightness of (e) indicates that no major vignetting artifacts remain in the devignetted data.

Figure 3. Panoramic SIM data processing workflow. Devignetting was performed after SIM reconstruction. Note the vignette profile differs between reconstruction methods, necessitating separate projection, blurring and division steps. Av Int Proj refers to average intensity projection.

Figure 4. Evaluating image resolution. (a) and (b) show a tile from the data in Fig. 7 (basal cell carcinoma sample) after widefield and MAP-SIM reconstruction, respectively. (c) and (d) each show a zoom-in of (a) and (b), respectively. (e) and (f) each show the FFT of (a) and (b), respectively. The dotted lines in (e) and (f) indicate the resolution of each image according to the resolution measurement described.

Figure 5. Normalized, radially averaged power spectral density (PSD_{ca}) and resolution analysis measured on the tiles shown in Figs. 4a and 4b.

Figure 6: Carcinoma of human prostate. (a) Color overview, (b) WF stitch, (c) MAP-SIM stich. (d) shows a region of the sample indicated in (a). (e) and (f) each show a zoom-in of (b) and (c), respectively, in the region indicated in (a).

Figure 7: Basal Cell Carcinoma. (a) Color overview, (b) WF stitch, (c) MAP-SIM stich. (d) shows a region of the sample indicated in (a). (e) and (f) each show a zoom-in of (b) and (c), respectively, in the region indicated in (a).

Figure 8: Adenocarcinoma of human ovary. (a) Color overview, (b) WF stitch, (c) MAP-SIM stich. (d), (g) show a region of the sample indicated in (a), acquired separately from (a) using a 10× objective. (e) and (h) show a zoom-in of (b), while (f) and (i) show a zoom-in of (c), all in the regions indicated in (a).

Figure 9: Adenocarcinoma of human breast. (a) Color overview, (b) WF stitch, (c) MAP-SIM stich. (d) shows a region of the sample indicated in (a), acquired separately from (a) using a 10× objective. (e) and (f) each show a zoom-in of (b) and (c), respectively, in the region indicated in (a).

Figure 10: Tuberculosis of human lung. (a) Color overview, (b) WF stitch, (c) MAP-SIM stich. (d) shows a region of the sample indicated in (a), acquired separately from (a) using a 20× objective. (e) and (f) each show a zoom-in of (b) and (c), respectively, in the region indicated in (a).

Figure 1

Click here to download Figure Figure01.png

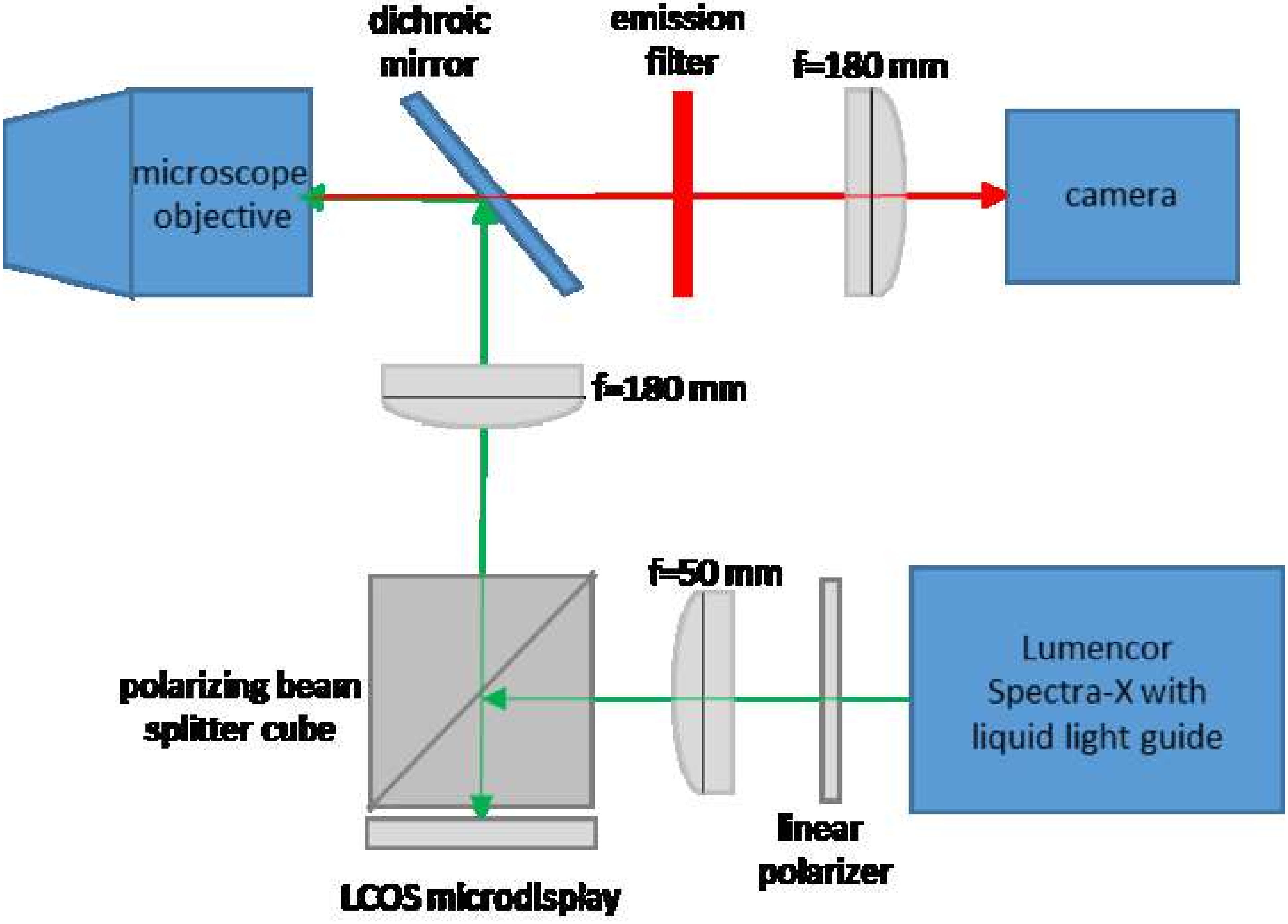

Figure 2

Click here to download Figure Figure02.png

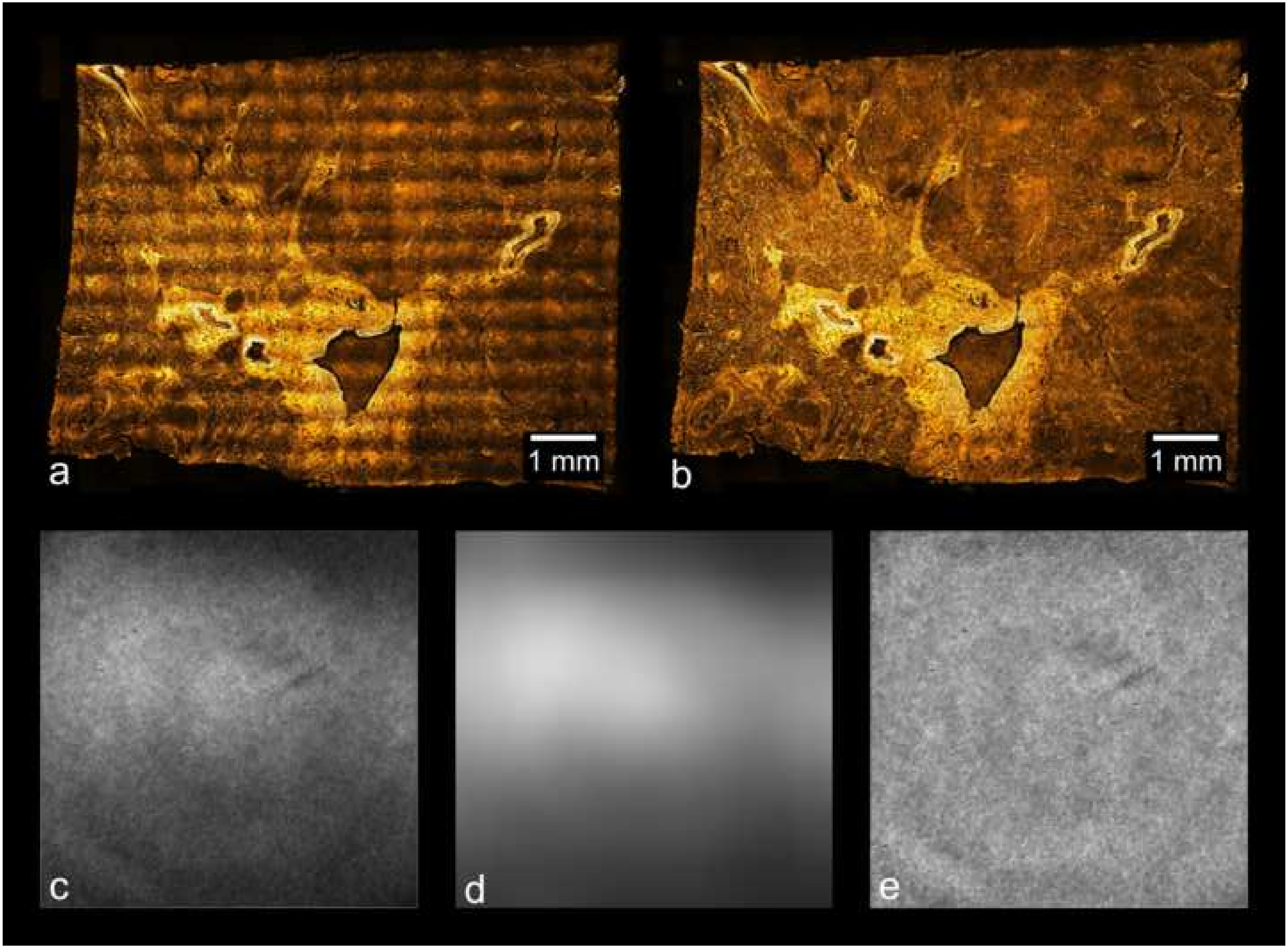

Figure 3

Click here to download Figure Figure03.png

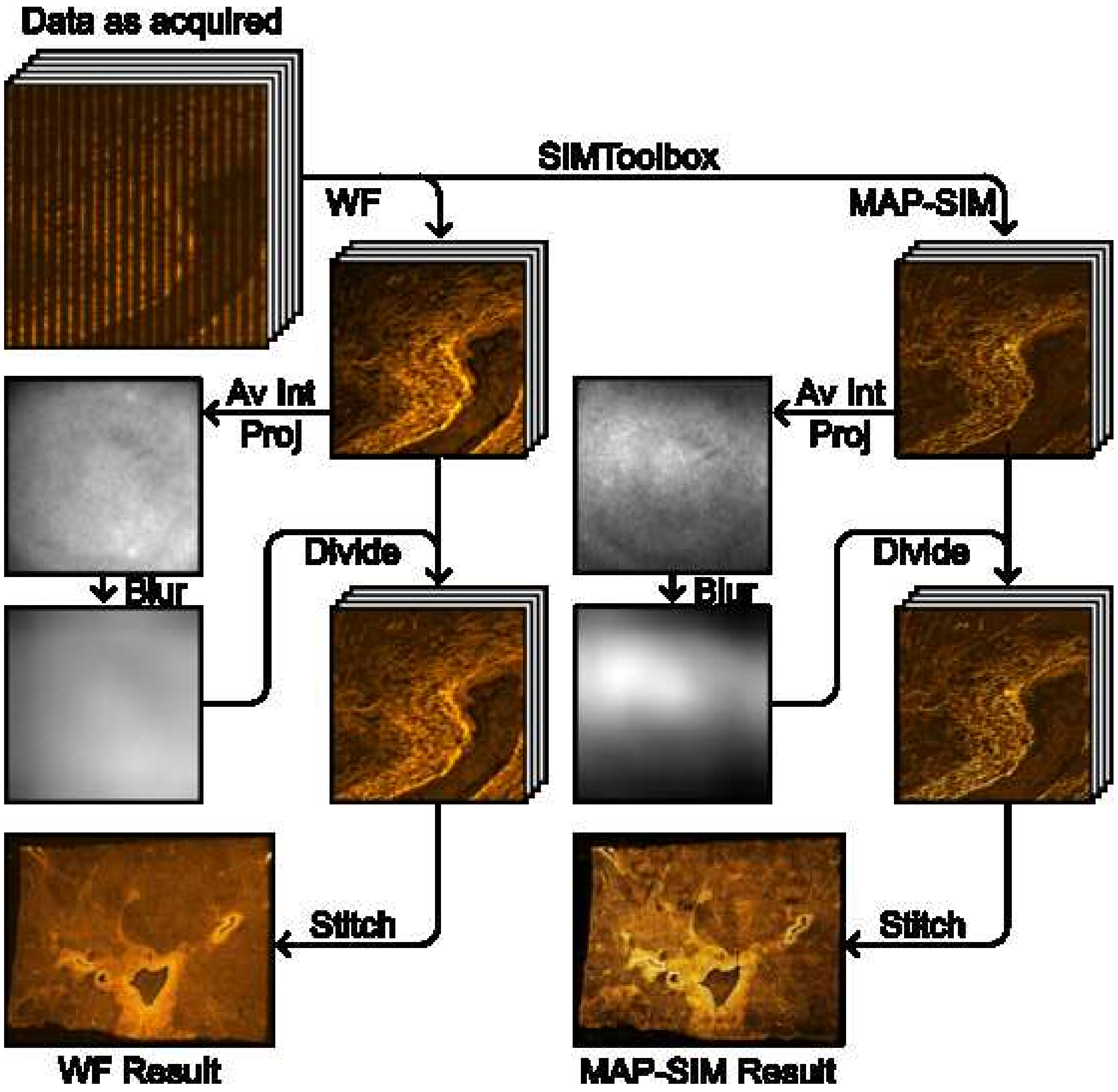

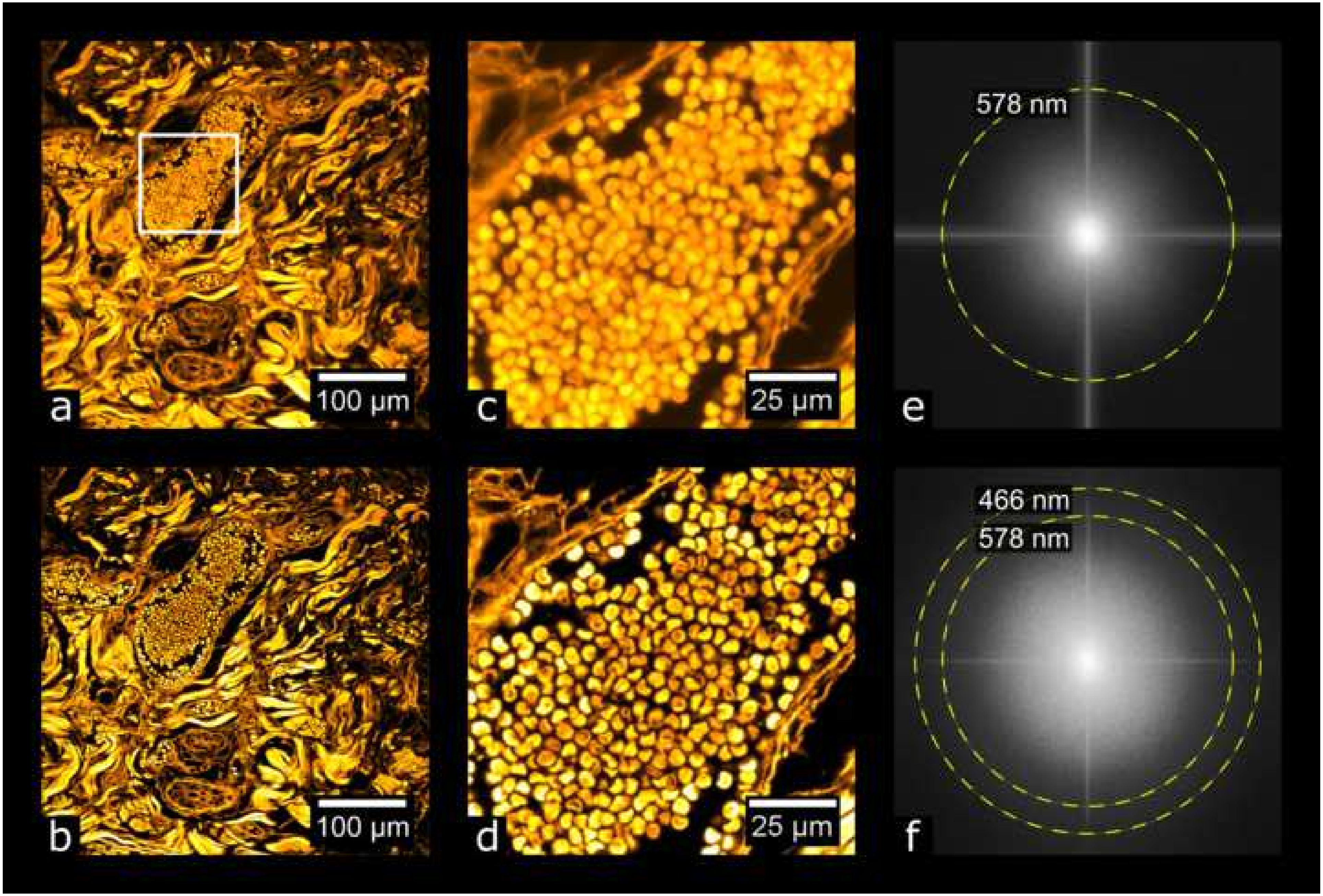
578 nm
466 nm
578 nm
100 μm
100 μm
25 μm
25 μm
a
b
c
d
e
f

Figure 5

Click here to download Figure Figure05.png

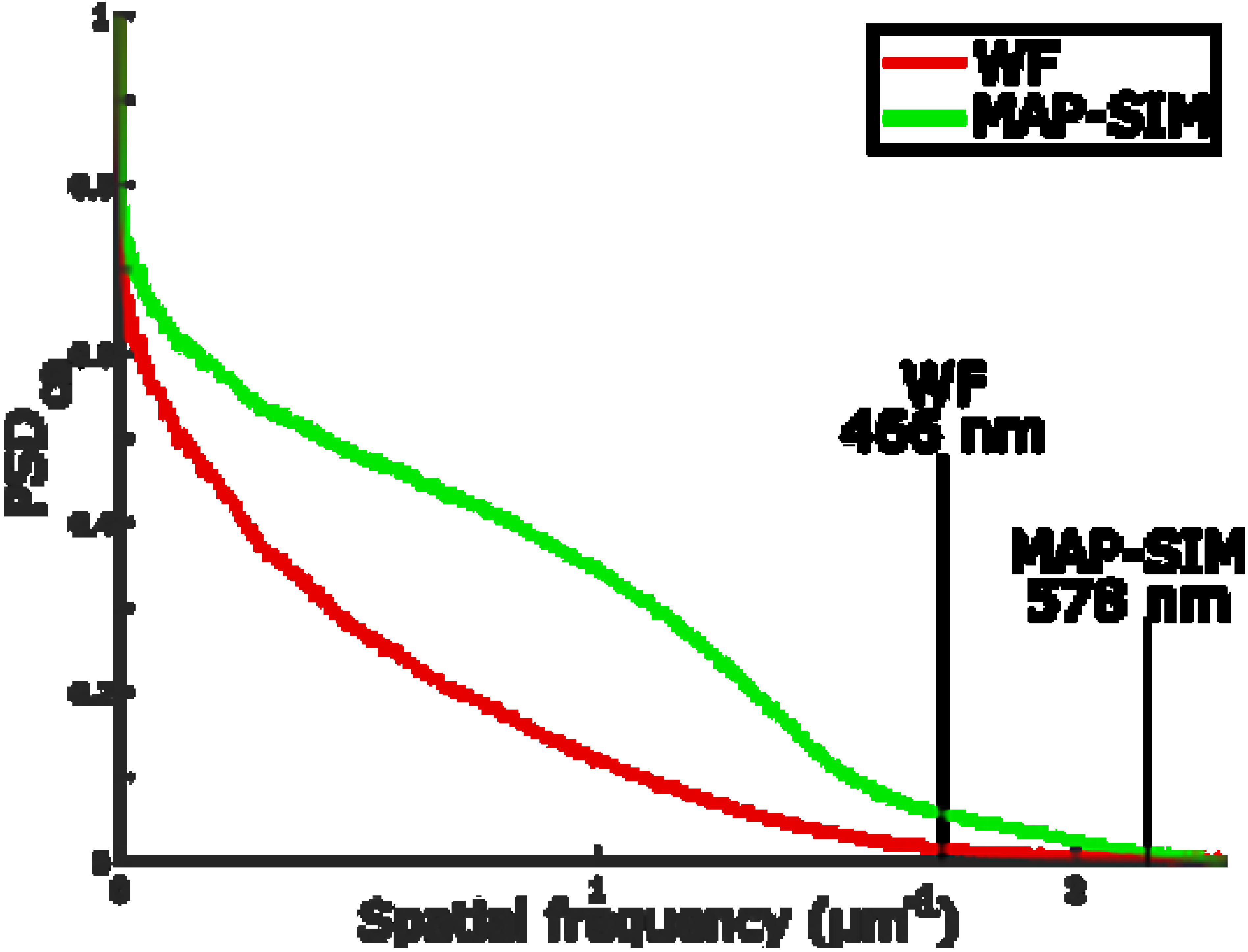

Figure 6

Click here to download Figure Figure06.png

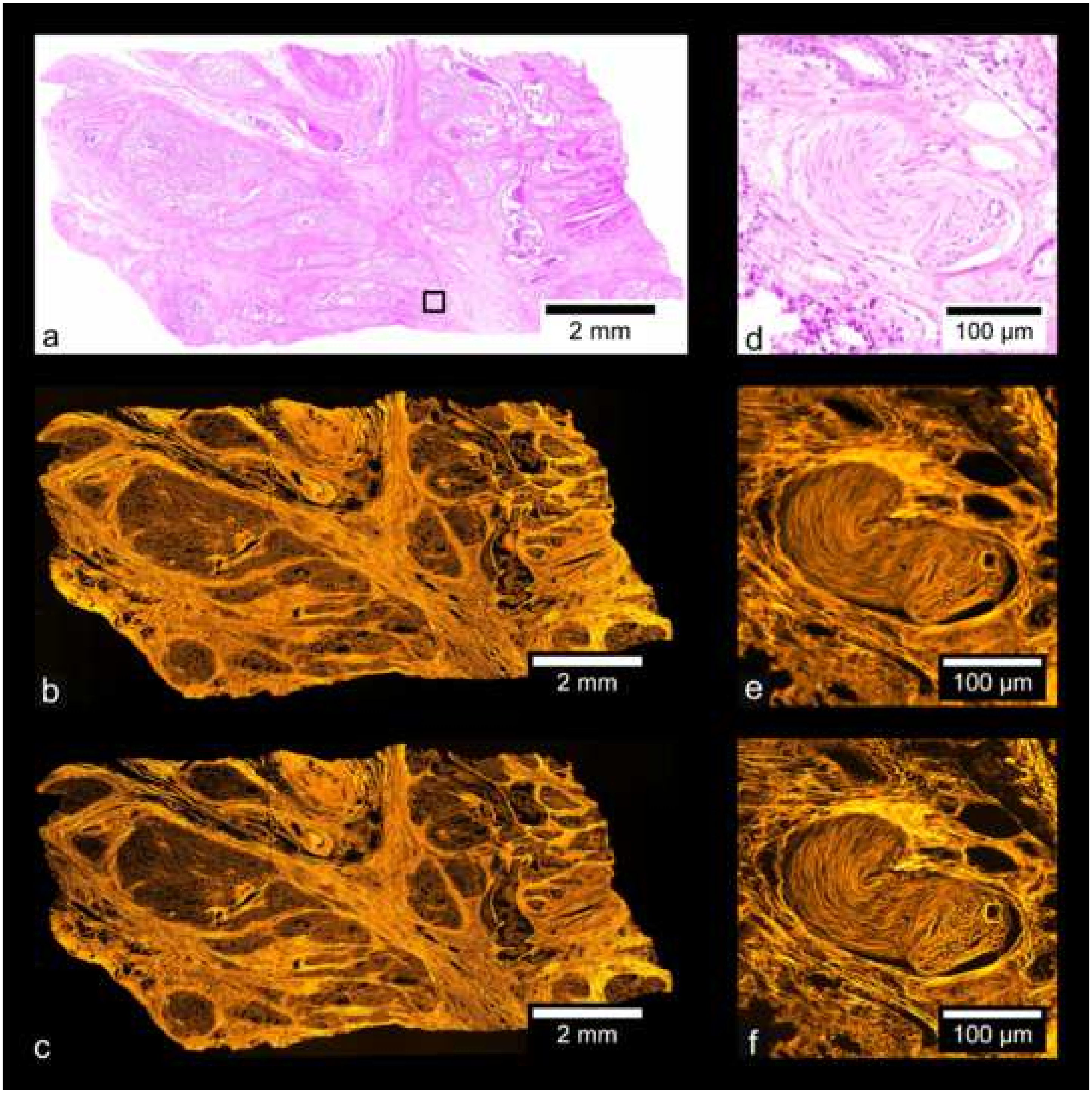

Figure 7

Click here to download Figure Figure07.png

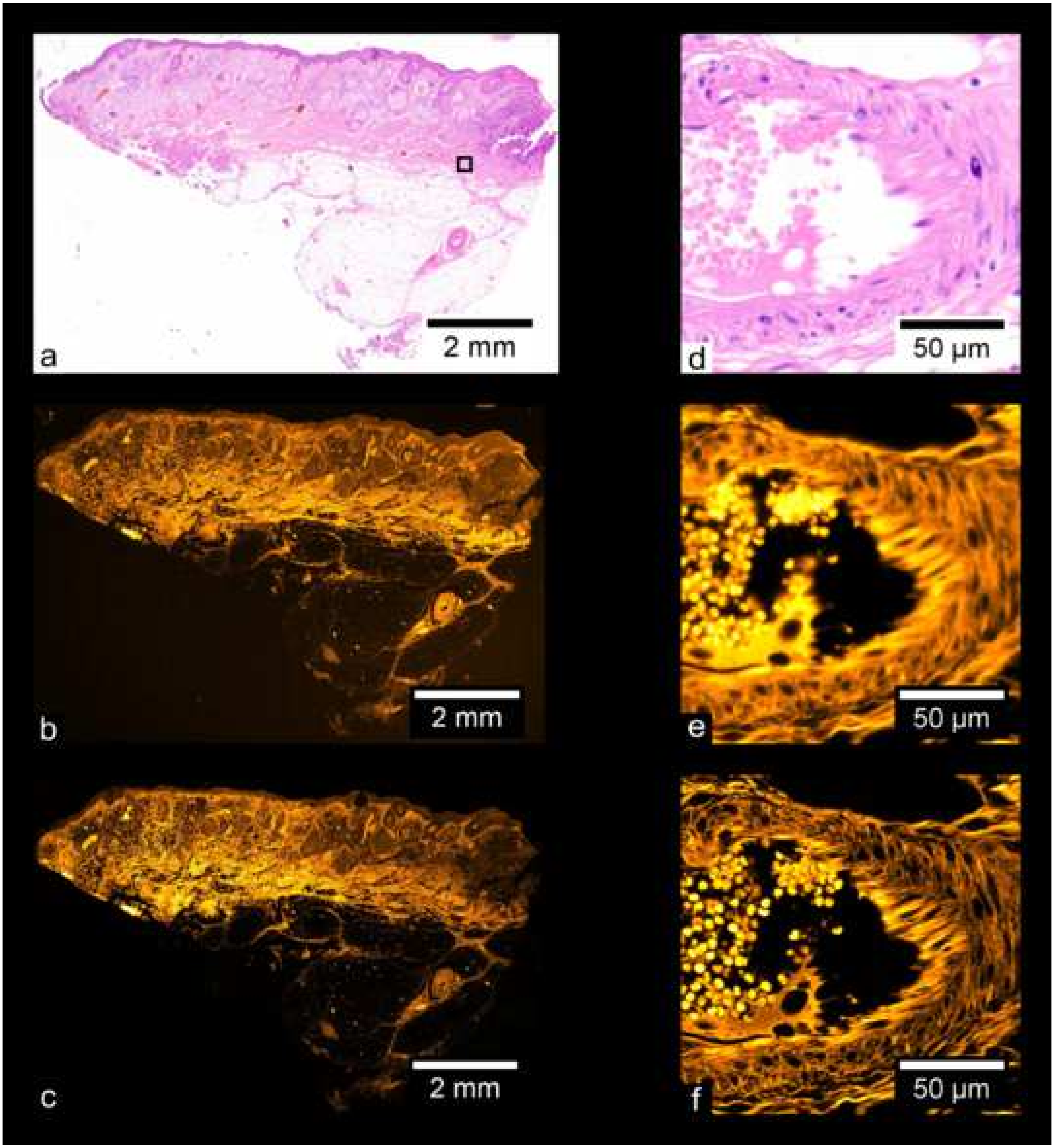

Figure 8

Click here to download Figure Figure08.png

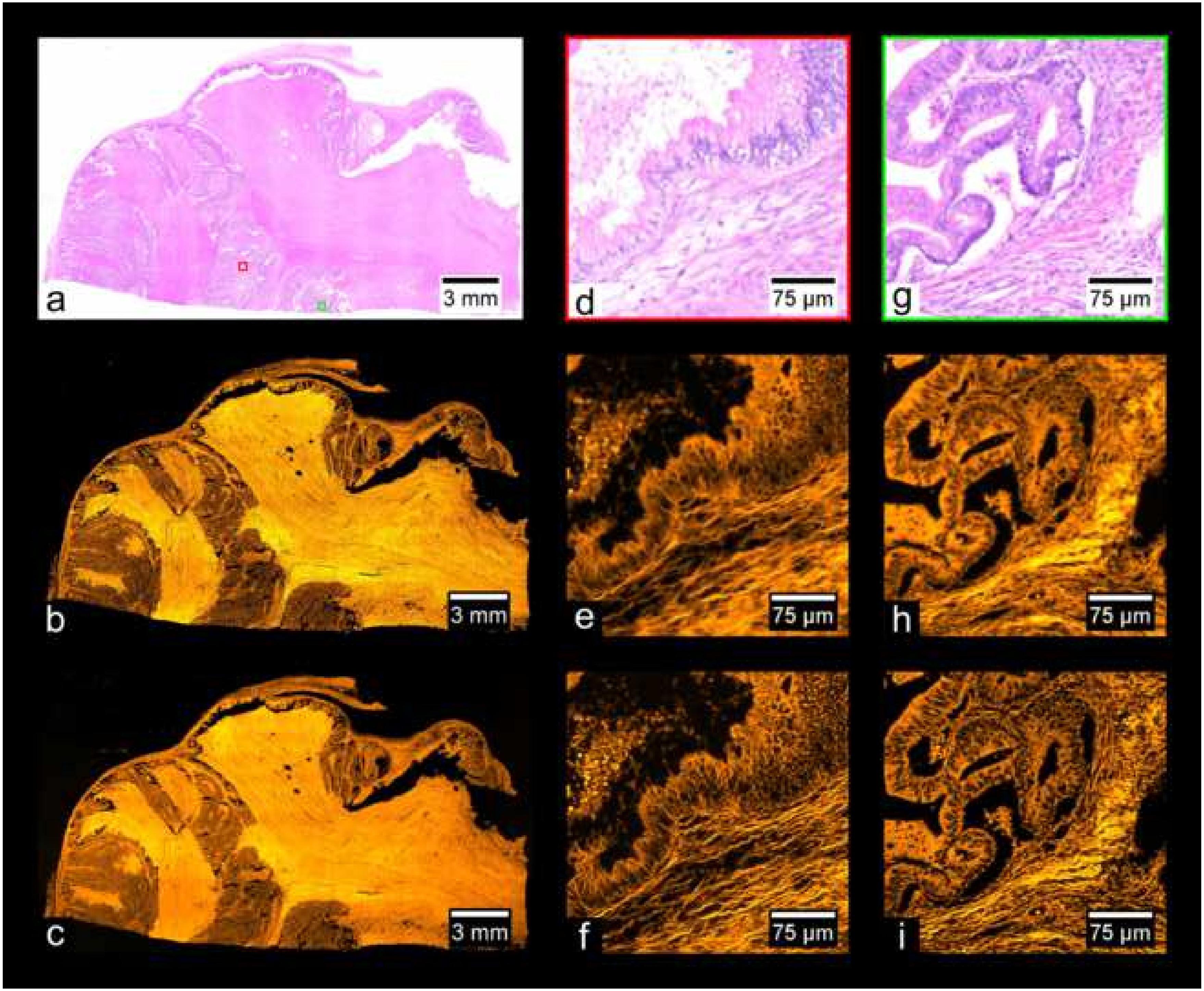

Figure 9

Click here to download Figure Figure09.png

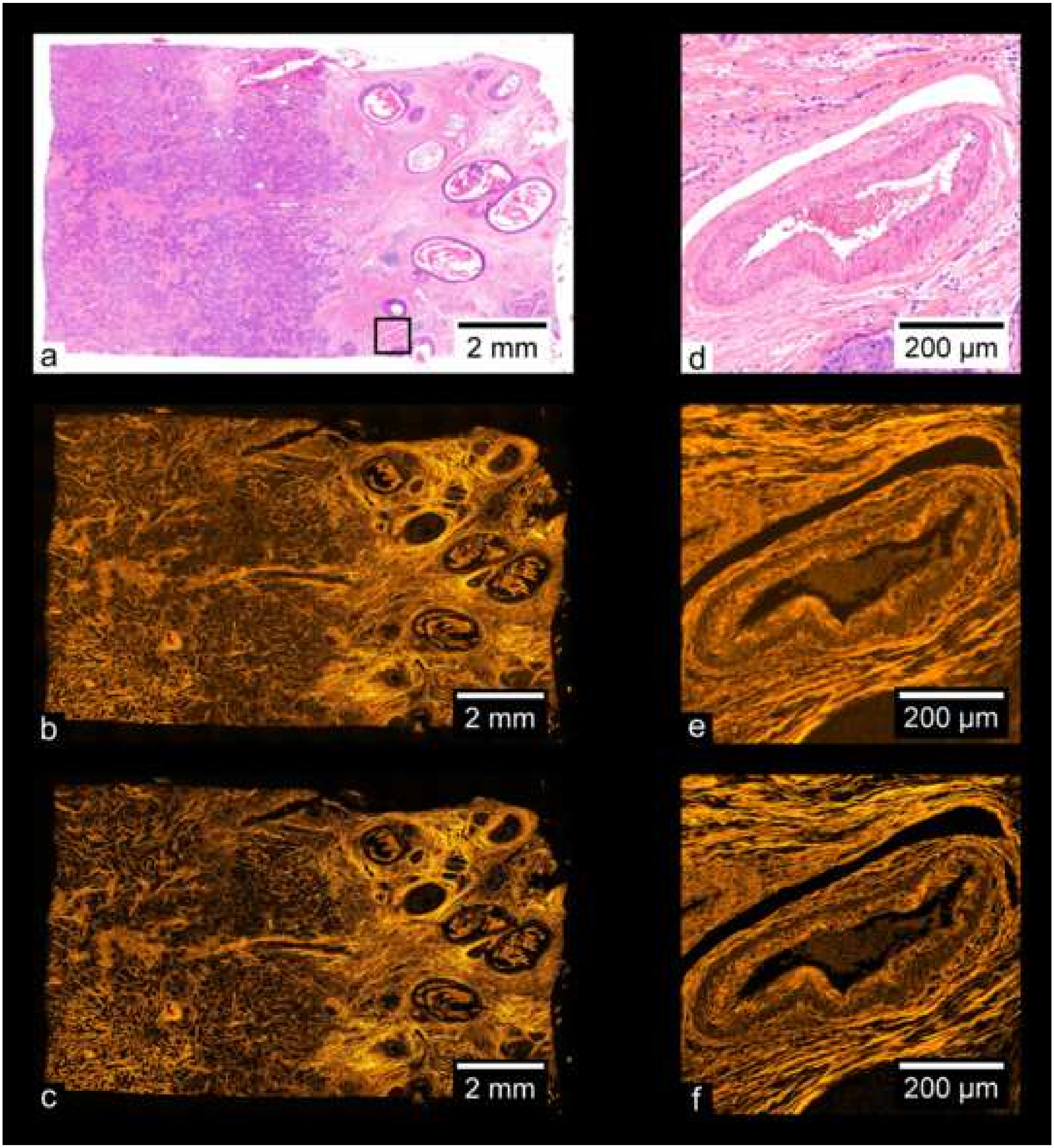

Figure 10

Click here to download Figure Figure10.png

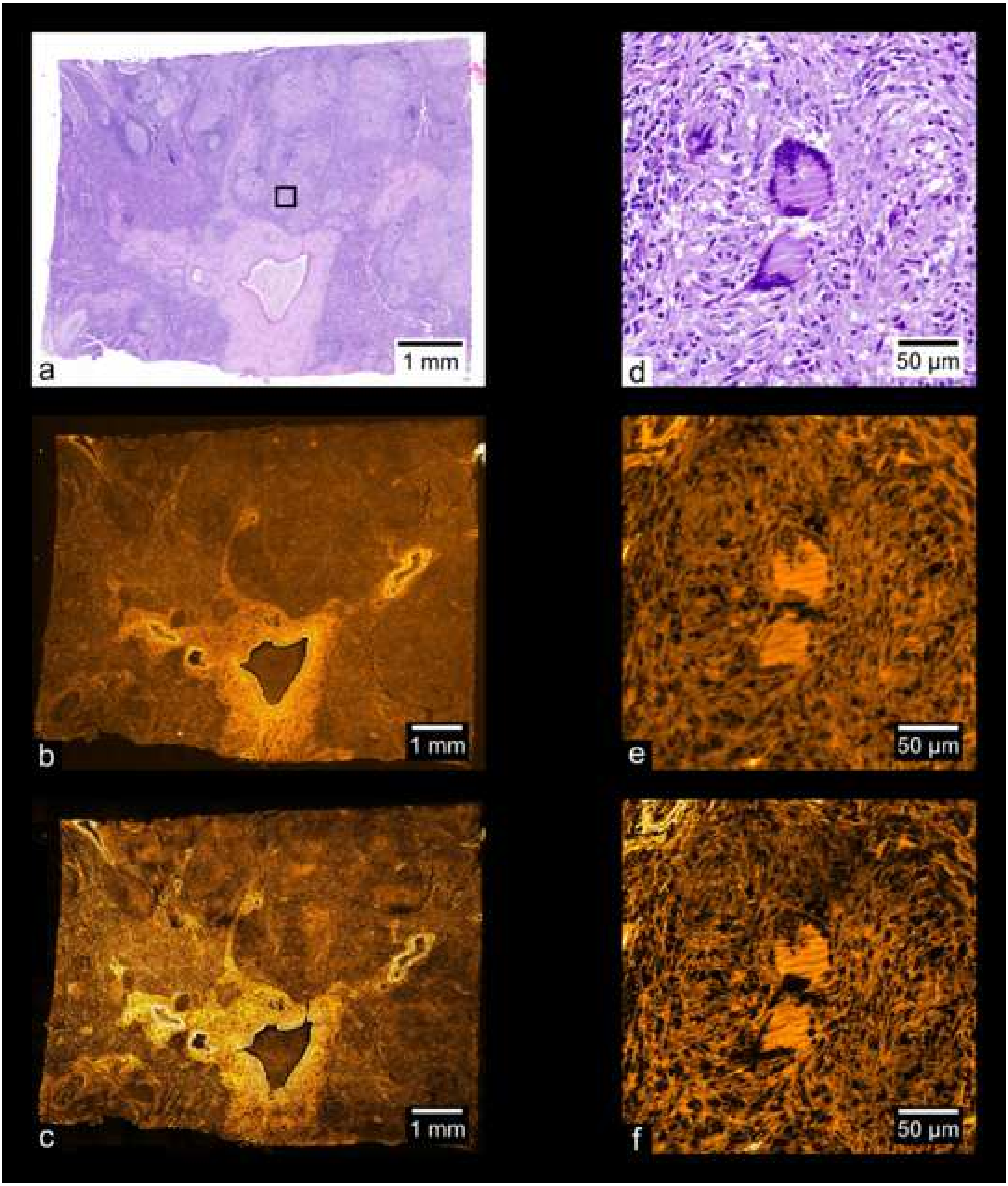

supplement

Click here to access/download
Supplementary Material
Supplement.docx

University of Colorado Colorado Springs

Dr. Guy M. Hagen
Biofrontiers
1420 Austin Bluffs Pkwy.
Colorado Springs, CO 80918
Tel. 719-255-3692
ghagen@uccs.edu

Dear Editor:

We would like to submit a manuscript entitled "Artifact-free whole-slide imaging with structured illumination microscopy and Bayesian image reconstruction" for consideration in *GigaScience* as a data note.

Fluorescence microscopy is finding increased use in histopathology. Structured illumination microscopy (SIM) is a method which can produce fluorescence images with optical sectioning (similar to confocal microscopy), and/or with super-resolution. Methods in which a larger sample is imaged with subsequent stitching of image tiles is a popular approach, but previously published data often contains noticeable artifacts due to image stitching. This is often visible as dark bands surrounding each sub-image of a larger mosaic. Other artifacts can arise due to SIM methods as they are typically applied.

Here, we present datasets which do not contain such artifacts and the detailed methods we developed to produce them. We imaged human tissues under pathophysiological conditions, including cancers of the prostate, breast, skin, and ovary. The samples we used were acquired commercially (for example from Carolina Biological) and are standard histological preparations.

We plan to upload our datasets to the Giga-database after an invitation to do so from the Journal. These datasets include the un-stitched image tiles, as well as high quality, high resolution images of the samples after de-vignetting and stitching. These datasets will be useful for other researchers investigating SIM methods or image stitching approaches, and also for clinicians who are interested in fluorescence-based histopathology. These datasets can be rather large, which has prevented their publication up to now. We believe *GigaScience* offers an excellent venue for such publication.

We would like to suggest the following reviewers for the manuscript:

Dr. Daniel Sage
Biomedical Imaging Laboratory
École polytechnique fédérale de Lausanne
Lausanne, Swizerland
daniel.sage@epfl.ch

Dr. Chris Armit
Data Scientist
Gigascience

Dr. Pavel Matula
Vice Dean, Faculty of informatics
Masaryk University
Brno, Czech Republic
pam@fi.muni.cz

University of Colorado
Colorado Springs

Dr. Guy M. Hagen
Biofrontiers
1420 Austin Bluffs Pkwy.
Colorado Springs, CO 80918
Tel. 719-255-3692
ghagen@uccs.edu

Dr. Daniel Gareau
Laboratory for Investigative Dermatology
The Rockefeller University
New York, NY, USA
dgareau@rockefeller.edu

Sincerely,

Guy Hagen

Guy M. Hagen
