# Supplementary material for: Artifact-free whole-slide imaging with structured illumination microscopy and Bayesian image reconstruction
Source: Gigascience. 2020 Apr 14;9(4):giaa035. doi: 10.1093/gigascience/giaa035 (PMC7155289; doi:10.1093/gigascience/giaa035)
Supplement: giaa035_GIGA-D-19-00306_Revision_2 [file giaa035_giga-d-19-00306_revision_2.pdf]

GigaScience

Artifact-free whole-slide imaging with structured illumination microscopy and Bayesian image reconstruction

--Manuscript Draft--

**Manuscript Number:**	GIGA-D-19-00306R2
**Full Title:**	Artifact-free whole-slide imaging with structured illumination microscopy and Bayesian image reconstruction
**Article Type:**	Data Note
**Funding Information:**	National Institute of General Medical Sciences (1R15GM128166-01) — Dr Guy M Hagen<br>Division of Biological Infrastructure (1727033) — Dr Guy M Hagen
**Abstract:**	Background<br><br>Structured illumination microscopy (SIM) is a method which can be used to image biological samples and can achieve both optical sectioning and super-resolution effects. Optimization of the imaging setup and data processing methods results in high quality images without artifacts due to mosaicking or due to the use of SIM methods. Reconstruction methods based on Bayesian estimation can be used to produce images with a resolution beyond that dictated by the optical system.<br><br>Findings<br><br>Five complete datasets are presented including large panoramic SIM images of human tissues in pathophysiological conditions. Cancers of the prostate, skin, ovary, and breast, as well as tuberculosis of the lung, were imaged using SIM. The samples are available commercially and are standard histological preparations stained with hematoxylin and eosin.<br><br>Conclusion<br><br>The use of fluorescence microscopy is increasing in histopathology. There is a need for methods which reduce artifacts when employing image stitching methods or optical sectioning methods such as SIM. Stitched SIM images produce results which may be useful for intraoperative histology. Releasing high quality, full slide images and related data will aid researchers in furthering the field of fluorescent histopathology.
**Corresponding Author:**	Guy M Hagen, PHD<br><br>UNITED STATES
**Corresponding Author Secondary Information:**	
**Corresponding Author's Institution:**	
**Corresponding Author's Secondary Institution:**	
**First Author:**	Karl A Johnson
**First Author Secondary Information:**	
**Order of Authors:**	Karl A Johnson<br>Guy M Hagen, PHD
**Order of Authors Secondary Information:**	
**Response to Reviewers:**	Dear Editor:<br>We would like to re-submit our manuscript entitled “Artifact-free whole-slide imaging with structured illumination microscopy and Bayesian image reconstruction” for

Powered by Editorial Manager® and ProduXion Manager® from Aries Systems Corporation

	consideration in GigaScience as a data note. We made the following revisions.<br><br>1. The reviewer noticed that two of the data files was incomplete or corrupted. We reloaded these two files.<br><br>2. We included a table showing the organization of the data files along with their sizes.<br><br>3. We included a universal public domain dedication text file with the dataset.<br><br>4. We added a statement about ethics approval and consent to participate.<br><br>5. We moved all of the information from the supplement into the main paper. We removed the example of the cellular-scale imaging.<br><br>Please note that the figures in the automatically generated PDF look terrible as usual. Two of the figures look like blank black boxes. There is nothing I can do about this. Please click the links embedded in the PDF to view the figures.<br><br>We hope that our paper will now be acceptable for publication in GigaScience.<br><br>Sincerely,<br><br>Guy M. Hagen
**Additional Information:**	
**Question**	**Response**
Are you submitting this manuscript to a special series or article collection?	No
**Experimental design and statistics**<br><br>Full details of the experimental design and statistical methods used should be given in the Methods section, as detailed in our Minimum Standards Reporting Checklist. Information essential to interpreting the data presented should be made available in the figure legends.<br><br>Have you included all the information requested in your manuscript?	Yes
**Resources**<br><br>A description of all resources used, including antibodies, cell lines, animals and software tools, with enough information to allow them to be uniquely identified, should be included in the Methods section. Authors are strongly encouraged to cite Research Resource Identifiers (RRIDs) for antibodies, model organisms and tools, where possible.	Yes

Powered by Editorial Manager® and ProduXion Manager® from Aries Systems Corporation

Have you included the information requested as detailed in our Minimum Standards Reporting Checklist?	
**Availability of data and materials**<br><br>All datasets and code on which the conclusions of the paper rely must be either included in your submission or deposited in publicly available repositories (where available and ethically appropriate), referencing such data using a unique identifier in the references and in the “Availability of Data and Materials” section of your manuscript.<br><br>Have you have met the above requirement as detailed in our Minimum Standards Reporting Checklist?	Yes

Manuscript

Click here to access/download;Manuscript;Hagen-Manuscript-revised-final.docx

Artifact-free whole-slide imaging with structured illumination microscopy and Bayesian image reconstruction

Karl A Johnson[1], Guy M. Hagen[1*]

[1]UCCS center for the Biofrontiers Institute, University of Colorado at Colorado Springs

1420 Austin Bluffs Parkway, Colorado Springs, Colorado, 80918, USA

*ghagen@uccs.edu

Guy Hagen: 0000-0002-4802-9481

Abstract

Background Structured illumination microscopy (SIM) is a method which can be used to image biological samples and can achieve both optical sectioning and super-resolution effects. Optimization of the imaging setup and data processing methods results in high quality images without artifacts due to mosaicking or due to the use of SIM methods. Reconstruction methods based on Bayesian estimation can be used to produce images with a resolution beyond that dictated by the optical system.

Findings Five complete datasets are presented including large panoramic SIM images of human tissues in pathophysiological conditions. Cancers of the prostate, skin, ovary, and breast, as well as tuberculosis of the lung, were imaged using SIM. The samples are available commercially and are standard histological preparations stained with hematoxylin and eosin.

Conclusion The use of fluorescence microscopy is increasing in histopathology. There is a need for methods which reduce artifacts when employing image stitching methods or optical sectioning methods such as SIM. Stitched SIM images produce results which may be useful for intraoperative histology. Releasing high quality, full slide images and related data will aid researchers in furthering the field of fluorescent histopathology.

Keywords Structured illumination microscopy, SIM, image stitching, Bayesian methods, MAP-SIM, SIMToolbox, histopathology, cancer.

Data description

Context

Structured illumination microscopy (SIM) is a method in optical fluorescence microscopy which can achieve both optical sectioning (OS-SIM) [1] and resolution beyond the diffraction limit (SR-SIM) [2,3]. SIM has been used for super-resolution imaging of both fixed and live cells [4–7] and has matured enough as a method that it is now available commercially. In SIM, a set of images is acquired using an illumination pattern which shifts between each image. As SIM has developed, diverse strategies have been proposed for creation of the SIM pattern [1,8–13]. Several different approaches for processing the data have also been introduced [3,7,8,14–16].

Recently, microscope systems capable of imaging with high resolution and a large field of view (FOV) have been developed [17–21], some using custom-made microscope objectives. However, stitching together images acquired with a higher magnification objective to create a large mosaic remains a valid and popular approach. Some published results involving stitched images suffer from pronounced artifacts in which the edges of the individual sub-images are visible, usually as dark bands which outline each sub-image [22–24]. On the other hand, several studies have proposed methods for stitching of microscope images with reduced artifacts [25–32].

The combination of SIM with image stitching methods allows collection of large FOV images with both optical sectioning and super-resolution properties. Here, we demonstrate methods and provide complete datasets for five different samples. The samples are hematoxylin and eosin (H&E) stained histological specimens which provide examples of human diseases (ovarian cancer, breast cancer, prostate cancer, skin cancer, and tuberculosis), and which are also available commercially for those who wish to reproduce our work. We used freely available optical designs [6,10,33] and open source software [33] for SIM imaging, along with freely available software for image stitching (Microsoft Image Composite Editor (ICE) [34], or a well validated plugin [26] for ImageJ [35]). Combining this with devignetting methods, we produced stitched images which are free of noticeable artifacts from stitching or from SIM reconstruction.

Fluorescence microscopy is becoming more important in histopathology. Traditional bright field microscopy diagnostic methods require a time-consuming process, involving chemical fixation and physical sectioning. The use of optical sectioning fluorescence microscopy allows high-quality images to be captured without the need for physical sectioning. Consequently, it has been shown that imaging can be performed on large human

tissue samples within 1 hour after excision [36]. Additionally, other studies have shown the results of fluorescence imaging to be usable and accurate in diagnosis of various medical conditions [37–42]. Previously, it was noted that obvious stitching artifacts significantly decrease the usability of large fluorescence images in medical diagnosis. In one case, such artifacts resulted in the rejection of over half of the images acquired [38]. The setup we describe here allows for fast, artifact-free, high-resolution imaging of fluorescent samples, and is compatible with samples stained with most fluorescent dyes.

Methods

Samples

All samples used in this study are available from Carolina Biological, Omano, or Ward's Science. The samples are approximately 7 μm thick and are stained with hematoxylin and eosin. The commercial source, product number, and other SIM imaging parameters for each sample are detailed in Table 1. Table 2 details imaging parameters for acquisitions of each sample with a color camera.

Table 1: Imaging parameters for the SIM datasets

Sample	Source company and product no.	SIM pattern no. of phases	Exposure time, ms	No. of tiles	Objective mag/NA	Acquisition time, s	Stitching software
Carcinoma of Prostate	Carolina, 318492	5	50	23 × 11	20×/0.45	315	Microsoft ICE
Basal Cell Carcinoma	Ward's Science, 470183-256	6	75	29 × 18	30×/1.05	821	FIJI
Adenocarcinoma of Ovary	Carolina, 318628	5	100	25 × 14	10×/0.4	595	Microsoft ICE
Adenocarcinoma of Breast	Carolina, 318766	8	200	12 × 8	10×/0.4	278	FIJI
Lung Tuberculosis	Omano, OMSK-HP50	5	100	20 × 16	30×/1.05	541	FIJI

Table 2: Parameters for the color images

Sample	No. of tiles	Objective mag/NA
Carcinoma of Prostate	6 × 5	4×/0.16
Basal Cell Carcinoma	5× 5	4×/0.16
Adenocarcinoma of Ovary	11 × 11	4×/0.16
Adenocarcinoma of Breast	6 × 6	4×/0.16
Lung Tuberculosis	8 × 10	10×/0.4

Microscope setup and data acquisition

We used a home-built SIM setup based on the same design as described previously [6,10,15] (Figure 1). The SIM system is based on an IX83 microscope (Olympus) equipped with a Zyla 4.2+ sCMOS camera (Andor) under the control of IQ3 software (Andor). We used the following Olympus objectives: UPLSAPO 4×/0.16 NA,

UPLSAPO 10×/0.4 NA, LUCPLFLN 20×/0.45 NA, and UPLSAPO 30×/1.05 NA silicone oil immersion. For color images we used an aca1920-40uc color camera (Basler) under control of Pylon software (Basler). We used a MS-2000 motorized microscope stage (Applied Scientific Instrumentation) to acquire tiled SIM images. In all datasets, the stage scanning was configured such that all image edges overlapped by 20%.

Briefly, the SIM system uses a ferroelectric liquid crystal on silicon (LCOS) microdisplay (type SXGA-3DM, Forth Dimension Displays). This device has been used previously in SIM and related methods in fluorescence microscopy [5,10,15,33,43–47] and allows one to produce patterns of illumination on the sample which can be reconfigured at will by changing the image displayed on the device. The light source (Lumencor Spectra-X) is toggled off between SIM patterns and during camera readout. Figure 1 shows a simplified diagram of the microscope system.

INSERT FIGURE 1-system diagram

Motorized stage and illumination control

Close synchronization between the camera acquisitions, light source, and microdisplay ensures rapid image acquisition, helps reduce artifacts, and reduces light exposure to the sample. As shown in Figure 2, Andor IQ software controls the SIM system.

INSERT FIGURE 2-connection diagram

While the camera and XYZ stage receive signals directly from Andor IQ software and an input/output computer card (Measurement Computing DDA06/16), the illumination signals which the software generates must be altered before being sent to the light source. Firstly, the microdisplay used in our setup will not produce an image on the sample if it is illuminated with a constant light source. Rather, a meaningful illumination pattern will only form if the light source is synchronized with an enable signal output from the microdisplay control board. Therefore, the channel signals output by IQ are first modulated with the microdisplay enable signal (this is performed by the leftmost AND gates pictured in Figure 2). Additionally, to reduce unnecessary light exposure to the sample, the light source is shut off whenever the camera sensor is not being exposed. This is accomplished by performing a second logical AND of the result of the previous AND with the ‘FIRE’ signal output from the camera. This process is illustrated in Figure 3.

INSERT FIGURE 3-trigger

SIM data processing

SIM reconstructions were performed in the same way as previously described using SIMToolbox, an open-source and freely available program that our group developed for processing SIM data [33]. We generated optically sectioned, enhanced resolution images using a Bayesian estimation method, maximum *a posteriori* probability SIM (MAP-SIM) [15]. MAP-SIM works using maximum *a posteriori* probability methods, which are well known in microscopy applications [48,49], to enhance high spatial frequency image information. We then combine this information, in the frequency domain, with low spatial frequency image information obtained by OS-SIM methods, then produce the final image by an inverse Fourier transform [15]. We typically measure the final resolution obtained by analyzing the frequency spectrum of the resulting image, as is discussed below.

The illumination patterns used here are generated such that the sum of all positions in each pattern set results in homogenous illumination. As such, a widefield (WF) image can be reconstructed from SIM data simply by performing an average intensity projection of the patterned images. This can be described by

$$I_{WF} = \frac{1}{N}\sum_{n=1}^{N} I_n,$$

where N is the number of pattern phases, I_n is the image acquired on the nth illumination position, and I_{WF} is the WF reconstruction. This is the method we used to generate WF images throughout this study.

Vignetting correction

Following SIM reconstruction, vignetting artifacts remain in each tile. If not removed prior to stitching, this vignetting introduces a distracting grid pattern in the final stitched image. We performed vignette removal by dividing each tile of the mosaic by an image representing the vignetting profile common to all tiles. Other studies have used an image of a uniformly fluorescent calibration slide as a reference for vignette removal [36], where information concerning non-uniform illumination is captured. However, we found that SIM processing introduces vignetting artifacts beyond those due to non-uniform illumination. Additionally, these artifacts vary somewhat depending on properties of the sample being imaged. As such, performing pre-acquisition calibration on a uniformly

fluorescent slide is not sufficient to remove vignetting artifacts from SIM reconstructions. Instead, an estimate of the vignetting profile is found through analysis of the mosaic tiles after SIM reconstruction.

A blurred average intensity projection of the tiles is a good approximation of the vignetting profile, as an average intensity projection merges the tiles into a single image with averaged foreground information while preserving the vignetting profile. Subsequent blurring with an appropriate radius and edge-handling method also eliminates the high spatial frequency foreground without impacting the low spatial frequency illumination profile. To eliminate errors during the blurring step due to the blurring area extending outside the original image, we used an edge handling method in which the blurring area is reduced near the edges of the image such that no values outside the image border are sampled. Unlike edge handling methods in which the image is padded with a uniform value (or mirrored and tiled) to accommodate a blurring area which extends beyond the original image limits, this method is free from major artifacts, such as erroneous brightness of the image edges, see figure 4. While the average intensity projection removes most foreground information from the images, some coarseness remains in the vignette estimation after this step (figure 4a), especially for stitches with fewer than 50 tiles. The blurring step serves to eliminate only this non-vignette information and must preserve the illumination profile. As shown in figure 4b, use of the default Gaussian blurring function in ImageJ introduces a bright glow near to borders of the image, a significant artifact which does not reflect the original vignetting profile. Use of the 'border limited mean' filter, on the other hand, does not introduce this aberration, as shown in figure 4c.

INSERT FIGURE 4-edge method

Approximating the illumination profile works especially well for histological samples, as such samples are non-sparse and require many tiles, factors which improve the accuracy of this approach. We performed all steps of this devignetting process using built-in functions and the 'Fast Filters' plugin in ImageJ [50]. The effect of devignetting is illustrated in Figure 5.

INSERT FIGURE 5-devignetting

Image Stitching

With visible vignetting removed, we then stitched together a composite image from the tiles. The pre-processing allows for stitching to be done in various stitching applications; Microsoft ICE and Preibisch's plugin for FIJI [26] were used to stitch the data presented here.

The data processing procedure described here is summarized in Figure 6. The total time for processing each dataset was about 30 min.

INSERT FIGURE 6-flowchart

Color image data processing methods

We created color overview images by stitching devignetted brightfield acquisitions. Devignetting was performed simply by adding the inverse of an empty brightfield acquisition to each color tile using ImageJ. For this method to produce optimal results, the empty brightfield image must be acquired in conditions identical to those of the raw tile data, such that the illumination profile in the empty image matches that of the unprocessed tiles. This simple operation removes nearly all visible vignetting and color balance artifacts within each tile. The results after devignetting were then stitched using Preibisch's plugin for FIJI [26].

Resolution measurement

We evaluated our results by measuring image resolution using SR Measure Toolbox. SR Measure Toolbox [51] measures the resolution limit of input images through analysis of the normalized, radially averaged power spectral density (PSD_{ca}) of the images, as previously described [6]. Briefly, the resolution limit in real space is determined by evaluating the cutoff frequency in Fourier space. The cutoff frequency is estimated by calculating the spatial frequency at which the PSD_{ca} (after noise correction) drops to zero.

Focusing on the basal cell carcinoma sample, we selected 125 (out of 522 total) image tiles, calculated the PSD and resolution for each tile, and averaged the results. We found that, in the case of this sample, the image resolution was 593 ± 20 nm for WF and 468 ± 2.5 nm for MAP-SIM (average ± standard deviation). This data was acquired with a UPLSAPO 30×/1.05 NA silicone oil immersion objective. Figure 7 shows an example measurement for one image tile. Figure 8 shows a plot of PSD_{ca} for this image tile.

INSERT FIGURE 7-resolution

INSERT FIGURE 8-PSD

Results

Figure 9 shows images of a prepared slide containing a human prostate carcinoma sample stained with H&E. Figure 9a shows a stitched color overview, and Figure 9d shows a zoom-in of the region indicated in Figure 9a, acquired separately using a UPLSAPO 20×/0.75NA objective. Figure 9b shows a stitched widefield fluorescence image, and Figure 9c shows a stitched SIM image. Figures 9e and 9f each show zoom-ins of the stitches shown in Figures 9b and 9c, respectively. Using the acquisition and processing methods described, whole-slide images are produced without any visible stitching artifacts. Additionally, the MAP-SIM reconstruction method produces resolution superior to that of the widefield data. Figures 10-13 show similar comparisons for basal cell carcinoma, ovary adenocarcinoma, breast adenocarcinoma, and tuberculosis of the lung, respectively.

The data shown in figures 9-13 is freely available through Giga DB[52]. This dataset includes all color overviews as well as WF and MAP-SIM stitches at full resolution. In addition, all image tiles used to create the WF and MAP-SIM stitches of the samples are provided.

INSERT FIGURE 9-prostate

INSERT FIGURE 10-basal cell

INSERT FIGURE 11-ovary

INSERT FIGURE 12-breast

INSERT FIGURE 13-lung

Discussion

Many past studies into stitching of SIM mosaics have suffered from noticeable image artifacts, arising from flaws in the optical setups used as well as imperfections in the SIM reconstruction and image stitching processes. While these artifacts are sometimes minimal enough to remain uncorrected, certain artifacts seriously inhibit the usefulness of the final stitched image. In [23], the authors note that issues in triggering and evenly illuminating the microdisplay being used for illumination resulted in striping and vignetting artifacts; similarly, in [22,24,36,53], stitching artifacts are apparent in the images.

Here, optimization of the optical setup, camera-microdisplay synchronization, and image processing methods yielded whole-slide images free from visible SIM or image stitching artifacts. In addition to the elimination of artifacts, our use of SIMToolbox to perform SIM reconstruction on the data allows for a variety of reconstruction algorithms to be used, including super-resolution algorithms such as MAP-SIM. This too presents an improvement over previous works.

Another advantage of the acquisition and processing methods demonstrated here is the minimization of user intervention, and in turn, reductions in acquisition and processing time. Stage movement, sample focusing, image acquisition, and SIM pattern advancement are controlled automatically. Loading of the sample, definition of the mosaic edges, and manual focus on 3-5 positions in the sample are the only steps needed to be taken by the user before acquisition can begin. Recent developments in autofocus technology for SIM may allow for the manual focus step to be shortened or omitted [53]. These automated steps during acquisition allow for large mosaics to be acquired. The quality of the final stitched images does not degrade for larger mosaics – in fact, the quality of the devignetting process improves with larger datasets, as more data is available to produce an accurate estimation of the illumination profile. SIMToolbox (version 2.0), which is capable of utilizing the processing power of modern consumer graphics cards during MAP-SIM processing, also reduces the time spent during the data processing phase. Finally, unlike other super-resolution reconstruction methods such as SR-SIM, MAP-SIM is able to produce artifact-free results without tuning of reconstruction parameters by the user, a process which is difficult to automate and requires significant user experience.

One drawback of the method presented here is the inability to image the entire volume of samples thicker than ~0.5 mm. However, this limitation does not prevent large, unsectioned samples from being imaged, as is the case with bright field microscopy, where samples must be thin enough for transmitted light to reach the objective. Rather, as the light which illuminates the sample in fluorescence microscopy emanates from the objective, all surface regions of a large sample may be imaged. Additionally, due to the optical sectioning exhibited by SIM, light from out-of-focus regions of the sample is almost completely attenuated. Consequently, imaging the surfaces of large samples with SIM produces high quality images without the need for physical sectioning, as previously demonstrated [23,36].

Here, we demonstrated our imaging techniques on traditionally prepared histopathological samples in order to provide a comparison between bright field imaging and SIM, but the same techniques can be used to image a wide variety of fluorescently labelled samples. The ability to seamlessly image the entire surface region of large samples has multiple potential applications in histopathology. SIM presents unique advantages in analyzing the surgical margins of large tissue excisions, as demonstrated by Wang, et al [36]. Confocal imaging of core needle biopsy samples has been previously demonstrated to produce images suitable for medical diagnosis [42], a practice easily adapted to SIM. The speed at which sample preparation and image acquisition can be performed in fluorescence microscopy presents opportunities for intra-operative analysis of tissue samples using SIM techniques, as mentioned by multiple other studies [23,36,54,55].

Reuse potential and example of data reuse

The data provided here presents various opportunities for reuse. With the multiple high-resolution color overviews and stitched SIM images, comparison of structures visible in the brightfield and fluorescent images could be performed to further study the use of fluorescence microscopy in histopathology. The unstitched image tiles of the basal cell carcinoma sample provided in the dataset, which still contain vignetting artifacts, may be used to reproduce the results of our devignetting process, as well as to further develop new devignetting approaches suited for SIM. These tiles might also be used to create or modify existing stitching software for global minimization of stitching artifacts. Note that the image tiles from the other samples in the dataset are provided after devignetting.

Gone uncorrected, vignetting in the image tiles used to stitch a larger image can cause a noticeable grid pattern in the final stitched image. While readily noticeable upon viewing of the image, quantification of this pattern is useful for evaluation of methods which remove it. Since this stitching artifact arises from an illumination profile common to each tile, the period of this pattern in the stitched image can simply be represented by the spacing between tiles used during acquisition:

$$T_{stitch} = l \cdot (1 - r),$$

where l is the image width and r is the proportional overlap between image tiles. The parameters for the dataset visualized in Figure 14 are l = 2048 px and r = 0.2, thus T_{stitch} = 1638 px, or ~355 µm (as the dataset has a pixel size of ~216.7 nm). In our setup, the camera sensor is square, so T_{stitch} is the same both the horizontally and vertically in the final image. As a pattern with a very consistent period, this grid artifact manifests in the FFT of an uncorrected

stitch as series of bright peaks. As shown in Fig 14e, the location of the peaks corresponding to the fundamental frequency of the grid pattern agrees very well with the calculated T_{stitch}. Figs. 14d and 14e show that the FFT of a properly corrected image contains no trace of the peaks evident in the uncorrected image.

INSERT FIGURE 14-analysis

Availability of source code and requirements

Project name: SIMToolbox version 2.12

Project home page: http://mmtg.fel.cvut.cz/SIMToolbox/

Operating system: platform independent

Programming language: MATLAB

License: GNU General Public License v3.0

Detailed software compatibility notes

The SIMToolbox GUI was compiled with MATLAB 2015a and tested in Windows 7 and 8. The GUI is a stand-alone program and does not require MATLAB to be installed. To use the MATLAB functions within SIMToolbox (i.e., without the GUI), MATLAB must be installed. The functions were mainly developed with 64 bit MATLAB versions 2012b, 2014a, 2015a in Windows 7. When using SIMToolbox functions without the GUI, the MATLAB "Image Processing Toolbox" is required. SIMToolbox also requires the "MATLAB YAML" package to convert MATLAB objects to/from YAML file format. Note that this package is installed automatically when using the GUI.

Availability of data

All raw and analyzed data is available on GigaDB [52]. All files and data are distributed under the Creative Commons CC0 waiver, with a request for attribution. The data is organized into five main folders for the five different samples, see table 3.

Table 3: Description of the data files

Folder	Files	File Size
01-Prostate-carcinoma	Prostate-carcinoma-WF-tiles.zip	3.6 GB
	Prostate-carcinoma-MAPSIM-tiles.zip	3.5 GB
	Prostate-carcinoma-WF-stitch.tif	695 Mb
	Prostate-carcinoma-MAPSIM-stitch.tif	695 Mb
	Prostate-carcinoma-Color-stitch.tif	118 MB
02-Basal-cell-carcinoma	Basal-cell-carcinoma-MAPSIM-tiles.zip	7.5 GB

	Basal-cell-carcinoma-WF-tiles.zip	7.5 GB
	Basal-cell-carcinoma-MAPSIM-stitch.tif	6.3 GB
	Basal-cell-carcinoma-WF-stitch.tif	5.7 GB
	Basal-cell-carcinoma-color-stitch.tif	123 MB
03-Ovary-adenocarcinoma	Ovary-adenocarcinoma-MAPSIM-tiles.zip	891 MB
	Ovary-adenocarcinoma-WF-tiles.zip	849 MB
	Ovary-adenocarcinoma-MAPSIM-stitch.tif	916 MB
	Ovary-adenocarcinoma-WF-stitch.tif	979 MB
	Ovary-adenocarcinoma-Color-stitch.tif	610 MB
04-Breast-adenocarcinoma	Breast-adenocarcinoma-MAPSIM-tiles.zip	1.4 GB
	Breast-adenocarcinoma-WF-tiles.zip	1.4 GB
	Breast-adenocarcinoma-MAPSIM-stitch.tif	1.1 GB
	Breast-adenocarcinoma-WF-stitch.tif	1.1 GB
	Breast-adenocarcinoma-Color-stitch.tif	129 MB
05-Lung-tuberculosis	Lung-tuberculosis-MAPSIM-tiles.zip	4.8 GB
	Lung-tuberculosis-WF-tiles.zip	4.6 GB
	Lung-tuberculosis-MAPSIM-stitch.tif	3.8 GB
	Lung-tuberculosis-WF-stitch.tif	3.6 GB
	Lung-tuberculosis-Color-stitch.tif	341 MB

Abbreviations

Av Int Proj, average intensity projection; FOV, field of view; H&E, hematoxylin and eosin; ICE, Image Composite Editor; MAP-SIM, maximum *a posteriori* probability SIM; NA, numerical aperture; LCOS, liquid crystal on silicon; PSDca, circularly averaged power spectral density; SIM, structured illumination microscopy; WF, wide field.

Ethics approval and consent to participate

Because the samples were acquired commercially, and because they are completely de-identified (meaning that there is no way to connect these particular samples to the original donor), this is not considered human subject research, and approval is not required to work with these samples.

Consent for publication

Not applicable

Competing interests

The authors declare that they have no competing interests.

Funding

Research reported in this publication was supported by the National Institute of General Medical Sciences of the National Institutes of Health under award number 1R15GM128166-01. This work was also supported by the UCCS BioFrontiers Center. The funding sources had no involvement in study design; in the collection, analysis and interpretation of data; in the writing of the report; or in the decision to submit the article for publication. This material is based in part upon work supported by the National Science Foundation under Grant Number 1727033. Any opinions, findings, and conclusions or recommendations expressed in this material are those of the authors and do not necessarily reflect the views of the National Science Foundation.

Author Contributions

KJ: acquired data, analyzed data, wrote the paper

GH: conceived project, acquired data, analyzed data, supervised research, wrote the paper

References

1. M. A. A. Neil, R. Juškaitis, and T. Wilson, "Method of obtaining optical sectioning by using structured light in a conventional microscope," Opt. Lett. **22**, 1905–1907 (1997).
2. M. G. L. Gustafsson, "Surpassing the lateral resolution limit by a factor of two using structured illumination microscopy," J. Microsc. **198**, 82–87 (2000).
3. R. Heintzmann and C. Cremer, "Laterally modulated excitation microscopy: improvement of resolution by using a diffraction grating," Proc. SPIE **3568**, 185–196 (1998).
4. L. Schermelleh, P. M. Carlton, S. Haase, L. Shao, L. Winoto, P. Kner, B. Burke, M. C. Cardoso, D. A. Agard, M. G. L. Gustafsson, H. Leonhardt, and J. W. Sedat, "Subdiffraction multicolor imaging of the nuclear periphery with 3D structured illumination microscopy," Science **320**, 1332–1336 (2008).
5. R. Fiolka, L. Shao, E. H. Rego, M. W. Davidson, and M. G. L. Gustafsson, "Time-lapse two-color 3D imaging of live cells with doubled resolution using structured illumination," Proc. Natl. Acad. Sci. U. S. A. **109**, 5311–5315 (2012).
6. J. Pospíšil, T. Lukeš, J. Bendesky, K. Fliegel, K. Spendier, and G. M. Hagen, "Imaging tissues and cells beyond the diffraction limit with structured illumination microscopy and Bayesian image reconstruction," Gigascience **8**, giy126 (2019).
7. X. Huang, J. Fan, L. Li, H. Liu, R. Wu, Y. Wu, L. Wei, H. Mao, A. Lal, P. Xi, L. Tang, Y. Zhang, Y. Liu, S.

Tan, and L. Chen, "Fast, long-term, super-resolution imaging with Hessian structured illumination microscopy," Nat. Biotechnol. **36**, 451–459 (2018).

8. M. G. L. Gustafsson, L. Shao, P. M. Carlton, C. J. R. Wang, I. N. Golubovskaya, W. Z. Cande, D. A. Agard, and J. W. Sedat, "Three-dimensional resolution doubling in widefield fluorescence microscopy by structured illumination," Biophys. J. **94**, 4957–4970 (2008).
9. S. Rossberger, G. Best, D. Baddeley, R. Heintzmann, U. Birk, S. Dithmar, and C. Cremer, "Combination of structured illumination and single molecule localization microscopy in one setup," J. Opt. **15**, 094003 (2013).
10. P. Křížek, I. Raška, and G. M. Hagen, "Flexible structured illumination microscope with a programmable illumination array," Opt. Express **20**, 24585 (2012).
11. P. Kner, B. B. Chhun, E. R. Griffis, L. Winoto, and M. G. L. Gustafsson, "Super-resolution video microscopy of live cells by structured illumination," Nat. Methods **6**, 339–342 (2009).
12. L. J. Young, F. Ströhl, and C. F. Kaminski, "A Guide to Structured Illumination TIRF Microscopy at High Speed with Multiple Colors," J. Vis. Exp. e53988–e53988 (2016).
13. V. Poher, H. X. Zhang, G. T. Kennedy, C. Griffin, S. Oddos, E. Gu, D. S. Elson, M. Girkin, P. M. W. French, M. D. Dawson, and M. A. Neil, "Optical sectioning microscope with no moving parts using a micro-stripe array light emitting diode," Opt. Express **15**, 11196–11206 (2007).
14. F. Orieux, E. Sepulveda, V. Loriette, B. Dubertret, and J.-C. Olivo-Marin, "Bayesian estimation for optimized structured illumination microscopy.," IEEE Trans. Image Process. **21**, 601–14 (2012).
15. T. Lukeš, P. Křížek, Z. Švindrych, J. Benda, M. Ovesný, K. Fliegel, M. Klíma, and G. M. Hagen, "Three-dimensional super-resolution structured illumination microscopy with maximum a posteriori probability image estimation," Opt. Express **22**, 29805–17 (2014).
16. T. Lukeš, G. M. Hagen, P. Křížek, Z. Švindrych, K. Fliegel, and M. Klíma, "Comparison of image reconstruction methods for structured illumination microscopy," Proc. SPIE **9129**, 91293J (2014).
17. C. A. Werley, M.-P. Chien, and A. E. Cohen, "An ultrawidefield microscope for high-speed fluorescence imaging and targeted optogenetic stimulation," Biomed. Opt. Express **8**, 5794 (2017).
18. N. J. Sofroniew, D. Flickinger, J. King, and K. Svoboda, "A large field of view two-photon mesoscope with subcellular resolution for in vivo imaging," Elife **5**, (2016).

19. G. McConnell, J. Trägårdh, R. Amor, J. Dempster, E. Reid, and W. B. Amos, "A novel optical microscope for imaging large embryos and tissue volumes with sub-cellular resolution throughout," Elife **5**, (2016).

20. P. J. Keller, A. D. Schmidt, J. Wittbrodt, and E. H. K. Stelzer, "Reconstruction of zebrafish early embryonic development by scanned light sheet microscopy," Science **322**, 1065–1069 (2008).

21. J. N. Stirman, I. T. Smith, M. W. Kudenov, and S. L. Smith, "Wide field-of-view, multi-region, two-photon imaging of neuronal activity in the mammalian brain," Nat. Biotechnol. **34**, 857–862 (2016).

22. B. Migliori, M. S. Datta, C. Dupre, M. C. Apak, S. Asano, R. Gao, E. S. Boyden, O. Hermanson, R. Yuste, and R. Tomer, "Light sheet theta microscopy for rapid high-resolution imaging of large biological samples," BMC Biol. **16**, 57 (2018).

23. T. C. Schlichenmeyer, M. Wang, K. N. Elfer, and J. Q. Brown, "Video-rate structured illumination microscopy for high-throughput imaging of large tissue areas," Biomed. Opt. Express **5**, 366 (2014).

24. D. Xu, T. Jiang, A. Li, B. Hu, Z. Feng, H. Gong, S. Zeng, and Q. Luo, "Fast optical sectioning obtained by structured illumination microscopy using a digital mirror device," J. Biomed. Opt. **18**, 060503 (2013).

25. F. B. Legesse, O. Chernavskaia, S. Heuke, T. Bocklitz, T. Meyer, J. Popp, and R. Heintzmann, "Seamless stitching of tile scan microscope images," J. Microsc. **258**, 223–232 (2015).

26. S. Preibisch, S. Saalfeld, and P. Tomancak, "Globally optimal stitching of tiled 3D microscopic image acquisitions," Bioinformatics **25**, 1463–1465 (2009).

27. C. Murtin, C. Frindel, D. Rousseau, and K. Ito, "Image processing for precise three-dimensional registration and stitching of thick high-resolution laser-scanning microscopy image stacks," Comput. Biol. Med. **92**, 22–41 (2018).

28. J. Chalfoun, M. Majurski, T. Blattner, K. Bhadriraju, W. Keyrouz, P. Bajcsy, and M. Brady, "MIST: Accurate and Scalable Microscopy Image Stitching Tool with Stage Modeling and Error Minimization," Sci. Rep. **7**, 4988 (2017).

29. Y. Yu and H. Peng, "Automated high speed stitching of large 3D microscopic images," in *2011 IEEE International Symposium on Biomedical Imaging: From Nano to Macro* (IEEE, 2011), pp. 238–241.

30. A. Bria and G. Iannello, "TeraStitcher - A tool for fast automatic 3D-stitching of teravoxel-sized microscopy images," BMC Bioinformatics **13**, 316 (2012).

31. F. Yang, Z.-S. Deng, and Q.-H. Fan, "A method for fast automated microscope image stitching," Micron **48**,

17–25 (2013).

32. P. Thévenaz and M. Unser, "User-friendly semiautomated assembly of accurate image mosaics in microscopy," Microsc. Res. Tech. **70**, 135–146 (2007).

33. P. Křížek, T. Lukeš, M. Ovesný, K. Fliegel, and G. M. Hagen, "SIMToolbox: A MATLAB toolbox for structured illumination fluorescence microscopy," Bioinformatics **32**, 318–320 (2015).

34. Microsoft, "Image Composite Editor," .

35. C. A. Schneider, W. S. Rasband, and K. W. Eliceiri, "NIH Image to ImageJ: 25 years of image analysis," Nat. Methods **9**, 671–675 (2012).

36. M. Wang, D. B. Tulman, A. B. Sholl, H. Z. Kimbrell, S. H. Mandava, K. N. Elfer, S. Luethy, M. M. Maddox, W. Lai, B. R. Lee, and J. Q. Brown, "Gigapixel surface imaging of radical prostatectomy specimens for comprehensive detection of cancer-positive surgical margins using structured illumination microscopy," Sci. Rep. **6**, 27419 (2016).

37. J. L. Dobbs, H. Ding, A. P. Benveniste, H. M. Kuerer, S. Krishnamurthy, W. Yang, and R. Richards-Kortum, "Feasibility of confocal fluorescence microscopy for real-time evaluation of neoplasia in fresh human breast tissue," J. Biomed. Opt. **18**, 106016 (2013).

38. D. S. Gareau, "Feasibility of digitally stained multimodal confocal mosaics to simulate histopathology," J. Biomed. Opt. **14**, 034050 (2009).

39. M. T. Tilli, M. C. Cabrera, A. R. Parrish, K. M. Torre, M. K. Sidawy, A. L. Gallagher, E. Makariou, S. A. Polin, M. C. Liu, and P. A. Furth, "Real-time imaging and characterization of human breast tissue by reflectance confocal microscopy," J. Biomed. Opt. **12**, 051901 (2007).

40. A. Parrish, E. Halama, M. T. Tilli, M. Freedman, and P. A. Furth, "Reflectance confocal microscopy for characterization of mammary ductal structures and development of neoplasia in genetically engineered mouse models of breast cancer," J. Biomed. Opt. **10**, 051602 (2005).

41. Y. Chen, W. Xie, A. K. Glaser, N. P. Reder, C. Mao, S. M. Dintzis, J. C. Vaughan, and J. T. C. Liu, "Rapid pathology of lumpectomy margins with open-top light-sheet (OTLS) microscopy," Biomed. Opt. Express **10**, 1257 (2019).

42. J. Dobbs, S. Krishnamurthy, M. Kyrish, A. P. Benveniste, W. Yang, and R. Richards-Kortum, "Confocal fluorescence microscopy for rapid evaluation of invasive tumor cellularity of inflammatory breast carcinoma

core needle biopsies," Breast Cancer Res. Treat. **149**, 303–310 (2015).

43. G. M. Hagen, W. Caarls, K. A. Lidke, A. H. B. De Vries, C. Fritsch, B. G. Barisas, D. J. Arndt-Jovin, and T. M. Jovin, "Fluorescence recovery after photobleaching and photoconversion in multiple arbitrary regions of interest using a programmable array microscope," Microsc. Res. Tech. **72**, 431–440 (2009).

44. S. R. Kantelhardt, W. Caarls, A. H. B. de Vries, G. M. Hagen, T. M. Jovin, W. Schulz-Schaeffer, V. Rohde, A. Giese, and D. J. Arndt-Jovin, "Specific visualization of glioma cells in living low-grade tumor tissue," PLoS One **5**, 1–11 (2010).

45. L. Shao, P. Kner, E. H. Rego, and M. G. L. Gustafsson, "Super-resolution 3D microscopy of live whole cells using structured illumination," Nat. Methods **8**, 1044–1046 (2011).

46. B.-C. Chen, W. R. Legant, K. Wang, L. Shao, D. E. Milkie, M. W. Davidson, C. Janetopoulos, X. S. Wu, J. A. Hammer, Z. Liu, B. P. English, Y. Mimori-Kiyosue, D. P. Romero, A. T. Ritter, J. Lippincott-Schwartz, L. Fritz-Laylin, R. D. Mullins, D. M. Mitchell, J. N. Bembenek, A.-C. Reymann, R. Bohme, S. W. Grill, J. T. Wang, G. Seydoux, U. S. Tulu, D. P. Kiehart, and E. Betzig, "Lattice light-sheet microscopy: Imaging molecules to embryos at high spatiotemporal resolution," Science **346**, 1257998 (2014).

47. G. M. Hagen, W. Caarls, M. Thomas, A. Hill, K. A. Lidke, B. Rieger, C. Fritsch, B. van Geest, T. M. Jovin, and D. J. Arndt-Jovin, "Biological applications of an LCoS-based programmable array microscope," Proc. SPIE **64410S**, 1–12 (2007).

48. H. Hurwitz, "Entropy reduction in Bayesian analysis of measurements," Phys. Rev. A **12**, 698–706 (1975).

49. P. J. Verveer and T. M. Jovin, *Efficient Superresolution Restoration Algorithms Using Maximum a Posteriori Estimations with Application to Fluorescence Microscopy* (1997), Vol. 14, p. 1696.

50. Michael Schmid, "Fast Filters," . https://imagej.nih.gov/ij/plugins/download/misc/Fast_Filters.java

51. J. Pospíšil, K. Fliegel, and M. Klíma, "Assessing resolution in live cell structured illumination microscopy," in *Proceedings of SPIE - The International Society for Optical Engineering*, P. Páta and K. Fliegel, eds. (SPIE, 2017), Vol. 10603, p. 39.

52. Johnson KA; Hagen GM (2020): Supporting data for "Artifact-free whole-slide imaging with structured illumination microscopy and Bayesian image reconstruction" GigaScience Database. http://dx.doi.org/10.5524/100728

53 T. C. Schlichenmeyer, M. Wang, C. Wenk, J. Q. Brown, and J. Q. Brown, "Autofocus optimization for

tracking tissue surface topography in large-area mosaicking structured illumination microscopy," in *Frontiers in Optics 2014* (OSA, 2014), p. FM4F.3.

54. H. L. Fu, J. L. Mueller, M. P. Javid, J. K. Mito, D. G. Kirsch, N. Ramanujam, and J. Q. Brown, "Optimization of a Widefield Structured Illumination Microscope for Non-Destructive Assessment and Quantification of Nuclear Features in Tumor Margins of a Primary Mouse Model of Sarcoma," PLoS One **8**, e68868 (2013).

55. H. L. Fu, J. L. Mueller, M. J. Whitley, D. M. Cardona, R. M. Willett, D. G. Kirsch, J. Q. Brown, and N. Ramanujam, "Structured Illumination Microscopy and a Quantitative Image Analysis for the Detection of Positive Margins in a Pre-Clinical Genetically Engineered Mouse Model of Sarcoma," PLoS One **11**, e0147006 (2016).

FIGURE CAPTIONS

Figure 1: Simplified diagram of SIM system. LCOS, liquid crystal on silicon

Figure 2. Simplified connection diagram.

Figure 3. Illumination trigger signal logic. This logical operation occurs for each illumination wavelength at the rightmost AND gates pictured in Figure 2.

Figure 4: Edge handling during the blurring process. (a) is the result after performing an average intensity projection on a set of MAP-SIM tiles. (b) shows (a) after being blurred using ImageJ's 'Gaussian blur' with a radius of 200 pixels, while (c) shows (a) blurred using the 'border limited mean' filter.

Figure 5: Vignetting artifacts and their removal. (a) shows the result of stitching images without applying the devignetting process, while (b) shows a stitch of the same data after devignetting has been applied. (c) shows the average intensity projection of the images used to stitch (a), which estimates the vignette profile of each frame. This estimate can be refined by application of an edge-limited blurring filter, as shown in (d). (e) shows the average intensity projection of the data used in (b), after devignetting has been applied. The uniform brightness of (e) indicates that no major vignetting artifacts remain in the devignetted data.

Figure 6. Panoramic SIM data processing workflow. Devignetting was performed after SIM reconstruction. Note the vignette profile differs between reconstruction methods, necessitating separate projection, blurring and division steps. Av Int Proj, average intensity projection.

Figure 7. Evaluating image resolution. (a) and (b) show a tile from the data in Figure 10 (basal cell carcinoma sample) after widefield and MAP-SIM reconstruction, respectively. (c) and (d) each show a zoom-in of (a) and (b), respectively. (e) and (f) each show the FFT of (a) and (b), respectively. The dotted lines in (e) and (f) indicate the resolution of each image according to the resolution measurement described.

Figure 8. Normalized, radially averaged power spectral density (PSD_{ca}) and resolution analysis measured on the tiles shown in Figs. 7a and 7b.

Figure 9: Carcinoma of human prostate. (a) Color overview, (b) WF stitch, (c) MAP-SIM stich. (d) shows a region of the sample indicated in (a). (e) and (f) each show a zoom-in of (b) and (c), respectively, in the region indicated in (a).

Figure 10: Basal Cell Carcinoma. (a) Color overview, (b) WF stitch, (c) MAP-SIM stich. (d) shows a region of the sample indicated in (a). (e) and (f) each show a zoom-in of (b) and (c), respectively, in the region indicated in (a).

Figure 11: Adenocarcinoma of human ovary. (a) Color overview, (b) WF stitch, (c) MAP-SIM stich. (d), (g) show a region of the sample indicated in (a), acquired separately from (a) using a 10× objective. (e) and (h) show a zoom-in of (b), while (f) and (i) show a zoom-in of (c), all in the regions indicated in (a).

Figure 12: Adenocarcinoma of human breast. (a) Color overview, (b) WF stitch, (c) MAP-SIM stich. (d) shows a region of the sample indicated in (a), acquired separately from (a) using a 10× objective. (e) and (f) each show a zoom-in of (b) and (c), respectively, in the region indicated in (a).

Figure 13: Tuberculosis of human lung. (a) Color overview, (b) WF stitch, (c) MAP-SIM stich. (d) shows a region of the sample indicated in (a), acquired separately from (a) using a 20× objective. (e) and (f) each show a zoom-in of (b) and (c), respectively, in the region indicated in (a).

Figure 14: Analysis of periodic stitching artifacts in the frequency domain. (a) shows a stitched image where devignetting has not been applied to the tiles, while (b) shows a stitch of devignetted data. (c) and (d) show the

central region of the FFT's of (a) and (c), respectively. The arrows in (c) point to the fundamental peaks of the grid artifact. (e) shows plots of (c) and (d) through the center of each image along the x and y axes. The calculated value for T_{stitch} aligns very well with the peaks along both axes.

Click here to access/download;Figure;Figure14-analysis.png

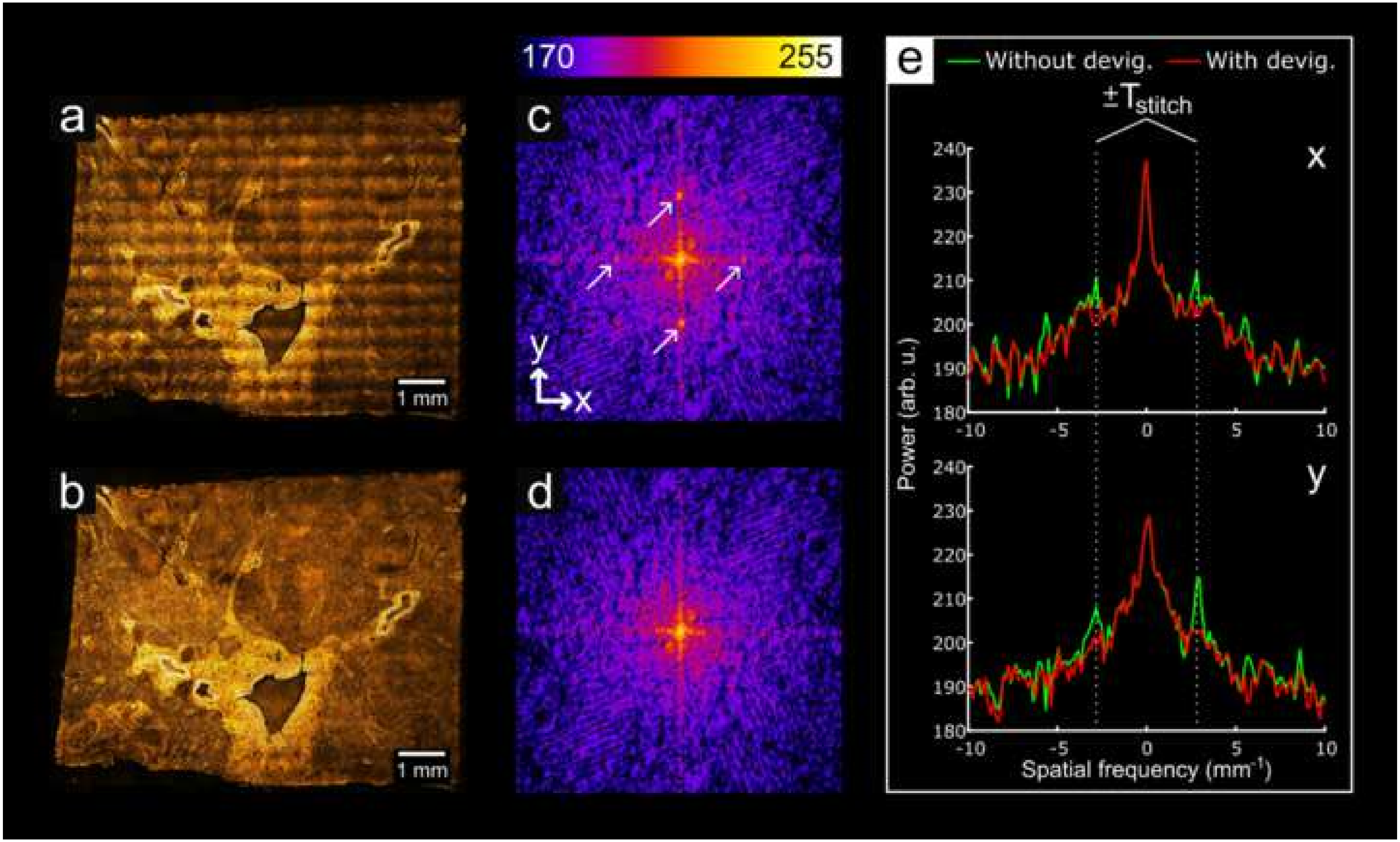

Click here to access/download;Figure;Figure13-lung.png

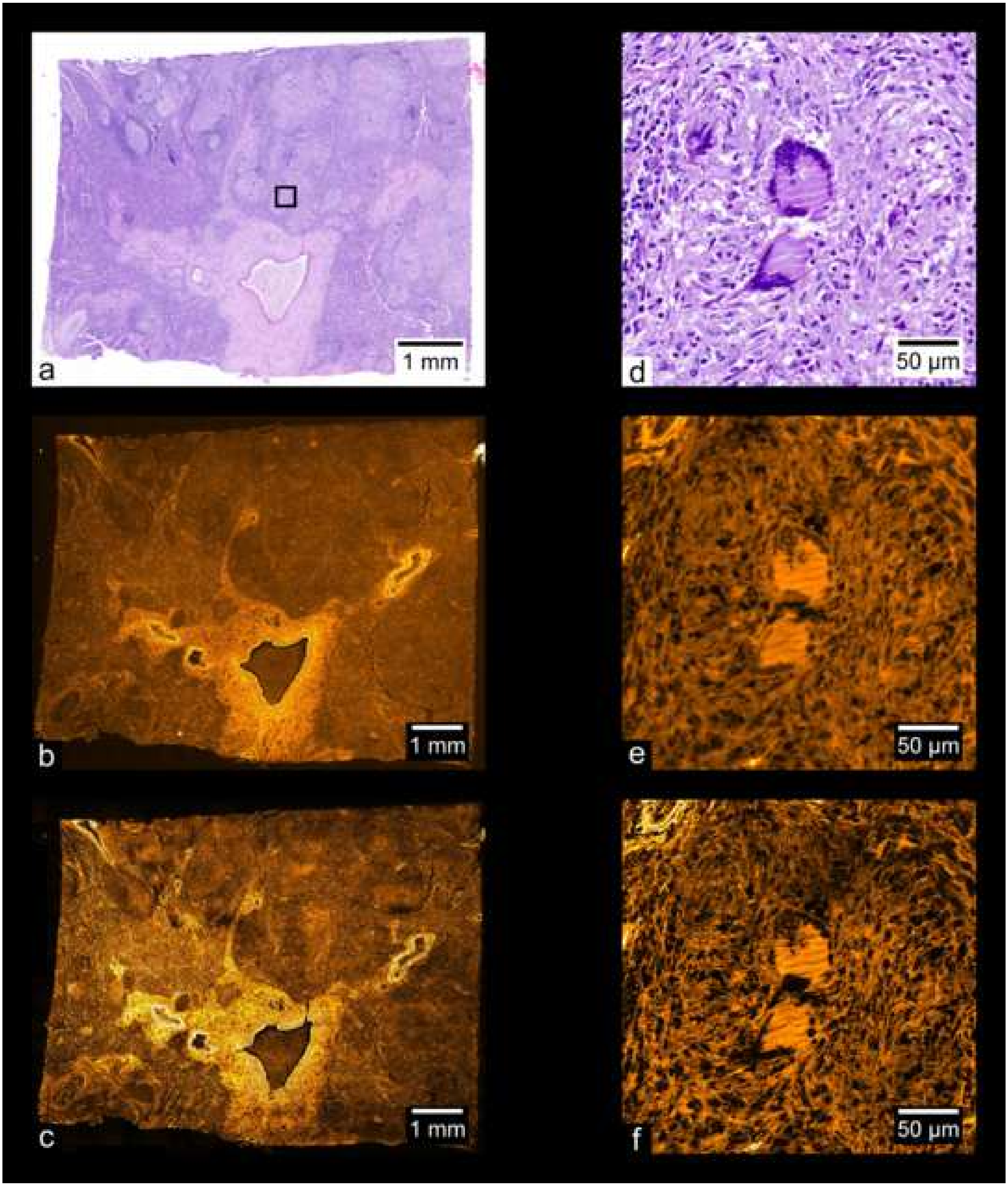

Figure12-breast

Click here to access/download;Figure;Figure12-breast.png

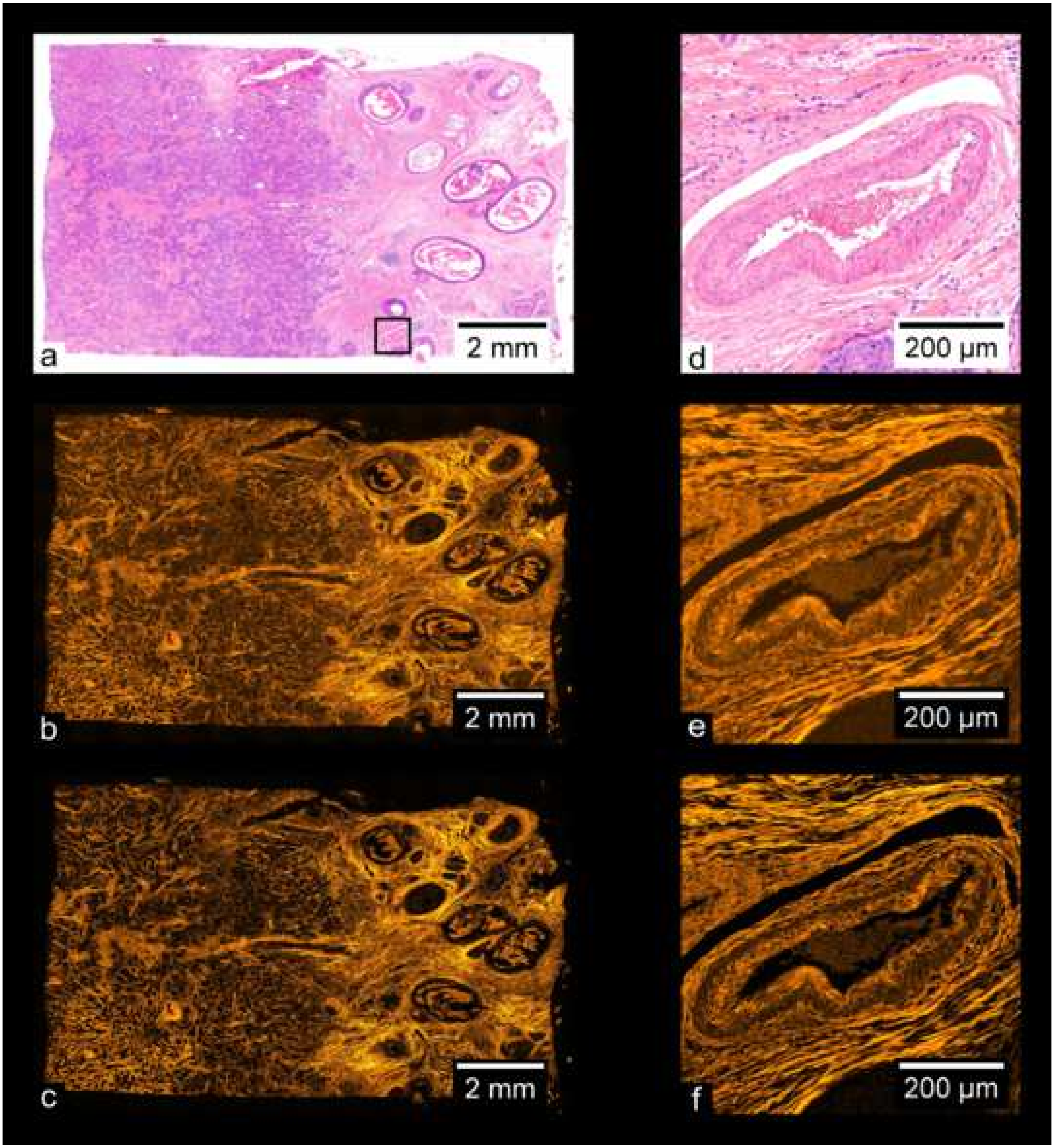

Click here to access/download;Figure;Figure11-ovary.png

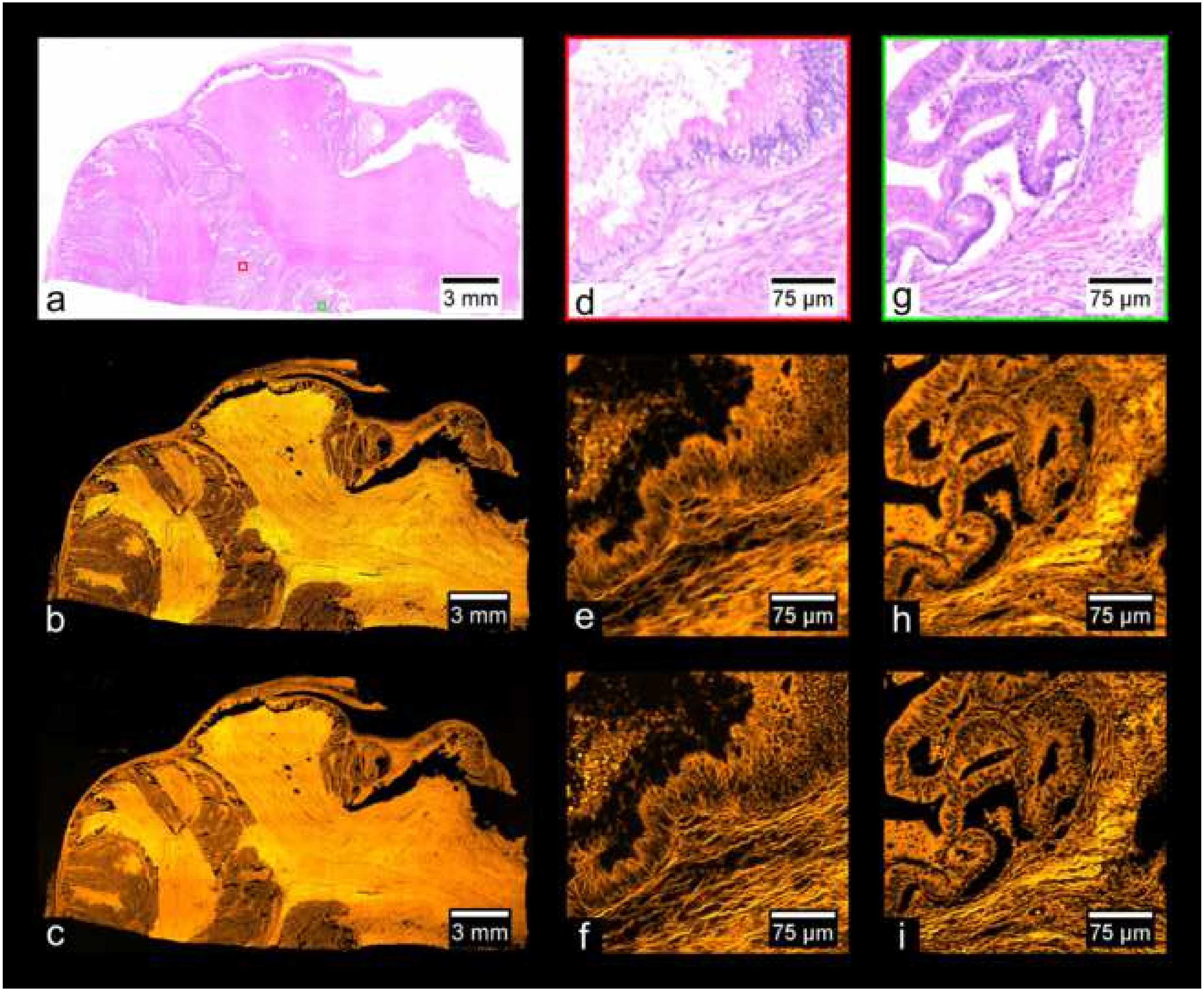

Figure10-basal cell

Click here to access/download;Figure;Figure10-basal cell.png

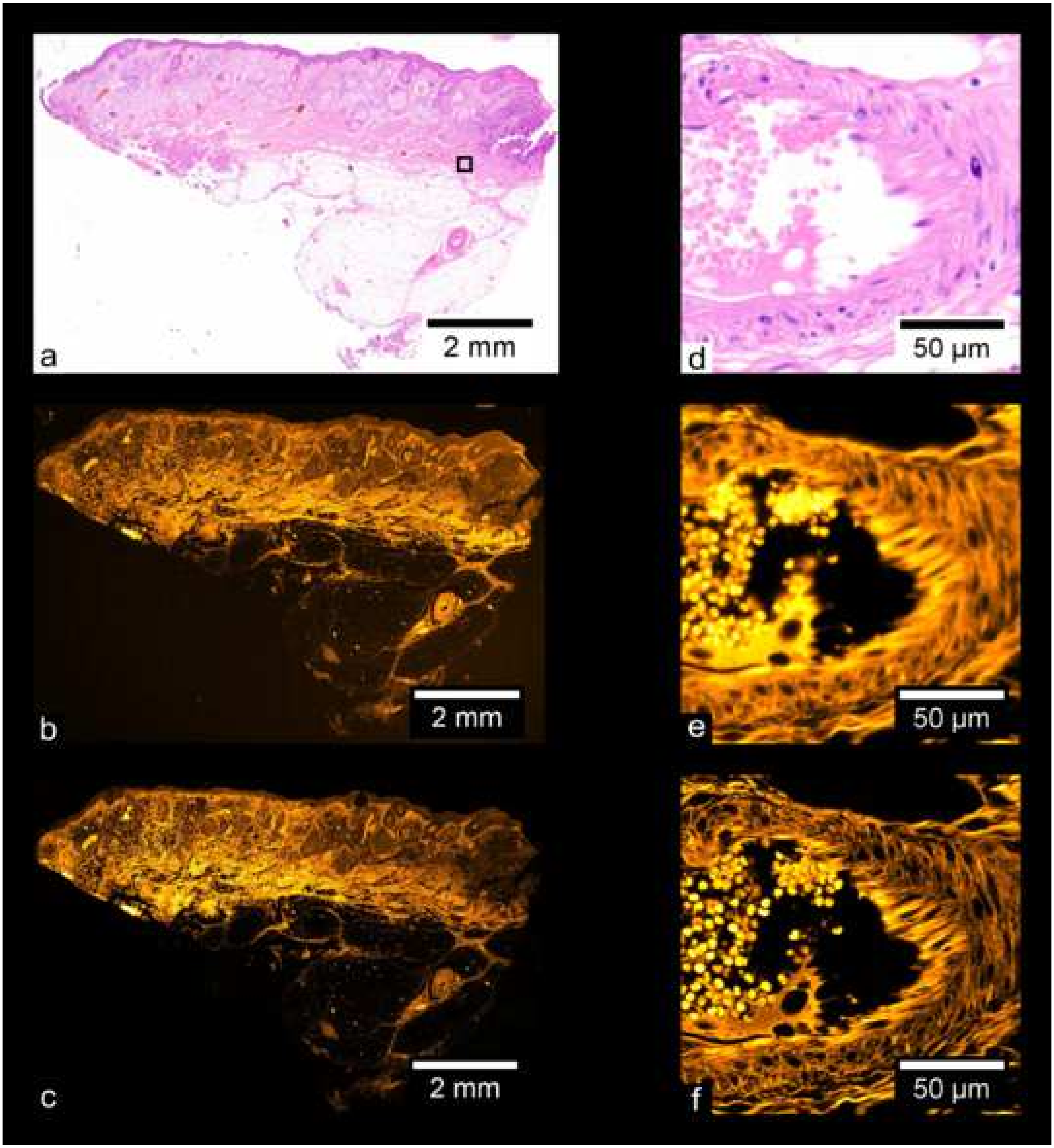

 Click here to access/download;Figure;Figure09-prostate.png

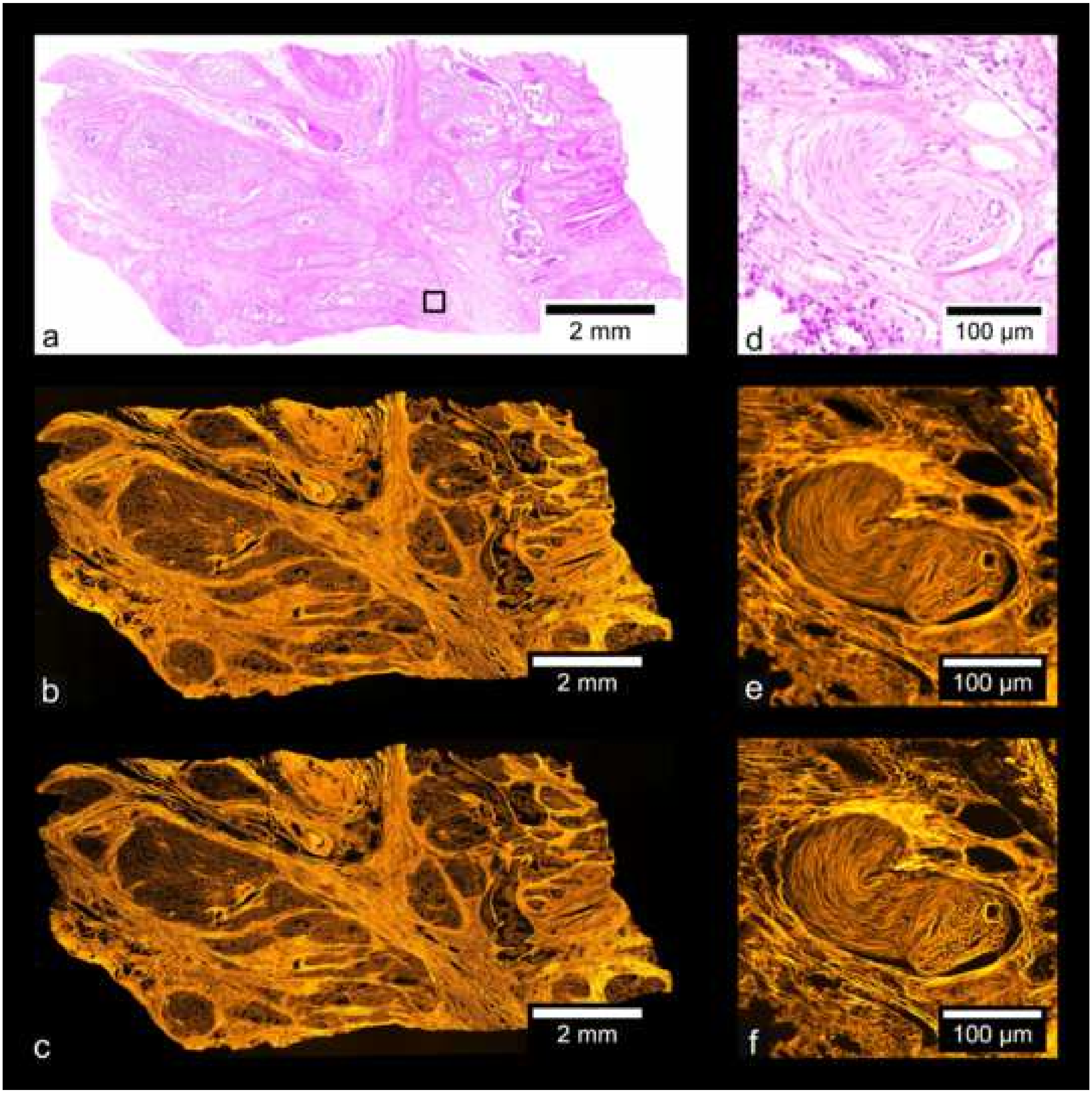

Figure08-PSD

Click here to access/download;Figure;Figure08-PSD.png

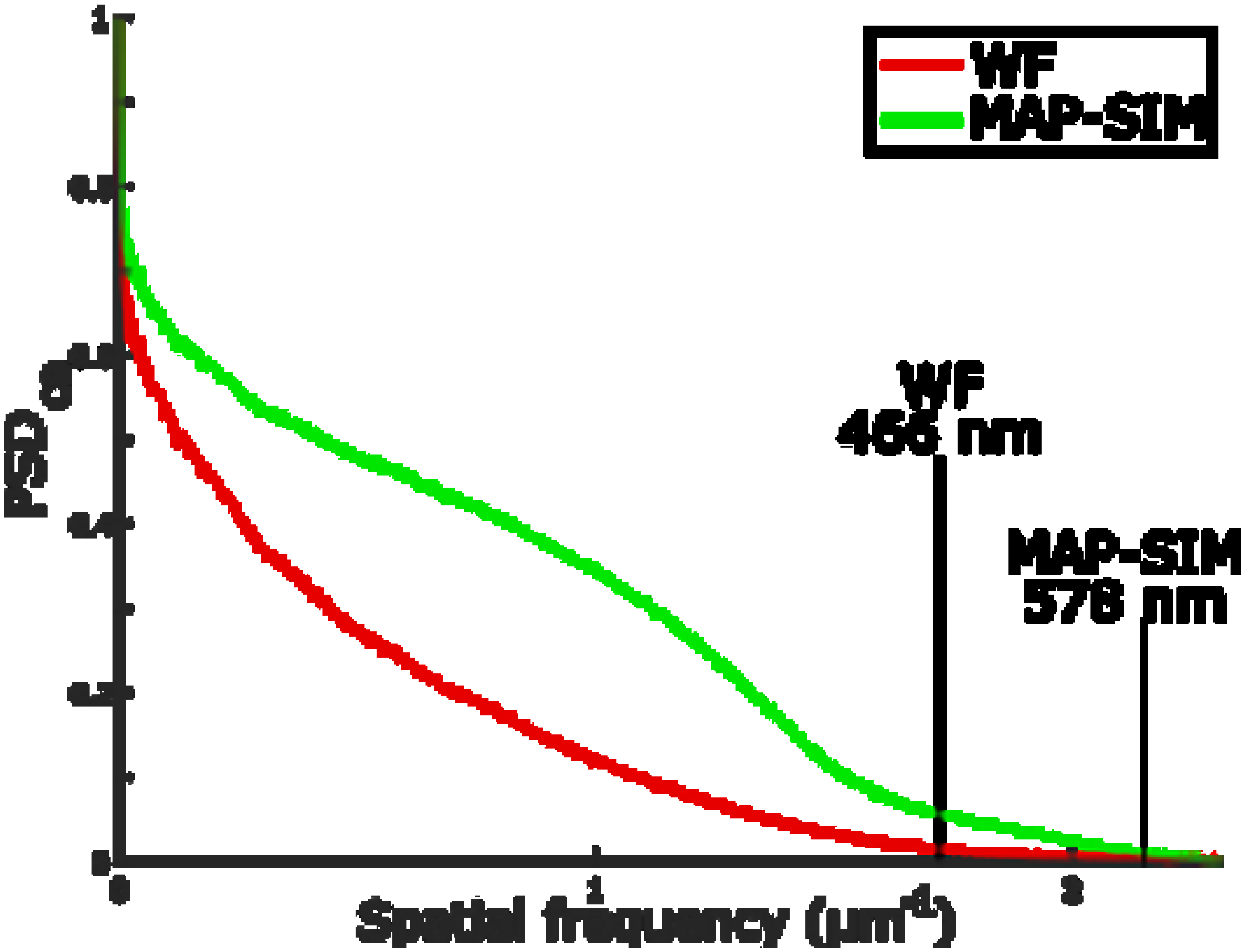

 Click here to access/download;Figure;Figure07-resolution.png

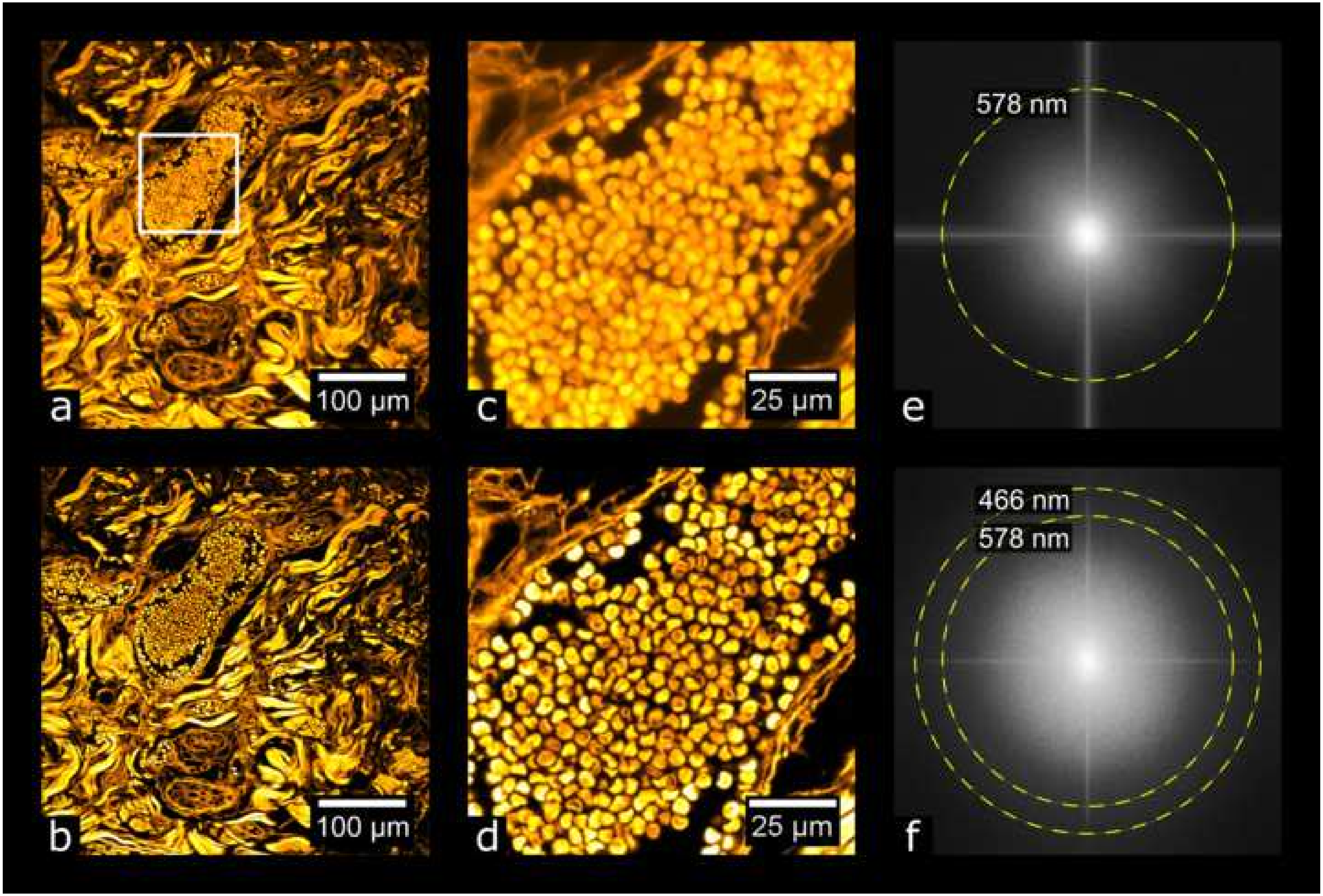

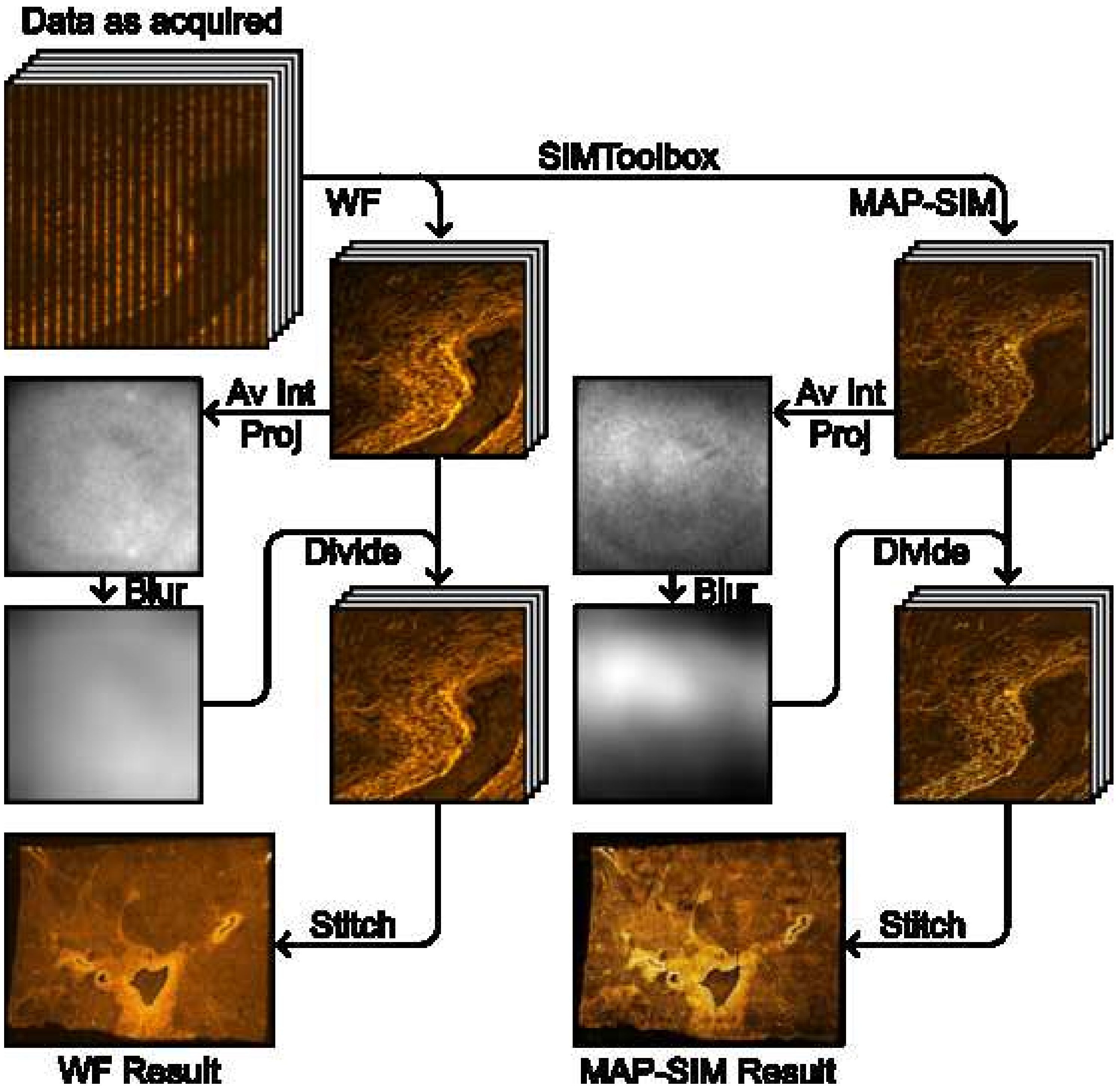
Data as acquired
SIMToolbox
WF
MAP-SIM
Av Int Proj
Av Int Proj
Divide
Divide
Blur
Blur
Stitch
Stitch
WF Result
MAP-SIM Result

Figure05-devignetting

Click here to access/download;Figure;Figure05-devignetting.png

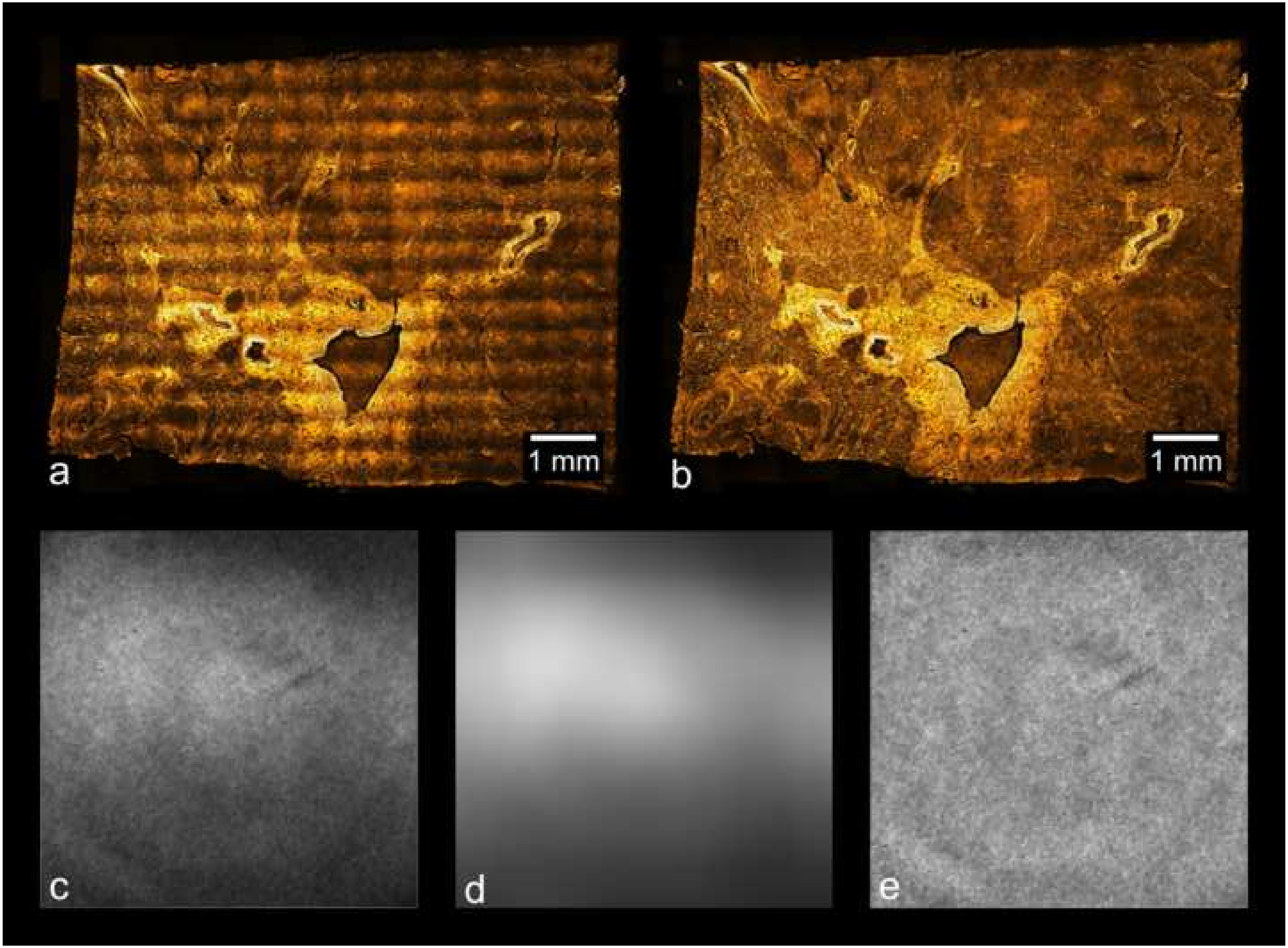

Click here to access/download;Figure;Figure04-edge method.png

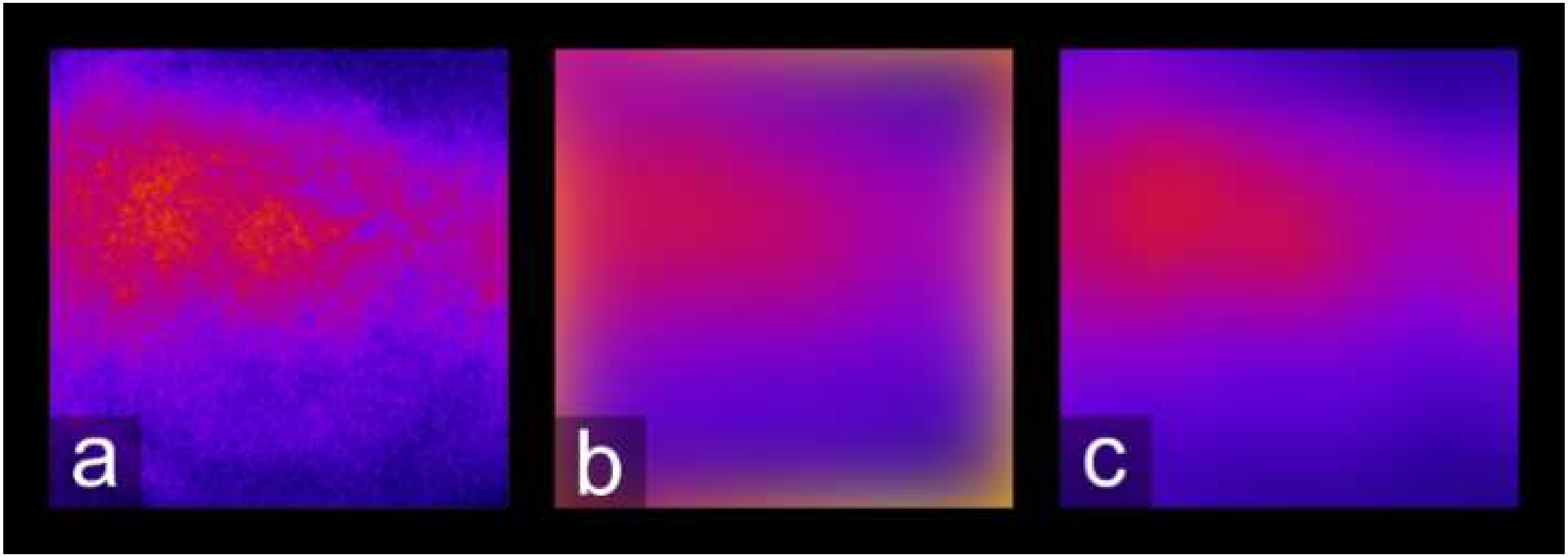

Figure03-trigger
Click here to access/download;Figure;Figure03-trigger.png

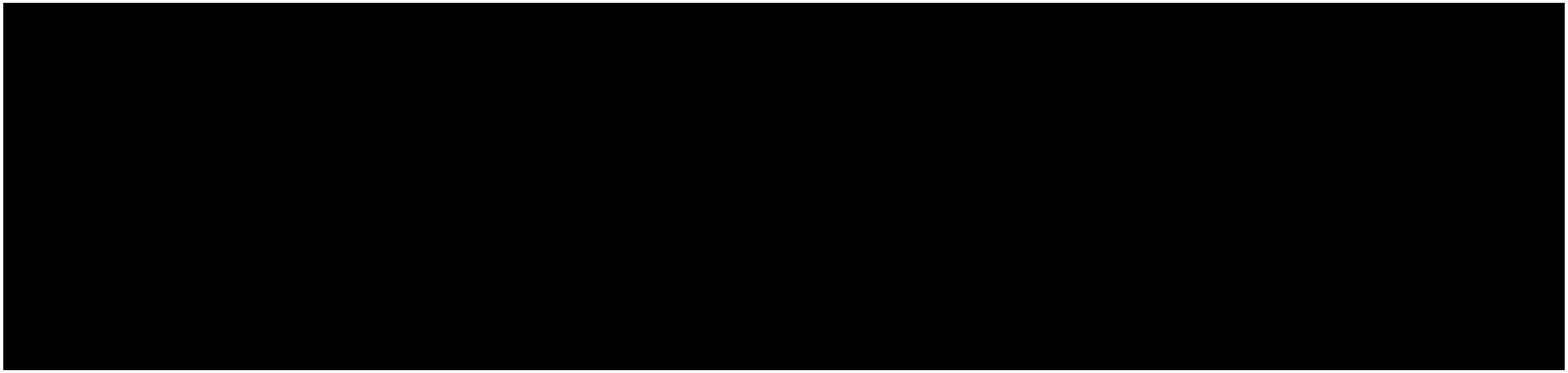

Figure02-connection diagram

Click here to access/download;Figure;Figure02-connection diagram.png

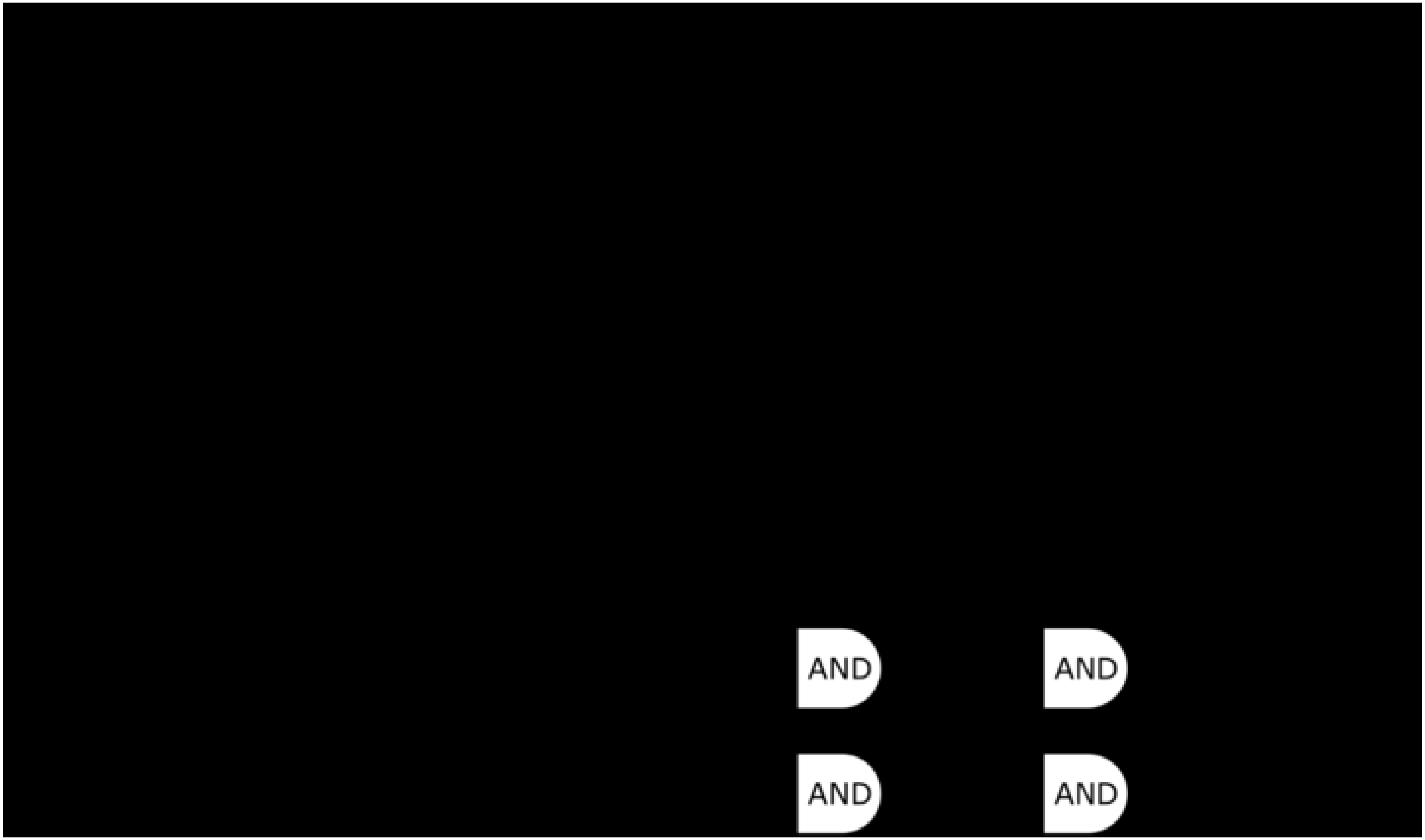

Click here to access/download;Figure;Figure01-system diagram.png

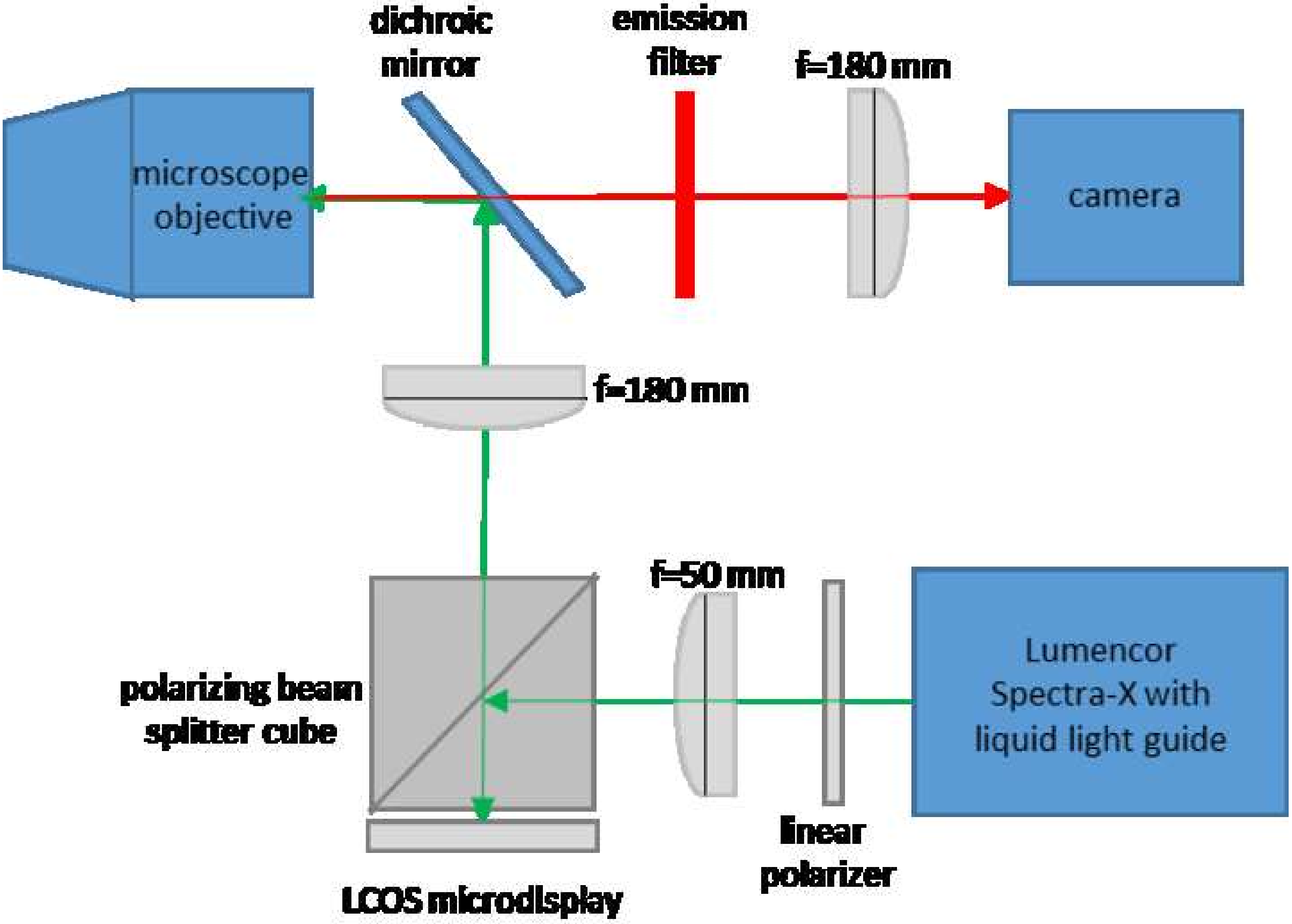

Manuscript

Click here to access/download;Personal Cover;Hagen letter to editor final submission.docx

University of Colorado Colorado Springs

Dr. Guy M. Hagen

Biofrontiers

1420 Austin Bluffs Pkwy.

Colorado Springs, CO 80918

Tel. 719-255-3692

ghagen@uccs.edu

Dear Editor:

We would like to re-submit our manuscript entitled "Artifact-free whole-slide imaging with structured illumination microscopy and Bayesian image reconstruction" for consideration in *GigaScience* as a data note. We made the following revisions.

1. The reviewer noticed that two of the data files was incomplete or corrupted. We reloaded these two files.

2. We included a table showing the organization of the data files along with their sizes.

3. We included a universal public domain dedication text file with the dataset.

4. We added a statement about ethics approval and consent to participate.

5. We moved all of the information from the supplement into the main paper. We removed the example of the cellular-scale imaging.

We hope that our paper will now be acceptable for publication in GigaScience.

Sincerely,

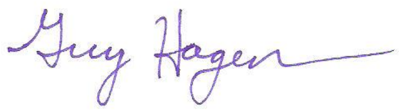

Guy M. Hagen
