# Supplementary material for: Artifact-free whole-slide imaging with structured illumination microscopy and Bayesian image reconstruction
Source: Gigascience. 2020 Apr 14;9(4):giaa035. doi: 10.1093/gigascience/giaa035 (PMC7155289; doi:10.1093/gigascience/giaa035)
Supplement: giaa035_Response_to_Reviewer_Comments_Original_Submission [file giaa035_response_to_reviewer_comments_original_submission.pdf]

Author's Response To Reviewer Comments

Close

Dear Editor:

We would like to re-submit our manuscript entitled "Artifact-free whole-slide imaging with structured illumination microscopy and Bayesian image reconstruction" for consideration in GigaScience as a data note. We would like to thank the reviewers for their comments about the paper. Reviewer 1 was very enthusiastic about the paper and we appreciate these positive comments about our work. Reviewer 2 was also positive, but had a few comments about the paper which were concerned with the organization of the paper and the data.

1. The first comment was that we should upload all of the un-stitched images, and to provide them as individual image files rather than as a single large file. This has been accomplished and now all of the raw and processed data is available at GigaDB. We packed all of the tiles for a given sample into ZIP files so that users will be able to download all of the tiles as a single large file. Otherwise users would have to click and download each file separately. GigaDB will probably re-package these files into a different format such as TAR.

2. The reviewer noted that we did not include certain information about the actual dataset in the main paper. For example, the directory structure, file sizes and types, etc. This information is not normally included in GigaScience articles which I have read. The GigaScience Instructions to Authors do not require this information. This information will be present on the GigaDB page for this dataset. There will be a table, generated by GigaDB, which will contain the desired information.

3. The reviewer noted that an open access dedication should be included with the dataset. This has been done.

4. The reviewer noted that, because the tissue preparations are of human origin, that information about ethics and consent should not be overlooked. Thank you for reminding us of this important point. However, because the samples were obtained commercially, it is the responsibility of the supplying company to ensure that ethical and legal guidelines are followed. I have double checked this with the Institutional Review Board (IRB) here at the University of Colorado, Colorado Springs. Because the samples are acquired commercially, and because they are completely de-identified (meaning that there is no way to connect these particular samples to the original donor), this is not considered human subject research, and approval is not required to work with these samples.

5, 6. In points 5 and 6, the reviewer is asking us to rearrange the paper by putting the items in the supplementary information into the main paper, and then to eliminate figures 7-10. Respectfully, we do not plan to do this for several reasons. I feel that reorganizing the manuscript as suggested would not improve the paper.

The whole point of the paper is to show the results of our research, in this case the results are the final, high resolution stitched images of the samples we examined. Eliminating these results from the paper would not be a good idea. For example, people working on breast cancer will be interested in the imaged breast cancer sample, people working on prostate cancer will be interested in the imaged prostate cancer sample, and so on. Further, the data re-use section was included in the supplementary material in our previous two papers in GigaScience. I believe this is the appropriate place for this information. Most GigaScience articles I have read do not include an actual, concrete example of data re-use like we do, and so this is a strength of our paper. Not everything can go into the main paper, and it is common practice today to publish supplementary information with additional experimental details, which can sometimes be quite lengthy.
Section 3 of the supplementary information is there for a specific reason. Almost all current research in structured illumination microscopy is performed on single cells using high magnification objectives. In the current paper we are imaging tissues over large areas, which is a quite different application. It is important for readers to realize that the methods presented here are widely applicable, including in the

more typical application of SIM. Section 3 of the supplement is aimed at other people involved in the SIM field.

7. The structured illumination data processing steps are the same as were used in our previous publications. We noted this in the section ‘SIM data processing’ by stating that the SIM reconstructions were performed in the same was as previously described. What is new here is the image devignetting and stitching methods applied to microscopy images of this type.
The steps described in the flow chart in Fig 3 are already described in the text. For example in the section ‘SIM data processing’ we state “SIM reconstructions were performed using SIMToolbox…” and “We generated optically sectioned, enhanced resolution images using… MAP-SIM.” In the section ‘Vignetting correction’ we state “Following SIM reconstruction, …We performed vignette removal by dividing each tile of the mosaic by an image representing the vignetting profile common to all tiles.” These are exactly the steps shown in the flowchart.

8. The reviewer noted that the quality of figures 1, 3, and 5 is very low. This is perfectly true, in the PDF file they look absolutely terrible and it is very disappointing. However the problem is with the PDF conversion process used by GigaScience. This is not something that authors can change. Please click on the links embedded in the PDF (in the upper right corner of the pages containing the figures) to download the original high resolution files for the figures. You will see that they are of high quality.

We hope that our paper will now be acceptable for publication in GigaScience.

Sincerely,

Guy M. Hagen

Close
