# Supplementary material for: Artifact-free whole-slide imaging with structured illumination microscopy and Bayesian image reconstruction
Source: Gigascience. 2020 Apr 14;9(4):giaa035. doi: 10.1093/gigascience/giaa035 (PMC7155289; doi:10.1093/gigascience/giaa035)
Supplement: giaa035_Reviewer_1_Report_Original_Submission — Chris Armit -- 9/18/2019 Reviewed [file giaa035_reviewer_1_report_original_submission.pdf]

Reviewer Report

Title: Artifact-free whole-slide imaging with structured illumination microscopy and Bayesian image reconstruction

Version: Original Submission **Date:** 9/18/2019

Reviewer name: Chris Armit

Reviewer Comments to Author:

This Data Note describes artifact-free whole-slide imaging with Structured Illumination Microscopy (SIM) and utilises a novel devignetting solution to stitch individual images together. The manuscript is well written and the supporting cellular-resolution image data is of high quality. It is noteworthy that the authors have published SIM data previously in GigaScience. However, whereas the latter study utilised cell line data, the data outlined in this newly submitted manuscript is clinically relevant as they represent human tissue samples derived from cancers of the prostate, skin, ovary, and breast. This new study additionally includes human tissue samples of tuberculosis of the lung. The tissue samples are obtained from a commercial source, and the authors detail the source company and product number, which is important for data provenance. It is additionally noteworthy that the SIMToolbox software used in this study is openly available and has been ascribed a GPLv3.0 license.

A major strength of this Data Note is the novel devignetting solution the authors utilise to improve image stitching. This is a known problem in whole-slide imaging, and I commend the authors for delivering artifact-free whole-slide images of human tissue samples. I see great value in the SIM datasets and I anticipate that they can be reused in several ways, for example by researchers wishing to explore whether machine-learning approaches can be used to annotate SIM images of human tissue in health and disease. In addition, and as the authors explicitly state in the Reuse potential section, "the unstitched image tiles provided in the dataset, which still contain vignetting artifacts, may be used to reproduce the results of our devignetting process, as well as to further develop more sophisticated devignetting approaches suited for SIM." I consider this an excellent point and I would strongly encourage GigaScience to archive both the unstitched image tiles and the processed image data outlined in this study.

Level of Interest

Please indicate how interesting you found the manuscript: Choose an item.

Quality of Written English

Please indicate the quality of language in the manuscript: Choose an item.

Declaration of Competing Interests

Please complete a declaration of competing interests, considering the following questions:

- Have you in the past five years received reimbursements, fees, funding, or salary from an organisation that may in any way gain or lose financially from the publication of this manuscript, either now or in the future?
- Do you hold any stocks or shares in an organisation that may in any way gain or lose financially from the publication of this manuscript, either now or in the future?
- Do you hold or are you currently applying for any patents relating to the content of the manuscript?
- Have you received reimbursements, fees, funding, or salary from an organization that holds or has applied for patents relating to the content of the manuscript?
- Do you have any other financial competing interests?
- Do you have any non-financial competing interests in relation to this paper?

If you can answer no to all of the above, write 'I declare that I have no competing interests' below. If your reply is yes to any, please give details below.

I declare that I have no competing interests.

I agree to the open peer review policy of the journal. I understand that my name will be included on my report to the authors and, if the manuscript is accepted for publication, my named report including any attachments I upload will be posted on the website along with the authors' responses. I agree for my report to be made available under an Open Access Creative Commons CC-BY license (http://creativecommons.org/licenses/by/4.0/). I understand that any comments which I do not wish to be included in my named report can be included as confidential comments to the editors, which will not be published.

Choose an item.

To further support our reviewers, we have joined with Publons, where you can gain additional credit to further highlight your hard work (see: https://publons.com/journal/530/gigascience). On publication of this paper, your review will be automatically added to Publons, you can then choose whether or not to claim your Publons credit. I understand this statement.

Yes Choose an item.
