# Supplementary material for: Artifact-free whole-slide imaging with structured illumination microscopy and Bayesian image reconstruction
Source: Gigascience. 2020 Apr 14;9(4):giaa035. doi: 10.1093/gigascience/giaa035 (PMC7155289; doi:10.1093/gigascience/giaa035)
Supplement: giaa035_Reviewer_2_Report_Revision_1 — Marcus Wagner -- 1/24/2020 Reviewed [file giaa035_reviewer_2_report_revision_1.pdf]

Reviewer Report

Title: Artifact-free whole-slide imaging with structured illumination microscopy and Bayesian image reconstruction

Version: Revision 1 **Date:** 1/24/2020

Reviewer name: Marcus Wagner

Reviewer Comments to Author:

Review of the revised manuscript GIGA-D-19-00306-R1, entitled
Artifact-free whole-slide imaging with structured illumination microscopy and Bayesian
image reconstruction,
by Johnson K, Hagen GM

Contents.
For a summary of the manuscript's contents, see my first review.
The dataset, to which the manuscript is related, is now (almost) complete. It consists now of five specimen, each containing two sets of unprocessed SIM image tiles (in WF and MAP-SIM mode) as large .zip files together with the finally stitched images in color overview, WF and MAP-SIM mode as the result of the described procedures.
Assessment of the manuscript.
Compared with its previous version, the manuscript was only slightly changed. The dataset is still incomplete (see below). Reviewer leaves the decision how to assess the manuscript in its current stage to the editor.
Objections from first review, authors' replies and reviewer's comments.
1) (Incompleteness of data and structure of the dataset).
-- Authors' reply: "The first comment was that we should upload all of the un-stitched images, and to provide them as individual image files rather than as a single large file. This has been accomplished and now all of the raw and processed data is available at GigaDB. We packed all of the tiles for a given sample into ZIP files so that users will be able to download all of the tiles as a single large file. Otherwise users would have to click and download each file separately."
-- Reviewer's comment: The structure of the dataset is now acceptable. Note, however, that in directory 05, both .zip files containing the tiles are corrupted and incomplete.
2) (Description of the dataset)
-- Authors' reply: "The reviewer noted that we did not include certain information about the actual dataset in the main paper. For example, the directory structure, file sizes and types, etc. This information is not normally included in GigaScience articles which I have read. The GigaScience Instructions to Authors do not require this information. This information will be present on the GigaDB page for this dataset. There will be a table, generated by GigaDB, which will contain the desired information."
-- Reviewer's comment: Content of the explanation file contained in the dataset should, in my view, still

become part of the manuscript.
3) (Missing CC0 Universal Public Domain Dedication)
-- Authors' reply: "The reviewer noted that an open access dedication should be included with the dataset. This has been done."
-- Reviewers's comment: CC0 Universal Public Domain Dedication has now been mentioned in the explanation file within the dataset. In order to make the dedication effective, however, the dedication text file itself must be included into the dataset.
4) (Ethics considerations for use of human tissue)
-- Authors' reply: "The reviewer noted that, because the tissue preparations are of human origin, that information about ethics and consent should not be overlooked. Thank you for reminding us of this important point. However, because the samples were obtained commercially, it is the responsibility of the supplying company to ensure that ethical and legal guidelines are followed. I have double checked this with the Institutional Review Board (IRB) here at the University of Colorado, Colorado Springs. Because the samples are acquired commercially, and because they are completely de-identified (meaning that there is no way to connect these particular samples to the original donor), this is not considered human subject research, and approval is not required to work with these samples."
-- Reviewer's comment: O.K. But note: The remarks made to the reviewer should be part of the paper, under "Ethics approval and consent to participate".
5) (Restructuring of the manuscript)
-- Authors' reply: "In points 5 and 6, the reviewer is asking us to rearrange the paper by putting the items in thesupplementary information into the main paper, and then to eliminate figures 7-10. Respectfully, we do not plan to do this for several reasons. I feel that reorganizing the manuscript as suggested would not improve the paper. (...) Further, the data re-use section was included in the supplementary material in our previous two papers in GigaScience. I believe this is the appropriate place for this information. Most GigaScience articles I have read do not include an actual, concrete example of data re-use like we do, and so this is a strength of our paper. Not everything can go into the main paper, and it is common practice today to publish supplementary information with additional experimental details, which can sometimes be quite lengthy.
Section 3 of the supplementary information is there for a specific reason. Almost all current research in structured illumination microscopy is performed on single cells using high magnification objectives. In the current paper we are imaging tissues over large areas, which is a quite different application. It is important for readers to realize that the methods presented here are widely applicable, including in the more typical application of SIM. Section 3 of the supplement is aimed at other people involved in the SIM field."
-- Reviewer's comment: I still disagree. Decision about this point is at the discretion of the editor.
6) (Reduction of number of figures)
-- Authors' reply: "The whole point of the paper is to show the results of our research, in this case the results are the final, high resolution stitched images of the samples we examined. Eliminating these results from the paper would not be a good idea. For example, people working on breast cancer will be interested in the imaged breast cancer sample, people working on prostate cancer will be interested in the imaged prostate cancer sample, and so on."
-- Reviewer's comment: O.K., this is a convincing reason to maintain the figures.

7) (Concordance of described processing steps and flowchart in Fig. 3).
-- Authors' reply: "The structured illumination data processing steps are the same as were used in our previous publications. We noted this in the section 'SIM data processing' by stating that the SIM reconstructions were performed in the same was as previously described. What is new here is the image devignetting and stitching methods applied to microscopy images of this type.
The steps described in the flow chart in Fig 3 are already described in the text. For example in the section 'SIM data processing' we state "SIM reconstructions were performed using SIMToolbox…" and "We generated optically sectioned, enhanced resolution images using… MAP-SIM." In the section 'Vignetting correction' we state "Following SIM reconstruction, …We performed vignette removal by dividing each tile of the mosaic by an image representing the vignetting profile common to all tiles." These are exactly the steps shown in the flowchart.
-- Reviewer's comment: Still the same objections as before. Decision about this point is at the discretion of the editor.
8) (Quality of figures 1, 3 and 5)
-- Authors' reply: "The reviewer noted that the quality of figures 1, 3, and 5 is very low. This is perfectly true, in the PDF file they look absolutely terrible and it is very disappointing. However the problem is with the PDF conversion process used by GigaScience. This is not something that authors can change. Please click on the links embedded in the PDF (in the upper right corner of the pages containing the figures) to download the original high resolution files for the figures. You will see that they are of high quality."
-- Reviewer's comment: O.K.
Marcus Wagner.

Level of Interest

Please indicate how interesting you found the manuscript: Choose an item.

Quality of Written English

Please indicate the quality of language in the manuscript: Choose an item.

Declaration of Competing Interests

Please complete a declaration of competing interests, considering the following questions:

- Have you in the past five years received reimbursements, fees, funding, or salary from an organisation that may in any way gain or lose financially from the publication of this manuscript, either now or in the future?
- Do you hold any stocks or shares in an organisation that may in any way gain or lose financially from the publication of this manuscript, either now or in the future?

- Do you hold or are you currently applying for any patents relating to the content of the manuscript?
- Have you received reimbursements, fees, funding, or salary from an organization that holds or has applied for patents relating to the content of the manuscript?
- Do you have any other financial competing interests?
- Do you have any non-financial competing interests in relation to this paper?

If you can answer no to all of the above, write 'I declare that I have no competing interests' below. If your reply is yes to any, please give details below.

I declare that I have no competing interests.

I agree to the open peer review policy of the journal. I understand that my name will be included on my report to the authors and, if the manuscript is accepted for publication, my named report including any attachments I upload will be posted on the website along with the authors' responses. I agree for my report to be made available under an Open Access Creative Commons CC-BY license (http://creativecommons.org/licenses/by/4.0/). I understand that any comments which I do not wish to be included in my named report can be included as confidential comments to the editors, which will not be published.

Choose an item.

To further support our reviewers, we have joined with Publons, where you can gain additional credit to further highlight your hard work (see: https://publons.com/journal/530/gigascience). On publication of this paper, your review will be automatically added to Publons, you can then choose whether or not to claim your Publons credit. I understand this statement.

Yes Choose an item.
