# Supplementary material for: Artifact-free whole-slide imaging with structured illumination microscopy and Bayesian image reconstruction
Source: Gigascience. 2020 Apr 14;9(4):giaa035. doi: 10.1093/gigascience/giaa035 (PMC7155289; doi:10.1093/gigascience/giaa035)
Supplement: giaa035_Authors_Response_To_Reviewer_Comments [file giaa035_authors_response_to_reviewer_comments.pdf]

Author's Response To Reviewer Comments

Close

Dear Editor:
We would like to re-submit our manuscript entitled “Artifact-free whole-slide imaging with structured illumination microscopy and Bayesian image reconstruction” for consideration in GigaScience as a data note. We made the following revisions.

1. The reviewer noticed that two of the data files was incomplete or corrupted. We reloaded these two files.

2. We included a table showing the organization of the data files along with their sizes.

3. We included a universal public domain dedication text file with the dataset.

4. We added a statement about ethics approval and consent to participate.

5. We moved all of the information from the supplement into the main paper. We removed the example of the cellular-scale imaging.

Please note that the figures in the automatically generated PDF look terrible as usual. Two of the figures look like blank black boxes. There is nothing I can do about this. Please click the links embedded in the PDF to view the figures.

We hope that our paper will now be acceptable for publication in GigaScience.

Sincerely,

Guy M. Hagen

Close
